# Targeting CD44 reverses sphingomyelin-induced oligodendrocyte maturation arrest in acid sphingomyelinase deficiency

**DOI:** 10.1038/s44321-026-00503-8

**Published:** 2026-08-12

**Authors:** Marta Guerrero-Valero, Elena Melgarejo, Miguel A Marchena, Beatriz Fernández-Gómez, Jaime Mulero-Franco, Fernando de Castro, Edward H Schuchman, María Dolores Ledesma

**Affiliations:** 1 Centro Biología Molecular Severo Ochoa (CSIC-UAM), Madrid, Spain; 2 Centro de Neurociencias Cajal/Instituto Cajal-CSIC, Madrid, Spain; 3 Universidad de Castilla-La Mancha, Ciudad Real, Spain; 4 Department of Genetics & Genomic Sciences, Icahn School of Medicine at Mount Sinai, New York, NY USA

**Keywords:** Neuroscience

## Abstract

Loss-of-function mutations in the *smpd1* gene cause acid sphingomyelinase deficiency (ASMD). Early neurodegeneration and lethality characterize its infantile neurovisceral form (type A). While neuronal dysfunction was traditionally considered the primary driver of the pathology, recent evidence suggests that dysmyelination and microgliosis are not merely secondary features. Specifically, myelin debris undermines the protective role of microglia, contributing to neuroinflammation and neuronal death. Herein, we examined central myelin and oligodendrocyte lineage progression in ASM knockout mice. We show that early-onset dysmyelination results from compromised oligodendrocyte maturation driven by aberrant sphingomyelin-mediated signaling. Transcriptomic profiling revealed that mature oligodendrocytes in these mice retain a gene expression signature similar to oligodendrocyte precursor cells, indicating a differentiation arrest. The cell adhesion molecule CD44 remained significantly upregulated in mature ASMko oligodendrocytes. Pharmacological inhibition of CD44 with verbascoside rescued oligodendroglial maturation in primary culture. Verbascoside administration in vivo restored myelin integrity and improved motor behavior. These findings establish that sphingomyelin homeostasis is critical for oligodendrocyte maturation and identify myelin defects as both primary pathological triggers and therapeutic targets for ASMD with neurologic symptoms.

## Synopsis

This study shows that sphingomyelin-induced impairment of oligodendrocyte (OL) differentiation and myelin alterations are early pathological events in acid sphingomyelinase deficiency (ASMD) and can be ameliorated by pharmacological inhibition of the extracellular matrix receptor CD44.

- Reduced myelin area, increased myelin debris, increased numbers of immature OLs, and reduced numbers of mature OLs are pathological hallmarks in the cerebellum of ASMko mice, a model of neurological ASMD.
- High sphingomyelin levels impair the morphological differentiation of cultured OLs.
- Transcriptomic analysis reveals an overlap between OL precursor cell and mature OL gene expression profiles in ASMko mice.
- CD44, which is physiologically downregulated to permit OL maturation, is aberrantly upregulated in mature OLs from ASMko mice.
- Inhibition of CD44 with Verbascoside in cultured ASMko OLs and in ASMko mice rescues OL maturation, ameliorates myelin abnormalities, and improves motor behavior.

## Abstract (web summary)

This study shows that sphingomyelin-induced impairment of oligodendrocyte (OL) differentiation and myelin alterations are early pathological events in acid sphingomyelinase deficiency (ASMD) and can be ameliorated by pharmacological inhibition of the extracellular matrix receptor CD44.

## Graphical abstract

**Figure:**
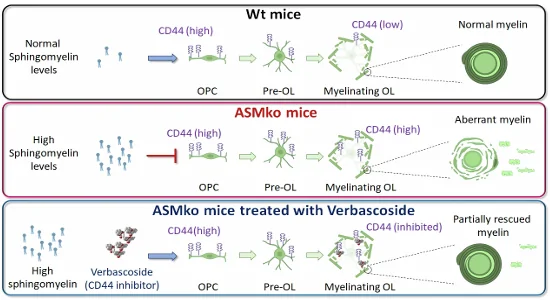

The paper explained **Problem**

Infantile neurovisceral acid sphingomyelinase deficiency (ASMD type A) is a fatal genetic neurodegenerative disorder. While myelin alterations were historically viewed as secondary to neuronal loss, emerging evidence identifies them as central drivers of ASMD pathogenesis. However, the cellular and molecular mechanisms underlying these defects remain poorly understood.

**Results**

We demonstrate that the maturation of myelin-producing oligodendrocytes is fundamentally impaired in the ASM-knockout (ASMko) mouse model. Mechanistically, we identified that the extracellular matrix receptor CD44, which typically undergoes programmed downregulation to permit oligodendrocyte maturation, remains pathologically elevated in the ASMD brain. Pharmacological inhibition of CD44 *via* oral administration of verbascoside restored oligodendrocyte maturation, attenuated myelin defects, and improved behavioral outcomes in ASMko mice.

**Impact**

These findings redefine the cellular landscape of ASMD brain pathology and establish CD44-targeted therapy as a promising strategy for this currently untreatable disease.

## Introduction

Mutations in the gene encoding acid sphingomyelinase (ASM), the lysosomal enzyme responsible for degrading sphingomyelin (SM), result in the storage disorder acid sphingomyelinase deficiency (ASMD) (Brady et al, 1966). In the ASMD type B clinical subset, genetic defects manifest as systemic pathology in peripheral organs while sparing the central nervous system. The recent approval of enzyme replacement therapy (ERT) *via* intravenous administration of recombinant ASM provides an effective treatment for these systemic manifestations (Wasserstein et al, 2018). However, as recombinant ASM cannot traverse the blood-brain barrier (BBB), ERT fails to slow down or stop the neurological progression. This is particularly critical for patients with ASMD type A, where genetic defects lead to early-onset, severe neurological symptoms, rapid neurodegeneration, and mortality within the first years of life, but also affects chronic neurovisceral ASMD patients (type A/B), where neurological symptoms are variable in severity and time of progression (Schuchman and Desnick, 2017).

The generation of the ASM knockout mouse model (ASMko) (Horinouchi et al, 1995) has been pivotal in elucidating ASMD brain pathology. Historically, research has prioritized characterizing neuronal alterations, documenting defects such as impaired calcium homeostasis (Pérez-Cañamás et al, 2017) and synaptic abnormalities (Arroyo et al, 2014; Bartoll et al, 2020). While neuroinflammation (microgliosis and astrocytosis) and dysmyelination are prominent in both ASMko mice (Samaranch et al, 2019; Ledesma et al, 2011; Gabandé-Rodríguez et al, 2019) and patients with neurologic ASMD (Landrieu and Saïd, 1984; Folkerth, 1999; Di Rocco et al, 2005), these features were traditionally dismissed as secondary pathological events. However, recent evidence has challenged this paradigm, establishing a causal role for microglia in ASMD-associated neurodegeneration (Gabandé-Rodríguez et al, 2019). These studies reveal that ASMko microglia attempt to clear myelin debris–a byproduct of dysmyelination–which, when added to the genetic defect, causes excessive lysosomal SM accumulation. This overload triggers lysosomal membrane permeabilization and subsequent exocytosis, resulting in the extracellular release of cathepsin B. As a potent lysosomal hydrolase, cathepsin B exerts direct neurotoxicity, thereby linking myelin defects to active neuronal death (Gabandé-Rodríguez et al, 2019).

Although recent findings identify dysmyelination as a primary trigger for ASMD brain pathology, the specific cellular mechanisms governing central myelin defects and oligodendrocyte (OL) dysfunction remain poorly understood. Herein, we utilized the ASMko mouse model to establish the temporal and structural progression of CNS myelin alterations. Furthermore, we characterized the lineage-specific defects in ASMko oligodendroglia and evaluated both in vitro and in vivo pharmacological strategies to restore OL maturation and myelin integrity.

## Results

### Myelin alterations are an early event in the cerebellum of ASMko mice

**Figure 1:**
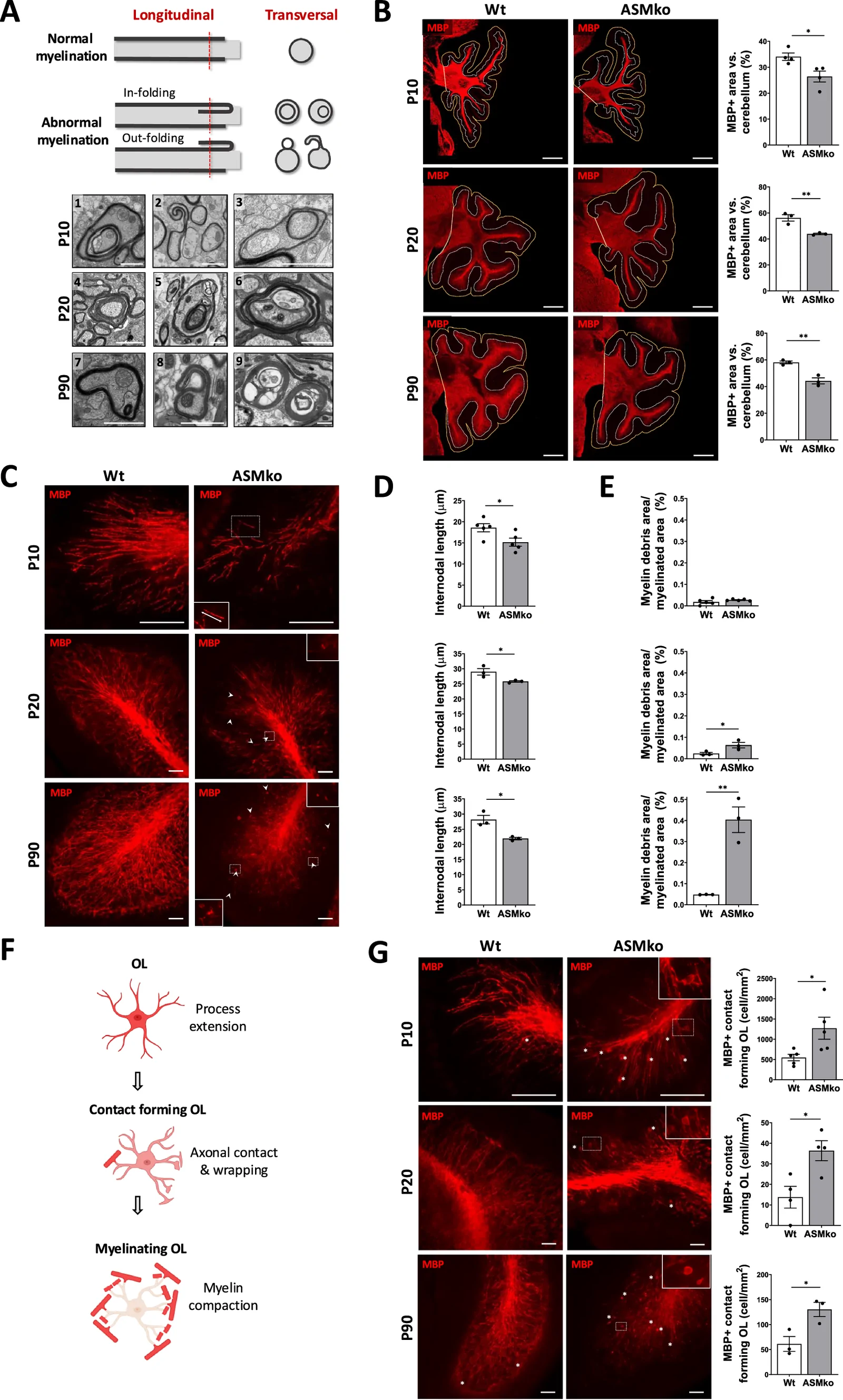
Structural myelin abnormalities are an early pathological event in the ASMko mouse cerebellum. (**A**) Schematic representation (top) of physiological myelin organization and common structural abnormalities (in-folding and out-folding) in longitudinal and transversal views. Representative electron microscopy images (bottom) show transverse myelinated axons in the cerebellum of ASMko mice at P10 (1–3), P20 (4–6), and P90 (7–9). Images 1, 3, 4, 5, 6, and 7 correspond to examples of in-folding, images 2 and 8 to out-folding, and image 9 shows in-folding accompanied by overt axonal degeneration. Scale bar: 1 μm. (**B**) Representative low-magnification immunofluorescence images of the myelin marker MBP in sections of whole cerebellum from WT and ASMko mice at P10, P20, and P90. The total cerebellar area is outlined by a continuous yellow line, while the MBP^+^ area is delimited by a white dashed line. Data show mean ± SEM percentage of the cerebellar area occupied by MBP. Statistical analysis was performed using two-tailed *t* test (P10: *n* = 4, *P* = 0.024; P20: *n* = 3, *P* = 0.009; P90: *n* = 3, *P* = 0.005). Scale bar: 500 μm. (**C**) Representative high-magnification immunofluorescence images of MBP within the apical regions of the cerebellar lobules of WT and ASMko mice at P10, P20, and P90. At P10, images represent 15-μm z-stack projections (acquired at ×40) to ensure accurate visualization of nascent, fine-diameter myelinated fibers. Images for P20 and P90 were acquired at ×25. A representative internode is shown at higher magnification in the ASMko panel at P10, where internodal length is indicated by a double-headed arrow in the inset. Myelin debris is indicated by arrowheads, with magnified views shown in insets of ASMko images at P20 and P90. Scale bar: 50 μm. (**D**, **E**) Data show mean ± SEM internodal length in μm (**D**; P10: *n* = 5, *P* = 0.035; P20: *n* = 3, *P* = 0.045; P90: *n* = 3, *P* = 0.013) and area occupied by myelin debris as a percentage of the total myelinated area (**E**; P10: *n* = 5, *P* = 0.200; P20: *n* = 3, *P* = 0.049; P90: *n* = 3, *P* = 0.004). Statistical analysis was performed using two-tailed *t* test. (**F**) Schematic illustrating the sequential stages of MBP distribution (red) during myelin formation: (i) Process extension: mature OLs exhibit uniform (symmetric) MBP distribution throughout the cell body and processes. (ii) Axonal contact and initial wrapping: in contact-forming OL, MBP undergoes asymmetric redistribution, progressively clearing from the cell body and trafficking into the extending processes as myelin ensheathment initiates. (iii) Myelin compaction: MBP localization is highly polarized, predominantly restricted to compact myelin sheaths, marking fully myelinating OLs. (**G**) Representative immunofluorescence images of MBP in the cerebellar lobules of WT and ASMko mice at P10 (×40), P20 (×25), and P90 (×25). Asterisks mark MBP^+^ contact-forming OLs; the upper-right insets show magnified views. Data show mean ± SEM number of MBP^+^ cells at the stage of axonal contact and initial wrapping per area of the cerebellar lobules. Statistical analysis was performed using two-tailed *t* test (P10: *n* = 5, *P* = 0.034; P20: *n* = 4, *P* = 0.020; P90: *n* = 3, *P* = 0.029). Scale bar: 50 μm. Asterisks in the graphs of all panels denote significance levels: *P  <  0.05; **P  <  0.01. Source data are available online for this figure.

**Figure EV1:**
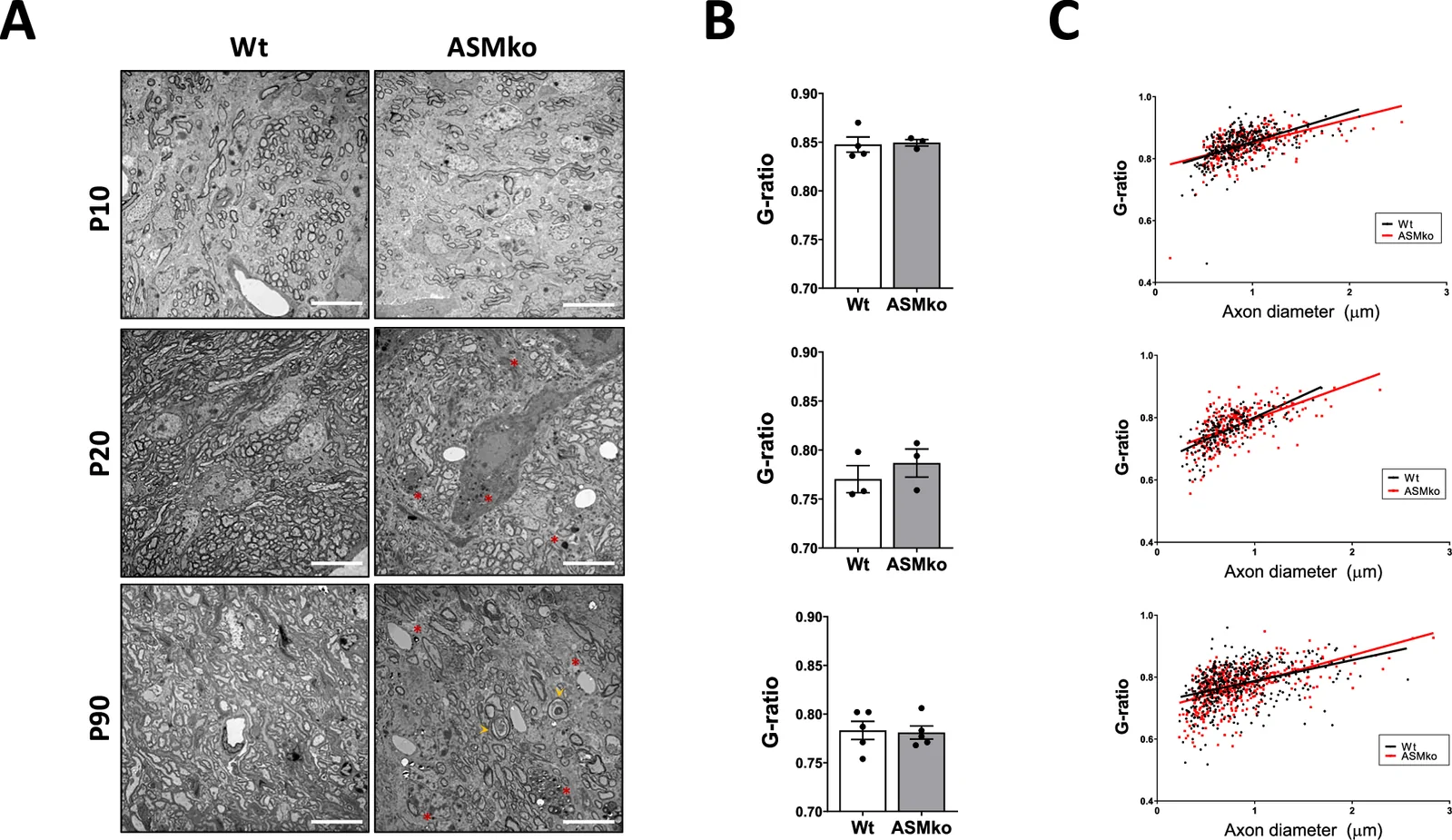
Myelin characterization in the cerebellum of WT and ASMko mice. (**A**) Electron micrographs of white matter from cerebellar lobules of WT and ASMko mice at P10, P20, and P90. Red asterisks indicate intracellular accumulation of electron-dense lipid droplets in ASMko samples; by P90, signs of fiber degeneration and axonal loss become evident (yellow arrowheads). Only transverse sections of myelinated fibers with intact axonal morphology and normally compacted myelin were included for g-ratio measurements. Scale bar: 10 µm. (**B**) Graphs show mean ± SEM g-ratio of myelin thickness of wt and ASMko mice at P10, P20, and P90; P10: Statistical analysis was performed using two-tailed *t* test. *n* = 4 wt, 3 ASMko, *P* = 0.857; P20: *n* = 3 per genotype, *P* = 0.459; P90: *n* = 5 per genotype, *P* = 0.853). (**C**) Scatter plot showing the relationship between g-ratio and axon diameter (< 3 µm) in wt and ASMko mice. Linear regression analyses reveal no significant differences between genotypes (P10: *P* = 0.144; P20: *P* = 0.054; P90: *P* = 0.059). More than 150 fibers were analyzed from at least three mice per genotype. Source data are available online for this figure.

While myelin defects in 3-month-old ASMko mice have been documented in the cerebellum (Gabandé-Rodríguez et al, 2019), the region most susceptible to microgliosis and neurodegeneration in this model (Gabandé-Rodríguez et al, 2019; Sarna et al, 2001), it remains unclear whether these defects emerge as a primary pathological driver or a secondary consequence of disease progression. To resolve this, we employed electron microscopy to characterize myelin alterations at the following key developmental stages: postnatal day 10 (P10) (active myelination (Quarles et al, 2006)), P20 (peak myelination rate (Baumann and Pham-Dinh, 2001; Ozarkar et al, 2025)), and P90 (established dysmyelination in ASMko mice (Gabandé-Rodríguez et al, 2019)). Ultrastructural analysis revealed aberrant myelination features, including in- and out-foldings, as early as P10 in ASMko mice. These structural irregularities progressively worsened with age, ultimately accompanied by signs of overt axonal degeneration (Fig. 1A). Complementary immunofluorescence staining of myelin basic protein (MBP) confirmed an early-onset deficit, with a significant 17% reduction in the MBP^+^ area evident at P10 in ASMko mice compared with WT mice, increasing to 22% at P20 and 24% at P90 (Fig. 1B). Despite the presence of the malformed profiles and reduced overall myelin density in the cerebellum of ASMko mice, the thickness of morphologically preserved, compact myelin sheaths remained unaffected, as indicated by comparable G-ratio values across all development stages (Fig. EV1). However, as this analysis was restricted to fibers lacking overt abnormalities, it does not exclude the possibility of local alterations in myelin compaction at sites displaying in- and out-folding.

MBP staining revealed two additional features of myelin pathology in ASMko mice: reduced internodal length and the accumulation of myelin debris (Fig. 1C). Internodal length was shortened by 18%, 11%, and 22% at P10, P20, and P90, respectively, when compared with WT mice (Fig. 1D). The partial normalization at P20 could reflect a transient delay in internode maturation; however, the subsequent shortening at P90 is consistent with the concurrent effects of myelin degeneration and the generation of newly formed, shorter internodes during abortive remyelination. Furthermore, significant myelin debris was evident at P20 (2.6-fold increase) and further increased at P90 (8.4-fold increase) relative to WT (Fig. 1E). MBP staining also identified an increase in the number of contact-forming OLs at the initial wrapping stage, which precedes myelin compaction (Bauer et al, 2009) (Fig. 1F), at P10 (93%), P20 (165%), and P90 (113%) (Fig. 1G). The accumulation of these cells suggests a stalled progression through the myelination program early in development, while at later stages it likely reflects the enhanced recruitment of newly differentiating OLs in a compensatory response to chronic myelin loss.

To explore whether the myelin alterations identified in ASMko mice are also present in humans, we analyzed cerebellar tissue from a patient with ASMD type A and an age-matched control child (Appendix Fig. S1). Although these observations should be interpreted with caution, as they are based on a single case due to the limited availability of fixed brain tissue from this ultra-rare disease, MBP staining revealed alterations consistent with those observed in ASMko mice. These include a 34% reduction in MBP^+^ area, a 12-fold increase in myelin debris, and a 2.2-fold increase in the number of MBP^+^ contact-forming OLs, while the total area occupied by oligodendroglia cells (identified by SOX10 staining) was not significantly affected (Appendix, Fig S1).

### ASMko OLs exhibit cell-autonomous, SM-dependent maturation defects

**Figure 2:**
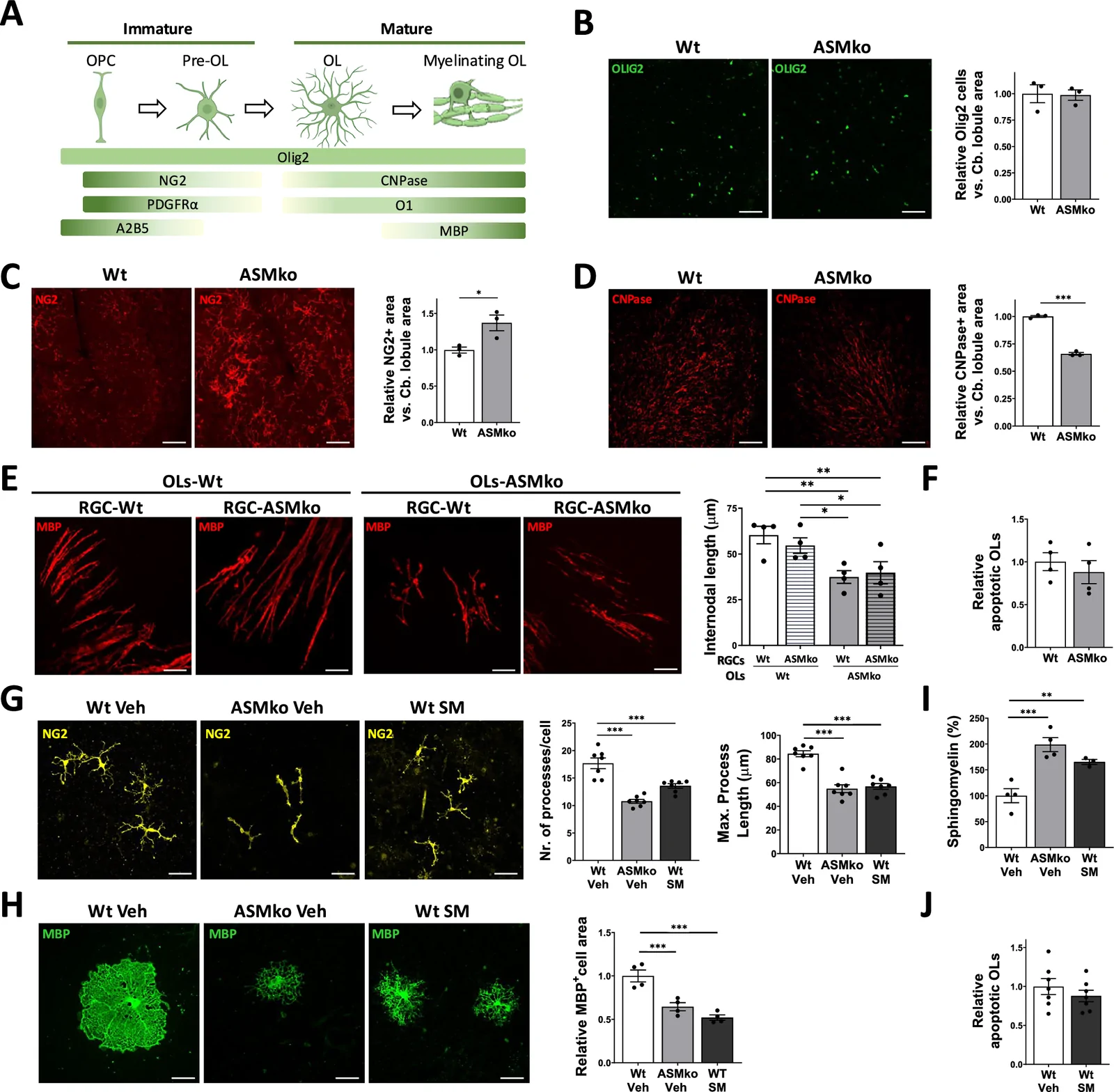
Impaired differentiation of ASMko OL. (**A**) Schematic representation of the OL lineage, illustrating the progressive morphological transition from bipolar OPCs through pre- myelination OLs to highly branched, mature, and myelinating OLs, together with the stage-specific markers used in this study (Olig2, NG2, PDGFRα, A2B5, CNPase, O1, MBP). These markers identify successive maturation states, with Olig2 labeling the entire lineage. For each experiment, markers were selected based on their technical suitability for the specific developmental window being interrogated. (**B**–**D**) Representative immunofluorescence images of Olig2 (**B**), NG2 (**C**), and CNPase (**D**) in the apical region of cerebellar lobules from WT and ASMko mice at P90. Data show the mean ± SEM number of Olig2^+^ cells (**B**), or mean ± SEM area occupied by NG2 (**C**) or CNPase (**D**) relative to the total lobular area analyzed. Statistical analyses were performed using two-tailed *t* test (Olig2: *n* = 3, *p*_Olig2_ = 0.899; NG2: *n* = 3, *p*_NG2_ = 0.032; CNPase: *n* = 3, *p*_CNPase_ < 0.0001). Scale bar: 50 μm. (**E**) Representative immunofluorescence images of MBP in the retina of WT and ASMko mice after transplantation of WT or ASMko OLs onto RGCs, as indicated. Data show mean ± SEM length (µm) of individual myelin internodes along RGC axons 5 weeks after transplantation (*n* = 4 mice per group). Statistical comparisons relative to control (RGC-WT/OPC-WT): RGC-ASMko/OPC-WT, *P* = 0.405; RGC-WT/OPC-ASMko, *P* = 0.005; RGC-ASMko/OPC-ASMko, *P* = 0.009. Additional pairwise comparisons: RGC-ASMko/OPC-WT vs. RGC-WT/OPC-ASMko, *P* = 0.024; RGC-ASMko/OPC-WT vs. RGC-ASMko/OPC-ASMko, *P* = 0.046; and RGC-WT/OPC-ASMko vs. RGC-ASMko/OPC-ASMko, *P* = 0.732). Statistical analysis was performed using one-way ANOVA followed by Sidak’s post hoc test. Scale bar: 50 μm. (**F**) Data show mean ± SEM number of apoptotic cells (TUNEL^+^) in cultured WT or ASMko OLs as a percentage of the total number of Olig2^+^ cells. Statistical analyses were performed using two-tailed *t* test (*n* = 4, *P* = 0.500). (**G**) Representative immunofluorescence images of NG2 in 4-day differentiated OL cultures from WT or ASMko OLs treated with vehicle, and in WT OLs treated with SM. Data show mean ± SEM number of processes and length of processes in NG2^+^ cells. Statistical analyses were performed using one-way ANOVA followed by Sidak’s post hoc test (*n* = 7; *p*_number ASMko_ < 0.001, *p*_number Wt SM_ < 0.001, *p*_length ASMko_ < 0.001, *p*_length Wt SM_ < 0.001). Scale bar: 50 μm. (**H**) Representative immunofluorescence images of MBP in 4-day differentiated OL cultures from WT or ASMko OLs treated with vehicle, or in WT OLs treated with SM. Data show the mean ± SEM area occupied by MBP. Statistical analysis was performed using one-way ANOVA followed by Sidak’s post hoc test (*n* = 4; *p*_MBP area ASMko_ < 0.001, *p*_MBP area Wt SM_ < 0.001). Scale bar: 50 μm. (**I**) Data show the mean ± SEM levels of SM in cultured WT or ASMko OLs treated with vehicle, and in WT OLs treated with SM, expressed as a percentage relative to vehicle-treated WT OLs. Statistical analysis was performed using one-way ANOVA followed by Sidak’s post hoc test (*n* = 4 for wt and ASMko; *n* = 3 wt+SM; *p*_ASMko_ < 0.001, *p*_Wt+SM_ = 0.007). (**J**) Data show the mean ± SEM percentage of apoptotic cells (TUNEL^+^) relative to the total number of Olig2⁺ cells in cultured WT OLs treated with vehicle or SM. Statistical analysis was performed using two-tailed *t* test (*n* = 7, *P* = 0.359). Asterisks in the graphs denote significance levels: *P  <  0.05. Source data are available online for this figure.

The myelin abnormalities observed in the ASMko cerebellum suggested a primary deficit in OL biology, prompting a detailed investigation of the lineage at P90, a stage of established OL differentiation (Baumann and Pham-Dinh, 2001). We evaluated the OL lineage in vivo using the following stage-specific markers: Olig2, which labels the entire oligodendroglial lineage; NG2, which marks OPCs and early differentiating cells (pre-OLs); and CNPase, a marker of mature OLs (Fig. 2A) (Traiffort et al, 2016). While the number of oligodendroglial lineage cells (Olig2^+^) remained comparable between ASMko and WT mice (Fig. 2B), the area occupied by NG2^+^ cells increased by 37% (Fig. 2C). Conversely, the CNPase^+^ mature OL area was decreased by 34% (Fig. 2D). These results suggest that ASM deficiency impairs OL maturation.

We next utilized a retinal myelination model to determine if this impairment is intrinsic to the OLs. Taking advantage of the fact that the retina lacks endogenous OLs, we delivered exogenous OPCs to induce axonal wrapping and myelination (Setzu et al, 2004; Nocera et al, 2024). We isolated A2B5^+^ OPCs (Fig. 2A) from both ASMko and WT brains and injected them into the vitreous humor, adjacent to the retinal ganglion cell axons, of 2-month-old WT and ASMko recipient mice. This cross-transplantation approach allowed us to discern whether the myelination defect is a cell-autonomous trait or driven by the ASM-deficient axon environment. Five weeks post-injection, we analyzed the internodal length of the newly formed myelin by immunostaining against MBP (internodes) and Caspr (paranodes) to ensure analysis of complete internodal segments (Appendix, Fig. S2) (Nocera et al, 2024; Murcia-Belmonte et al, 2016). While WT OPCs produced internodes of normal length in both WT and ASMko retinas, ASMko OPCs displayed a marked reduction in internodal length when injected into WT (38% reduction) or ASMko (34% reduction) environments (Fig. 2E), reproducing the defect shown in the cerebellum. These findings show that the reduced internodal length is determined by the genotype of the oligodendroglial cell rather than by the axonal environment, indicating a cell-autonomous defect in ASMko myelin formation.

The cell-autonomous maturation defects observed in vivo prompted us to investigate OL differentiation in primary OPC cultures. To ensure population homogeneity, A2B5⁺ OPCs were purified by MACS from P4 mouse brains, establishing a synchronized baseline at the early progenitor stage. Following purification, OPCs were plated on poly-L-lysine/laminin and differentiated for 4 days. Consistent with the stable Olig2^+^ numbers observed in WT and ASMko brain tissue (Fig. 2B), ASMko cultures showed no increase in apoptosis compared with WT, as confirmed by TUNEL assay (Fig. 2F). However, reflecting the lineage imbalance seen in the ASMko brain (Fig. 2C), ASMko OPCs exhibited a marked impairment in morphological maturation after 4 days of in vitro differentiation. Specifically, ASMko OPCs showed a reduction in both the number (0.61-fold) and length (0.65-fold) of their cellular processes relative to WT controls (Fig. 2G). Furthermore, the MBP⁺ area in mature OLs was 0.65-fold smaller than in WT cultures (Fig. 2H).

We next investigated the underlying cause of impaired ASMko OL maturation. Given that SM accumulation is a pathological hallmark of ASM deficiency, we supplemented the differentiation medium of WT cells with this lipid for 4 days. To mimic the progressive accumulation during differentiation, cultures were treated with 20 µM SM on day 1 and 40 µM on day 3 maintained until day 4, resulting in a 65% increase in cellular SM levels comparable to ASMko OLs (Fig. 2I), without inducing apoptosis (Fig. 2J). Remarkably, SM-treated WT oligodendroglia effectively phenocopied the ASMko phenotype, exhibiting a reduction in process number and length (0.77 and 0.67-fold, respectively) in NG2⁺ progenitors (Fig. 2G) and a decrease in MBP⁺ area (0.52-fold) in mature OLs (Fig. 2H).

### Transcriptomic profiling of ASMko OPCs and OLs confirms maturation arrest and suggests altered extracellular matrix interactions

**Figure 3:**
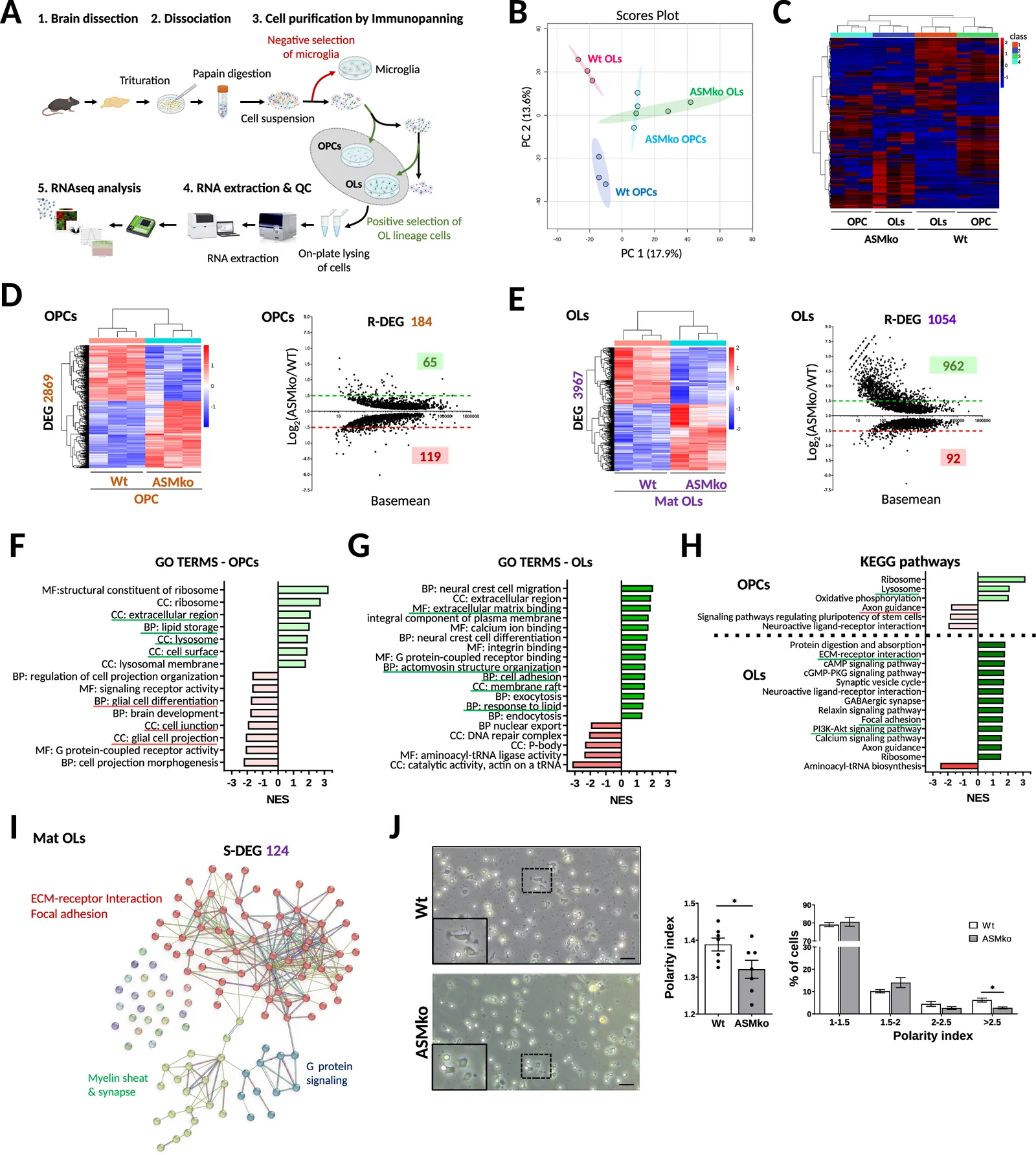
Altered transcriptional signatures in ASMko OPCs and OLs reflect a profound maturation arrest and dysregulated ECM interactions. (**A**) Graphical illustration of the workflow used to isolate OPCs and mature OLs from mice for RNA-seq analysis. Brains were dissected and enzymatically dissociated, and single-cell suspensions were sequentially immunopanned to deplete non-OL lineage cells and to enrich for PDGFRα⁺ OPCs or O1⁺ mature OLs. Purified cells were used for RNA extraction, library preparation, and high-throughput sequencing. (**B**) PCA of RNA-seq profiles from purified WT and ASMko OPCs and OLs (three biological replicates per group). Each dot represents an individual sample. (**C**) Hierarchical clustering heatmap of the 1000 most variable transcripts (ANOVA). Rows correspond to transcripts, columns to samples, and the color scale indicates normalized expression values (z-score). (**D**, **E**) Analysis of differentially expressed genes in ASMko vs. WT OPCs (**D**) and mature OLs (**E**). Left: heatmaps showing the expression patterns of the significantly deregulated genes (DEG; *q* value < 0.05) between genotypes. Rows represent individual DEG and columns represent biological replicates; color intensity reflects relative expression levels after row-wise scaling. Right: volcano plots of the same DEG, with robust deregulated genes (R-DEG) defined by a |log₂ FC > 1.5 (dashed lines). (**F**, **G**) Gene Ontology (GO) enrichment analysis of DEG in ASMko vs. WT OPCs (**F**) and mature OLs (**G**). Horizontal bar plots display the normalized enrichment score (NES) for each term. Because numerous GO categories reached statistical significance (adjusted *P* < 0.05), panels show a curated subset of biologically relevant terms to facilitate interpretation. GO categories are indicated by their prefixes: Biological Process (BP), Molecular Function (MF), and Cellular Component (CC). (**H**) KEGG pathway enrichment analyses of differentially expressed genes in ASMko vs. WT OPCs and mature OLs, shown as a single composite panel. The upper section displays pathways significantly enriched in OPCs (adjusted *P* < 0.05), whereas the lower section, separated by a dotted line, shows those enriched in mature OLs. Horizontal bar plots indicate NES values. As with GO analysis, only pathways with clear biological relevance to the study were included to enhance interpretability. (**I**) STRING network representation of the most strongly dysregulated genes (S-DEG) in mature OLs comparing ASMko and WT ( | log₂FC | > 3). Nodes represent individual genes and edges indicate predicted functional associations. Network clustering reveals three major functional modules. (**J**) Representative phase-contrast images of WT and ASMko OPC cultures 2 h after plating on laminin-coated coverslips; insets show magnified representative cells for each genotype. Data show the mean ± SEM polarity index (left), and the mean ± SEM percentage of cells reaching the indicated polarity index (right). Statistical analyses were performed using two-tailed *t* test (*n* = 7; >50 cells analyzed per sample; *p*_PI_ = 0.046 for mean polarity index; distribution *P* values: *P*_1–1.5_ = 0.578, *P*_1.5–2_ = 0.346, *P*_2–2.5_ = 0.346; *P *> _2.5_ = 0.016). Scale bar: 50 µm. Asterisks in the graphs of all panels denote significance levels: *P  <  0.05. Source data are available online for this figure.

To define the molecular landscape underlying impaired ASMko OL maturation, we isolated PDGFRα^+^ OPCs and O1^+^ OLs (Fig. 2A) from WT and ASMko brains by immunolabeling followed by immunopanning. This purification method was selected for its ability to isolate viable cell populations with minimal transcriptional perturbation for RNA-sequencing analysis (Fig. 3A), constituting the gold standard immunopanning strategy for OPC and mature OL purification (Emery and Dugas, 2013; Barres, 2014). The success of the OL isolation protocol was confirmed by the high enrichment of OL-specific genes and the minimal expression of markers associated with astrocytes, microglia, and neurons (Appendix Fig. S3). Comparison of overall transcriptional profiles by principal component analysis (PCA) revealed a clear separation by genotype along the first principal component (PC1), and by developmental stage (OPCs vs. OLs) along the second (PC2) in WT samples. By contrast, ASMko samples exhibited substantial transcriptional overlap between developmental stages, consistent with a differentiation arrest from OPCs to mature OLs (Fig. 3B).

In agreement with the PCA results, hierarchical clustering of the top 1000 most variable transcripts primarily segregated samples by genotype. Within each genotype, samples clustered by developmental stage, further reinforcing the evidence of disrupted maturation in ASMko cells (Fig. 3C). Focusing on genotype-dependent transcriptional differences within each stage (Fig. 3D,E), we identified 2869 and 3967 significantly differentially expressed genes (DEGs) in OPCs (Fig. 3D) and mature OLs (Fig. 3E), respectively. When we applied a more stringent cutoff ( | log₂FC | > 1.5) to determine the robust deregulated genes (R-DEG), a striking contrast emerged. While OPCs displayed only 184 R-DEGs (65 upregulated, 119 downregulated), mature OLs exhibited a fivefold increase, with 1054 R-DEGs (962 upregulated, 92 downregulated). This strong increase in transcriptional divergence of the genotypes, predominantly driven by gene upregulation, emerging at the mature OL stage, indicates that molecular defects are progressively amplified as differentiation proceeds, culminating in a profound loss of the mature OL identity.

To investigate the functional implications of the transcriptional changes observed in OPCs and mature OLs, we performed Gene Set Enrichment Analysis (GSEA), which identifies coordinated, subtle shifts in predefined gene sets by integrating the magnitude and direction of expression changes across all genes. We evaluated enrichment across Gene Ontology (GO) categories (biological processes, molecular functions, and cellular components) as well as Kyoto Encyclopedia of Genes and Genomes (KEGG) pathways.

In ASMko OPCs, GSEA revealed significant enrichment of genes associated with glial cell differentiation and projection, lipid storage, sphingolipid metabolism, lysosomal function, and ribosomal activity (Fig. 3F). Positively enriched GO terms included ribosome (normalized enrichment score [NES] = 3.271), lysosome (NES = 1.932), and lipid storage (NES = 2.044), indicating that these gene sets are overrepresented among the most highly expressed genes. These processes reflect protein processing, catabolism, and lipid metabolism, consistent with the lysosomal lipid storage phenotype of ASMD. By contrast, gene sets linked to glial cell differentiation (NES = −1.764) and glial cell projection (NES = −2.095) were negatively enriched, meaning that these genes are downregulated in ASMko OPCs compared with controls, consistent with impaired maturation. KEGG pathway analysis reinforced these findings, showing enrichment of ribosome (NES = 3.157), lysosome (NES = 2.114), and oxidative phosphorylation (NES = 2.048), as well as negative enrichment of axon guidance (NES = −1.788) and stem cell pluripotency pathways (NES = −1.866; Fig. 3H). Together, the GO and KEGG results indicate a broad shift toward increased metabolic and catabolic activity accompanied by a suppression of differentiation-related programs in OPCs.

In mature OLs, enriched gene sets converged on pathways involved in lipid response, membrane trafficking, membrane rafts, extracellular matrix (ECM) binding, calcium ion binding, and integrin-mediated interactions (Fig. 3G), which are processes essential for myelin formation and axonal support. ECM binding showed robust positive enrichment (NES = 1.894), highlighting structural alterations in OL-ECM interactions. This ECM-related signature included coordinated changes in both extracellular matrix components and cell-surface receptors, suggesting a broad remodeling of OL-matrix interactions at the mature stage. Negatively enriched terms, including regulation of glial differentiation (NES = −1.696) and axon ensheathment (NES = −1.202), point to a failure in maturation and myelination. These findings were echoed in KEGG analysis, with enrichment of ECM-receptor interactions, focal adhesion, PI3K-Akt, cAMP/cGMP-PKG, and calcium signaling, and axon guidance (Fig. 3H), indicating dysregulation of signaling and structural pathways crucial for OL differentiation and myelination.

Taken together, these findings indicate stage-specific transcriptional disruptions in ASMko OPCs and mature OLs, linking altered metabolic, lipid-handling, and ECM-related pathways to impaired differentiation and myelin formation. To resolve the molecular drivers of these defects, we prioritized the most highly dysregulated genes in mature OLs ( | log₂FC | > 3). Network-based clustering of these 124 candidates using the STRING database revealed three distinct functional modules (Fig. 3I). A dominant cluster comprised genes associated with ECM-receptor interactions and focal adhesion, reinforcing the pathway-level shifts identified by GSEA. The convergence of these highly dysregulated nodes on adhesion- and signaling-related pathways points to a coordinated breakdown of the structural and communication programs critical for OL maturation. Given the prominence of these ECM-related perturbations, we next assessed whether these transcriptional alterations translated into functional defects in cell adhesion and spreading in vitro (Yamada et al, 2022). MACS-purified OPCs were cultured on poly-L-lysine/laminin-coated coverslips and imaged after 2 h to evaluate early spreading and polarization. ASMko OPCs exhibited a significantly reduced polarity index compared with WT cells (Fig. 3J), primarily driven by a 57% reduction in the proportion of highly polarized cells (from 6.2% in WT to 2.6% cells in ASMko, with polarity index >2.5). These findings indicate that ASM deficiency impairs the initial stages of cell spreading and polarization, providing functional evidence of altered interactions with the extracellular environment.

### CD44 is overexpressed in ASMko OLs, and its pharmacological inhibition rescues OL maturation and ameliorates brain pathology

**Figure 4:**
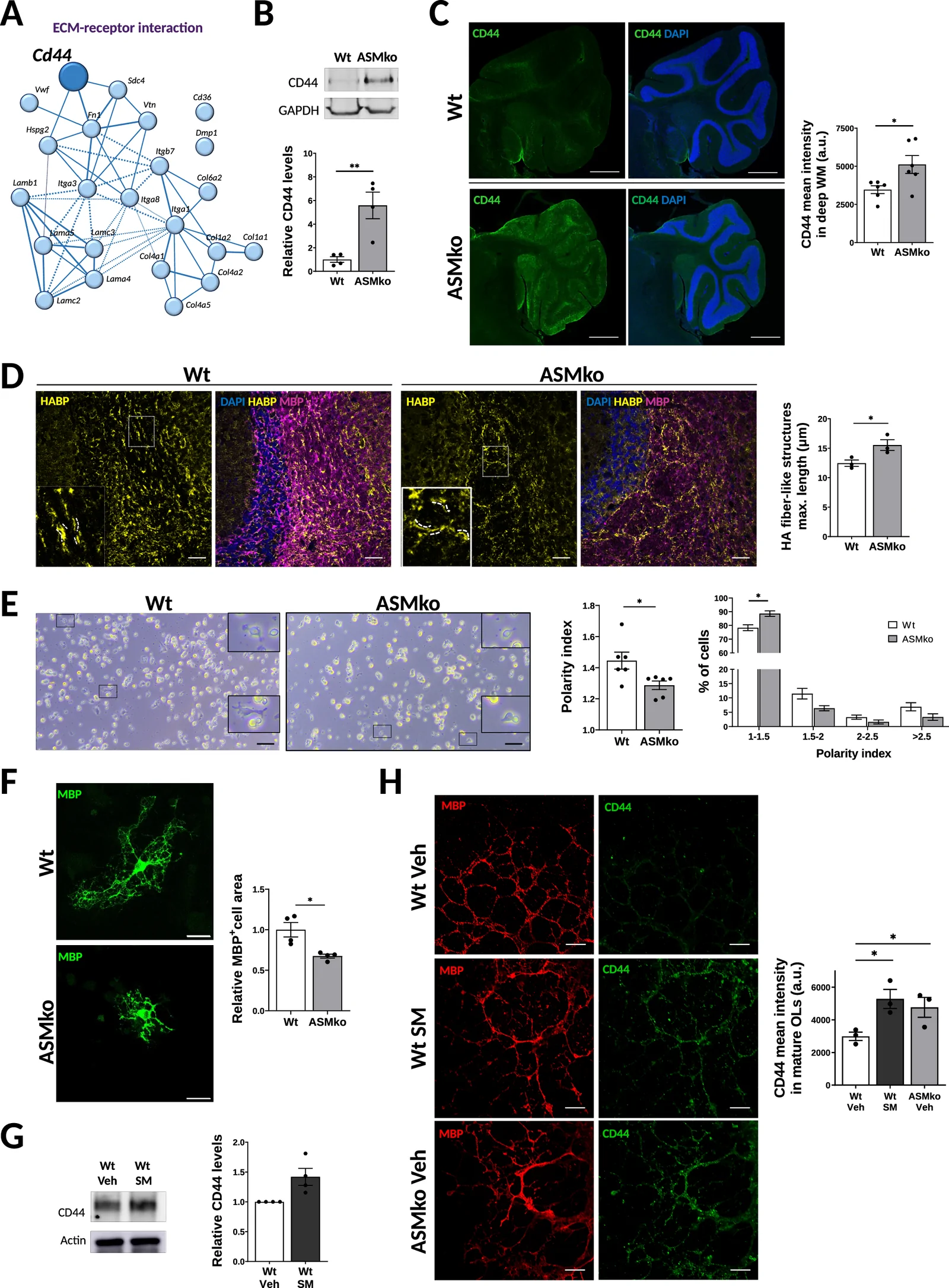
Increased CD44 protein expression in ASMko OLs is due to high SM. (**A**) Increased *Cd44* expression from the 23 DEGs related to the ECM-receptor interaction pathway in ASMko OLs compared with mature OLs. Blue nodes denote upregulated genes. (**B**) Representative western blot of CD44 protein levels in cerebellar extracts from P90 WT and ASMko mice. GAPDH was used as a loading control. Data show mean ± SEM CD44 levels in arbitrary units normalized to GAPDH. Statistical analysis was performed using two-tailed *t* test (*n* = 4; *P* = 0.007). (**C**) Representative immunofluorescence images of CD44 in the cerebellar deep white matter of P90 WT and ASMko mice. Data show mean ± SEM CD44-associated intensity in arbitrary units. Statistical analysis was performed using two-tailed *t* test (*n* = 6; *P* = 0.027). DAPI staining in blue shows cell nuclei. Scale bar: 500 µm. (**D**) Representative immunofluorescence images of HA patterns (labeled by immunostaining against HABP) in the cerebellar deep white matter of P90 WT and ASMko mice. Insets show magnified representative organization patterns for each genotype. White dashed lines highlight the fiber-like structures. Data show mean ± SEM length of the five longest HABP⁺ fiber-like structures per image. Statistical analysis was performed using two-tailed *t* test (*n* = 3; *P* = 0.044). Scale bar: 50 µm. (**E**) Representative phase-contrast images of 4-day differentiated OL cultures from WT and ASMko mice cultured in hyaluronic acid-coated coverslips; insets show magnified representative cells for each genotype. Data show the mean ± SEM polarity index (left), and the mean ± SEM percentage of cells reaching the indicated polarity index (right). Statistical analysis was performed using two-tailed *t* test. (*n* = 6; *p*_PI_ = 0.027 for mean polarity index; distribution *P* values: *P*_1–1.5_ = 0.021, *P*_1.5–2_ = 0.096, *P*_2–2.5_ = 0149; *P*_>2.5_ = 0.149). Scale bar: 50 µm. (**F**) Representative immunofluorescence images of MBP in 4-day differentiated OL cultures from WT or ASMko OLs cultured in hyaluronic acid-coated coverslips. Data show the mean ± SEM relative area occupied by MBP. Statistical analysis was performed using two-tailed *t* test (*n* = 4; *P* = 0.012. Scale bar: 50 μm. (**G**) Representative western blot of CD44 protein levels in cultured OLs differentiated for 4 days from WT mice incubated with vehicle or SM. Actin was used as a loading control. Data show mean ± SEM CD44 levels in arbitrary units normalized to Actin. Statistical analysis was performed using a one-sample *t* test (*n* = 4; *P* = 0.060). (**H**) Representative immunofluorescence images of MBP and CD44 in cultured OLs differentiated for 4 days from WT and ASMko mice treated with vehicle, or from WT OLs treated with SM. Images show magnifications of processes from mature MBP^+^ OLs. Data show mean ± SEM CD44 intensity in MBP^+^ OL processes in arbitrary units. Statistical analysis was performed using one-way ANOVA followed by Sidak’s post hoc test (*n* = 3; *p*_ASMko vs. Wt_ = 0.049, *p*_Wt+SM vs. Wt_ = 0.019, *p*_Wt+SM vs. ASMko_ = 0.501). Scale bar: 5 µm. Asterisks in the graphs of all panels denote significance levels: *P  <  0.05; **P  <  0.01. Source data are available online for this figure.

Among the 23 dysregulated genes associated with extracellular receptor interaction in mature OLs (Fig. 4A), *Cd44* emerged as a prominent candidate, with transcript levels increased by 5.5-fold relative to WT. CD44 is a transmembrane glycoprotein and the main surface receptor for hyaluronan (HA) (Dzwonek and Wilczynski, 2015). While CD44 expression is typically downregulated during OL maturation, its sustained expression has been shown to inhibit morphological differentiation (Naruse et al, 2013). Furthermore, mice overexpressing CD44 exhibit differentiation defects and a distinct dysmyelination profile (Tuohy et al, 2004; Back et al, 2005; Sato et al, 2022). These observations suggest that the aberrant upregulation of CD44 in ASMko mature OLs may be a primary driver of the impaired differentiation program. To test this hypothesis, we first validated whether the increased *Cd44* levels observed in the RNA-seq analysis translated to the protein level. Western blot analysis of cerebellar extracts of P90 ASMko and WT mice confirmed a 5.6-fold increase in CD44 protein levels in ASMko mice (Fig. 4B). Immunofluorescence further revealed a 48% increase in CD44 signal intensity within the deep white matter of the ASMko cerebellum, a region highly enriched for myelinating OLs (Fig. 4C). While staining for HA showed no change in overall abundance, the spatial organization was altered, characterized by a 25% increase in fiber-like structures (Baier et al, 2007) in ASMko mice compared with WT controls (Fig. 4D). The aberrant increase of CD44 in the mature OLs prompted us to analyze in vitro their functional response to its physiological ECM ligand, HA. To this aim, 4-day differentiated cultures derived from MACS-purified OPCs (a stage enriched in differentiating oligodendroglial cells) were replated onto HA-coated coverslips. Cells were imaged after 7 h to evaluate early spreading and polarization. ASMko OLs exhibited a significant 11% reduction in polarity index compared with WT cells (Fig. 4E), primarily driven by a 13% increase in the proportion of poorly polarized cells (from 78.32% in WT to 88.63% in ASMko cells, with polarity index <1.5). To determine whether these early defects translated into altered morphological progression during continued differentiation, cells were maintained on the same HA-coated substrates for an additional 4 days after replating and subsequently analyzed by MBP immunofluorescence. ASMko MBP^+^ cells displayed a 34% reduction in cell area compared with WT cells (Fig. 4F), consistent with impaired morphological development on HA substrates. Together, these findings suggest that ASM deficiency alters CD44-dependent responses to HA in differentiating oligodendroglial cells. As a control, we also analyzed the early spreading and polarization of freshly MACS-isolated OPCs plated onto HA-coated coverslips. In contrast to cells derived from 4-day differentiated cultures, no differences were detected between WT and ASMko OPCs (Appendix Fig. S4A,B), consistent with the absence of CD44 dysregulation at this early developmental stage.

Since exogenous SM supplementation impairs OL maturation (Fig. 2I,J), we investigated whether elevated SM levels directly drive the upregulation of CD44 expression. Primary WT OLs were differentiated in the presence of increasing concentrations of SM (20 µM on day 1 and 40 µM on day 3) over 4 days. Western blot analysis of whole-cell extracts revealed a 1.4-fold increase in CD44 protein levels in SM-treated cultures relative to controls (Fig. 4G). Given that CD44 contributes to OL-ECM interactions and process extension, and considering our previous observation of impaired radial and longitudinal OL process growth in ASMko mice, we quantified CD44 expression specifically along the processes of MBP⁺ mature OLs, avoiding variability from whole-cell extracts at 4 days that contain cells at different maturation stages and could mask differences in mature OLs. Using this approach, we found that SM-treated mature OLs exhibited a 1.8-fold increase in CD44 protein levels along cellular processes, closely matching the 1.6-fold increase observed in ASMko cultured OLs (Fig. 4H). These results demonstrate that SM accumulation is sufficient to elevate CD44 specifically within the processes of mature OLs.

**Figure 5:**
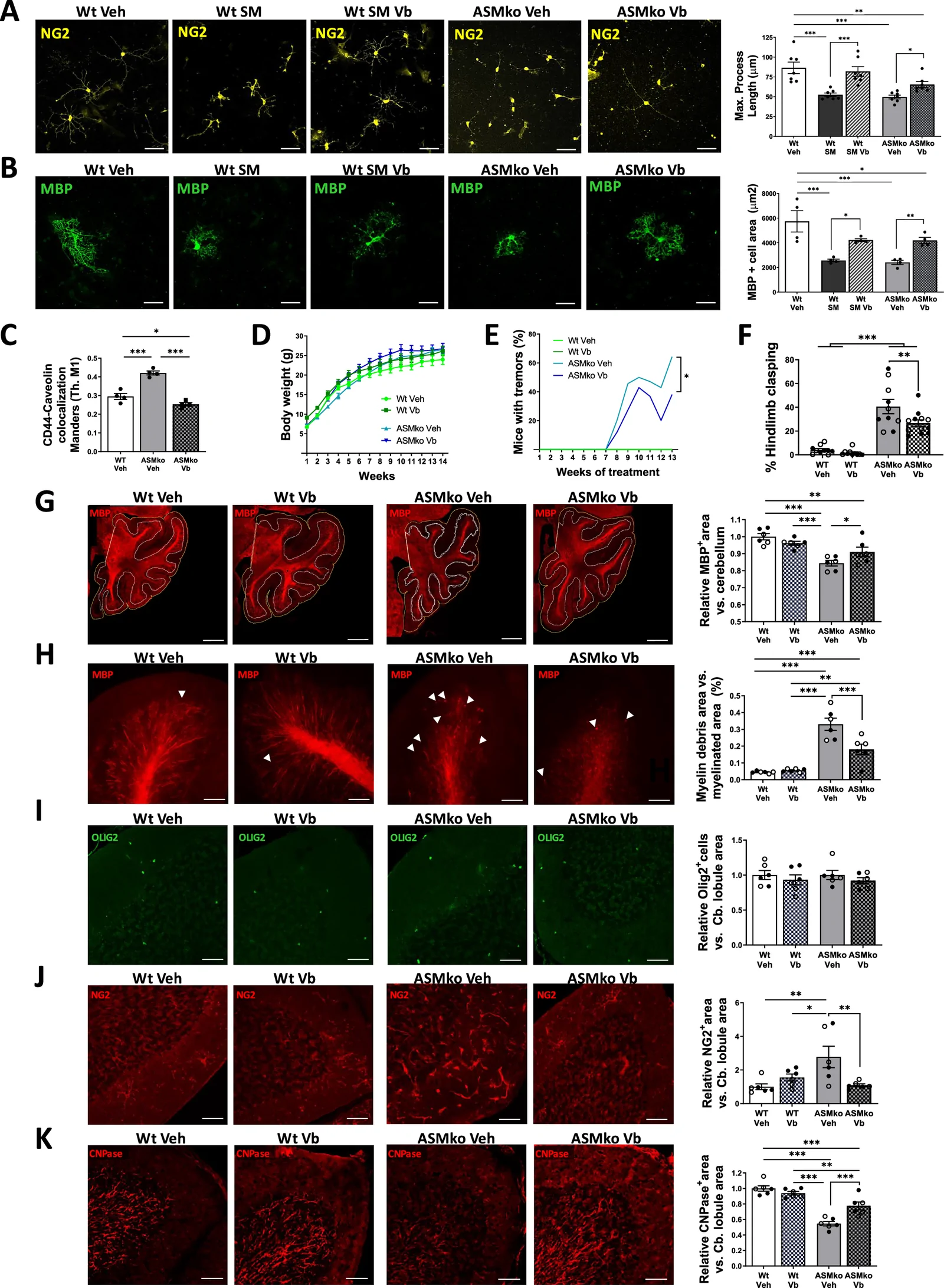
Pharmacological inhibition of CD44 ameliorates OL and myelin abnormalities in vitro and in vivo. (**A**) Representative immunofluorescence images of NG2 in 4-day differentiated OL cultures from WT and ASMko mice treated with vehicle, SM, Vb, or SM+Vb. Data show mean ± SEM length or number of processes in NG2^+^ cells. Statistical analysis was performed using one-way ANOVA followed by Sidak’s post hoc test (*n* = 7; *p*_ASMko Veh vs. Wt Veh_ < 0.001, *p*_Wt+SM vs. Wt Veh_ < 0.001, *p*_ASMko Vb vs. ASMko Veh_ = 0.026, *p*_Wt SM Vb vs. Wt SM_ < 0.001). Additional comparisons: *p*_Wt+SM vs. ASMko Veh_ = 0.684, *p*_Wt+SM Vb vs. Wt Veh_ = 0.506, *p*_ASMko Vb vs. Wt Veh_ = 0.004). Scale bar: 50 µm. (**B**) Representative immunofluorescence images of MBP in 4-day differentiated OL cultures from WT and ASMko mice treated with vehicle, SM, Vb, or SM+Vb. Data show mean ± SEM MBP^+^ cell area. Statistical analysis was performed using one-way ANOVA followed by Sidak’s post hoc test (*n* = 4; *p*_ASMko Veh vs. Wt Veh_ < 0.001, *p*_Wt+SM vs. Wt Veh_ < 0.001, *p*_ASMko Vb vs. ASMko Veh_ = 0.009, *p*_Wt SM Vb vs. Wt SM_ = 0.014). Additional comparisons: *p*_Wt+SM vs. ASMko Veh_ = 0.801, *p*_Wt+SM Vb vs. Wt Veh_ = 0.205, *p*_ASMko Vb vs. Wt Veh_ = 0.020). Scale bar: 50 µm. (**C**) Graph shows mean ± SEM of the Manders M1 coefficient (fraction of CD44 overlapping with caveolin 2), quantified from multiple ROIs within the processes of MBP⁺ mature OLs differentiated for 4 days in vitro from WT and ASMko mice treated with vehicle or with Vb. Statistical analysis was performed using one-way ANOVA followed by Sidak’s post hoc test (*n* = 4; *p*_WtVeh vs. ASMkoVeh_ < 0.001, *p*_Wt Veh vs. ASMko Vb_ = 0.038, *p*_ASMko Veh vs. ASMko Vb_ < 0.001). (**D**) Data show mean ± SEM body weight (g) along the weeks of treatment in WT and ASMko mice receiving vehicle or Vb. Statistical analysis was performed using Kruskal–Wallis test (*n* = 10 for WT Veh, WT Vb, and ASMko Veh; and *n* = 12 for ASMko Vb; *p*_All pairs_ > 0.424). (**E**) Data show mean ± SEM of percentage of mice exhibiting tremors in each experimental group. Statistical analysis was performed using one-way ANOVA followed by Sidak’s post hoc test (*n* = 10 for WT Veh, WT Vb, and ASMko Veh; and *n* = 12 for ASMko Vb; *p*_ASMko Vb vs. ASMko Veh_ = 0.020). (**F**) Data show mean ± SEM of percentage of mice showing hindlimb clasping in each experimental group. one-way ANOVA followed by Sidak’s post hoc test (*n* = 10 for WT Veh, WT Vb, and ASMko Veh; and *n* = 12 for ASMko Vb). Black and white dots denote females and males, respectively; sex ratios were balanced. Statistics: *p*_ASMko Veh vs. Wt Veh_ < 0.001, *p*_Wt Vb vs. Wt Veh_ = 0.63, *p*_ASMko Vb vs. ASMko Veh_ = 0.008, *p*_ASMko Vb vs. Wt Veh_ < 0.001). (**G**) Representative low-magnification (×10) immunofluorescence images of the myelin marker MBP in whole cerebellar sections of WT and ASMko mice treated with vehicle or Vb. Compare the area of the cerebellum, outlined by a continuous yellow line, with the MBP^+^ area delimited by a white dashed line. Data show mean ± SEM percentage of cerebellar area occupied by MBP (*n* = 6). Black and white dots denote females and males, respectively; sex ratios were balanced. Statistics: one-way ANOVA followed by Sidak’s post hoc test *p*_ASMko Veh vs. Wt Veh_ < 0.001, *p*_Wt Vb vs. Wt Veh_ = 0.178, *p*_ASMko Vb vs. ASMko Veh_ = 0.027, *p*_ASMko Vb vs. Wt Veh_ = 0.004, *p*_ASMko Veh vs. Wt Vb_ < 0.001, *p*_ASMko Vb vs. Wt Vb_ = 0.079. Scale bar: 500 μm. (**H**) Representative high-magnification (×25) immunofluorescence images of MBP in the apical regions of cerebellar lobules of WT and ASMko mice treated with vehicle or Vb. Arrowheads show myelin debris. Data show mean ± SEM area occupied by myelin debris with respect to the total MBP+ area in the cerebellum (*n* = 6). Black and white dots denote females and males, respectively; sex ratios were balanced. Data show mean ± SEM area occupied by myelin debris. Statistics: one-way ANOVA followed by Sidak’s post hoc test *p*_ASMko Veh vs. Wt Veh_ < 0.001, *p*_Wt Vb vs. Wt Veh_ = 0.765, *p*_ASMko Vb vs. ASMko Veh_ < 0.008, *p*_ASMko Vb vs. Wt Veh_ = 0.002, *p*_ASMko Veh vs. Wt Vb_ < 0.001, *p*_ASMko Vb vs. Wt Vb_ < 0.001. Scale bar: 50 µm. (**I**–**K**) Representative immunofluorescence images of Olig2 (**I**), NG2 (**J**), and CNPase (**K**) in the apical region of cerebellar lobules from WT and ASMko mice treated with vehicle or Vb. Data show mean ± SEM number of Olig2^+^ + cells (**I**), mean ± SEM area occupied by NG2 (**J**) or CNPase (**K**) relative to the total lobular area analyzed (*n* = 6). Black and white dots denote females and males, respectively; sex ratios were balanced. Statistics: one-way ANOVA followed by Sidak’s post hoc test Olig2: *p*_WtVeh vs. WtVb_ = 0.437, *p*_WtVeh vs. ASMkoVeh_ > 0.999, *p*_WtVeh vs. ASMko Vb:_ = 0.366, *p*_ASMkoVeh vs. ASMkoVb_ = 0.366; NG2: *p*_WtVeh vs. WtVb_ = 0.276, *p*_WtVeh vs. ASMkoVeh_ = 0.002, *p*_WtVeh vs. ASMko Vb:_ = 0.865, *p*_ASMkoVeh vs. ASMkoVb_ = 0.003; CNPase: *p*_WtVeh vs. WtVb_ = 0.234, *p*_WtVeh vs. ASMkoVeh_ < 0.001, *p*_WtVeh vs. ASMko Vb:_ < 0.001, *p*_ASMkoVeh vs. ASMkoVb_ < 0.001. Additional comparisons: Olig2 *p*_WtVb vs. ASMkoVeh_ = 0.437, *p*_WtVb vs. ASMkoVb_ = 0.846; NG2: *p*_WtVb vs. ASMkoVeh_ = 0.022, *p*_WtVb vs. ASMkoVb_ = 0.355; CNPase: *p*_WtVb vs. ASMkoVeh_ < 0.001, *p*_WtVb vs. ASMkoVb_ = 0.004. Scale bar: 50 µm. Asterisks in the graphs of all panels denote significance levels: *P  <  0.05; **P  <  0.01; ***P  <  0.001. Source data are available online for this figure.

**Figure EV2:**
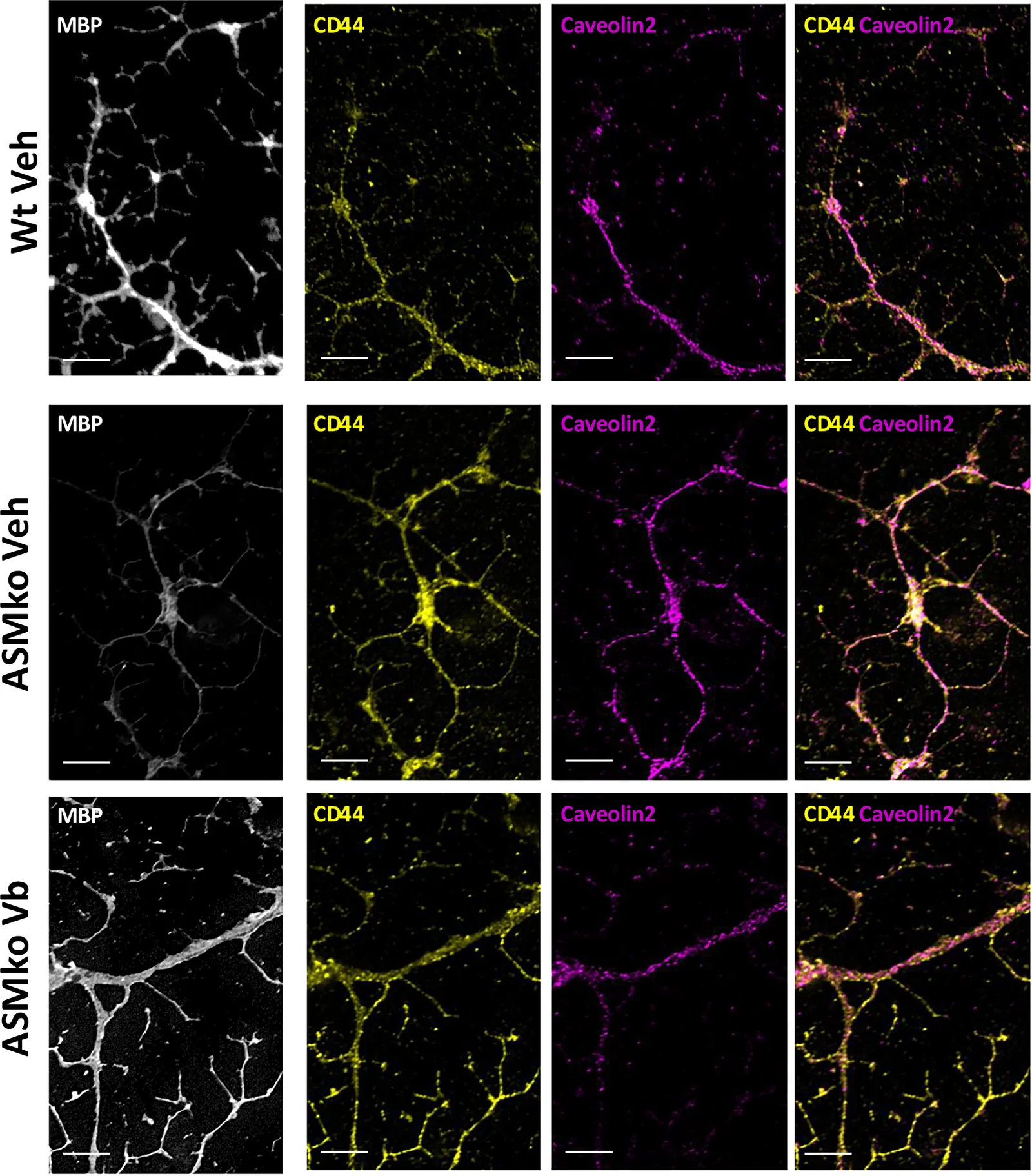
Co-localization of CD44 with the lipid raft marker caveolin 2 in cultured OLs. Immunofluorescence images against CD44 and the lipid raft marker caveolin 2 in MBP⁺ mature OLs differentiated for 4 days in vitro from WT and ASMko mice treated with vehicle or with Vb. Images show high-magnification regions of OL processes (×63) illustrating CD44-caveolin colocalization. Scale bar: 5 µm. Source data are available online for this figure.

The aberrant increase of CD44 expression in ASMko mature OLs suggested that its inhibition could be a viable therapeutic strategy to overcome the observed myelin pathology. To test this, we evaluated the effects of verbascoside (Vb), a small-molecule inhibitor of CD44 signaling (Wang et al, 2020). Treatment of ASMko primary OL cultures with 5 µM Vb during the 4-day differentiation period enhanced morphological maturation, as evidenced by a 32% increase in OPC process length (Fig. 5A) and a 74% expansion of the MBP^+^ membrane area (Fig. 5B). To further validate the role of the SM-CD44 axis, we assessed the rescue potential of Vb in WT cells subjected to exogenous SM. Vb treatment successfully mitigated the inhibitory effects of SM, increasing process length by 56% in NG2^+^ progenitors (Fig. 5A) and expanding the MBP^+^ area by 64% in mature OLs (Fig. 5B). These results indicate that pharmacological inhibition of CD44 improves the morphological features of OLs under conditions of excess SM and ameliorates differentiation defects in ASMko oligodendroglia. Since CD44 signaling has been linked to its localization within lipid raft domains (Oliferenko et al, 1999; Babina et al, 2014), of which SM is a major constituent, we next investigated whether CD44 distribution within lipid rafts is altered in ASMko OLs. Colocalization of CD44 with the lipid raft marker caveolin-2 was increased by 43% in cultured ASMko OLs compared with WT controls (Figs. 5C and EV2). Vb treatment reduced the aberrant colocalization of CD44 with caveolin-2 by 40% in ASMko OLs (Figs. 5C and and EV2).

The rescue effects of Vb in primary ASMko OL cultures warranted further in vivo evaluation. Given that Vb is known to traverse the BBB (Wang et al, 2020), it was administered intraperitoneally to WT and ASMko mice starting at P15, a period coinciding with active OL differentiation and myelination. To ensure safety during this critical developmental window, mice received 50 mg/kg of Vb once daily from P15 to P45, followed by 100 mg/kg thereafter. The treatment was well tolerated, with no overt adverse clinical signs (such as hair loss, diarrhea, or early death) or impact on body weight (Fig. 5D). As analyses revealed no sex-dependent differences in behavioral or histological outcomes, data were pooled across sexes to enhance statistical power. The effects of the treatment on motor behavior were assessed by monitoring tremors throughout the trial and evaluating hindlimb clasping at its endpoint. Vehicle-treated ASMko mice began exhibiting tremors by week 8 of treatment, which escalated until the end of the treatment (14 weeks) (Fig. 5E). Hindlimb clasping, an indicator of cerebellar dysfunction, was also markedly elevated, with a 9.7-fold increase in vehicle-treated ASMko mice relative to WT counterparts (Fig. 5F). Notably, Vb treatment ameliorated motor deficits in ASMko mice, resulting in a significant reduction in tremor incidence over time, as shown in the main longitudinal analysis (Fig. 5E). To further characterize tremor dynamics at the individual level, we performed complementary analyses. A time-to-onset analysis revealed a significant delay in tremor onset in Vb-treated ASMko mice (Appendix Fig. S5A), consistent with the group-level findings. In addition, tremor persistence showed a 23% reduction trend in treated animals, reflecting a lower proportion of symptomatic days per animal (Appendix Fig. S5B). In line with these findings, hindlimb clasping was reduced by 34% following Vb treatment (Fig. 5F).

Consistent with the behavioral improvement, histological analysis of the cerebellum revealed substantial myelin recovery. Vb-treated ASMko mice showed an 8% increase in MBP^+^ area and a 45% reduction in myelin debris when compared with vehicle-treated ASMko mice (Fig. 5G,H). While the total number of Olig2^+^ Ols remained constant (Fig. 5I), Vb treatment corrected the differentiation block: the area occupied by NG2^+^ OPCs decreased by 61% (Fig. 5J) while the area occupied by CNPase^+^ mature OLs increased by 40% (Fig. 5K). Reassuringly, Vb treatment exerted no discernible effects on WT mice across any of the histological measures (Fig. 5G–K), indicating that the drug specifically targets the pathological state associated with ASM deficiency. Interestingly, despite the marked recovery of OL maturation and myelin integrity, Vb treatment did not rescue the reduced internodal length observed in ASMko mice (Appendix Fig. S6). This finding suggests that recovery of OL differentiation is not sufficient to restore all structural features of myelination and raises the possibility that additional extracellular matrix-dependent mechanisms contribute to internodal architecture. Treatment initiation at P15, once much of the early myelination program had already been established, may also have limited the reversibility of this parameter.

## Discussion

The lack of effective therapies for neurological manifestations in ASMD underscores the need to elucidate the specific cellular and molecular mechanisms driving CNS pathology. Traditionally, neurodegeneration in ASMD has been attributed primarily to intrinsic neuronal defects, with dysmyelination and chronic neuroinflammation regarded as secondary phenomena. This paradigm also applies to other neurological lysosomal storage disorders (LSDs) characterized by lipid accumulation. It has been proposed that lysosomal dysfunction and the accumulation of undegraded substrates induce cellular stress, leading to microglial activation, astrogliosis, and axonal degeneration, which subsequently result in myelin loss. Only in leukodystrophies—LSDs caused by mutations in genes involved in oligodendrocyte physiology and myelin composition—has myelin pathology been considered a primary pathological feature (Wolf et al, 2021). However, recent evidence linking myelin debris and microglial activation to neuronal death in the cerebellum of mouse models of neurovisceral ASMD and Niemann Pick type C disease (Gabandé-Rodríguez et al, 2019) challenges this hierarchy. These findings suggest that myelin dysfunction may serve as a primary pathological trigger rather than a consequence of neuronal loss in LSDs.

While a previous study reported an absence of hypomyelination in the corpus callosum of ASM-deficient mice at P16 (Kunkel et al, 2023), our preliminary analyses across multiple brain regions, including the cerebellum, cortex, and hippocampus, revealed distinct oligodendroglial abnormalities at different developmental stages. Given that the cerebellum is critically affected in ASMko mice and offers a unique microenvironment for OL biology, we prioritized this region for in-depth mechanistic and intervention studies to evaluate the contribution of myelin alterations to overall disease progression.

Our findings indicate that at early developmental stages, prior to pathological manifestation, ASMko mice exhibit defects within the OL lineage. These alterations are characterized by an accumulation of immature OLs and a concomitant reduction in mature, myelinating OLs, resulting in a decreased myelinated area within the cerebellum despite a stable OL population. Survival analyses in vivo and in primary OPC cultures suggest this imbalance stems from impaired OL maturation rather than increased cell death. Detailed morphometric examination revealed defects in both OL expansion and myelin assembly. In vitro, immature OLs exhibited shorter radial and longitudinal processes, whereas mature cells were smaller. In vivo, myelin formation was compromised, evidenced by shorter internodes, an increased frequency of OLs stalled at the “contact-forming” stage of myelination, and the presence of aberrant myelin structures. These structural abnormalities likely arise from previously reported alterations in myelin lipid composition (Buccinnà et al, 2009; Ledesma et al, 2011) and/or the observed maturation arrest. As the disease progresses, these primary defects exacerbate rapidly, culminating in overt dysmyelination and the accumulation of myelin debris.

We further demonstrate the cell-autonomous requirement for ASM within the OL lineage. Both the genetic ablation of the ASM-encoding gene (*Smpd1*) and the exogenous addition of SM impair differentiation in isolated OPC cultures. Furthermore, transplantation of OPCs in the mouse retina confirmed that ASMko-derived OPCs generate shorter myelin internodes than WT-derived OPCs, an effect that persists regardless of the genotype of the RGC axons they myelinate.

Transcriptomic profiling further confirmed the presence of defective OL differentiation. RNA-sequencing revealed a notable overlap in gene expression between ASMko OPCs and mature OLs––a sharp contrast to the distinct stage-specific signatures observed in WT mice. While both OPCs and mature OLs from ASMko mice exhibited divergent profiles from their WT counterparts, these transcriptional shifts were far more pronounced in the mature cell population. Functional enrichment analyses (GO terms and KEGG pathways) is ASMko OPCs identified the dysregulation of genes related to lysosomal biology, lipid storage, and sphingolipid metabolism, aligning with expectations for a lysosomal lipid storage disorder such as ASMD. Notably, transcripts related to glial cell differentiation were negatively enriched, providing a molecular basis for the observed maturation arrest. In mature ASMko OLs, the most significantly altered pathways involved ECM binding, receptor-ligand interactions, and membrane lipid rafts.

Among the upregulated ECM-related genes in ASMko mature OLs, we observed a broad dysregulation of extracellular matrix components and receptors, including multiple collagens, laminins, and integrin family members, indicating widespread remodeling of OL-matrix interactions. Within this ECM-associated program, CD44 emerged as a particularly relevant candidate due to its marked upregulation, its established role as a negative regulator of OL differentiation, and its direct therapeutic tractability. Transgenic mice overexpressing CD44 under a myelin-specific promoter exhibit tremors, impaired OL maturation, and progressive CNS dysmyelination (Tuohy et al, 2004), reminiscent of the phenotype we observed in ASMko mice. CD44 is the major receptor for HA, an ECM glycosaminoglycan known to accumulate in dysmyelinated lesions (Back et al, 2005). Both CD44 and HA inhibit morphological OL differentiation (Sato et al, 2022; Liu et al, 2004), and chronically elevated levels have been linked to the accumulation of OPCs (Cargill et al, 2012). Under physiological conditions, CD44 expression is downregulated during OL maturation, whereas it remains aberrantly elevated in ASMko OLs, indicating a failure to suppress this inhibitory signaling pathway. In the CNS, HA is a major component of the extracellular matrix and is predominantly produced by astrocytes, although OPCs and other neural cells can also contribute to its deposition and remodeling. HA provides a dynamic scaffold that regulates oligodendroglial lineage progression, including OPC migration, proliferation, and differentiation during developmental myelination (Yamada et al, 2022). Notably, accumulation of high-molecular-weight HA has been reported in demyelinating lesions and disease contexts such as multiple sclerosis and vanishing white matter disease, where it is associated with impaired OL differentiation and defective remyelination (Back et al, 2005). In these settings, HA-CD44 signaling has been implicated as an inhibitory axis regulating oligodendroglial maturation. Importantly, CD44 is the principal receptor for HA and has been suggested to contribute to the organization of HA-rich matrix domains, thereby influencing ligand presentation and downstream signaling (Tuohy et al, 2004). Accordingly, CD44 upregulation in ASMko oligodendroglial cells may alter both HA signaling and matrix organization. Consistent with this framework, we observed a disorganized HA matrix in the cerebellar white matter of ASMko mice, supporting altered HA-CD44 interactions in vivo. These observations raise the possibility that increased CD44 expression functionally alters oligodendroglial responses to HA-rich environments. To test this hypothesis in a disease-relevant context, we performed in vitro experiments using primary OPC cultures exposed to exogenous SM, a pathological hallmark of ASMD. SM exposure impaired OL differentiation and sustained CD44 upregulation, identifying SM as a previously unrecognized regulator of OL differentiation.

Further research is required to fully elucidate the mechanism by which SM impacts OL differentiation and its specific contribution to the transcriptional landscape identified by RNA sequencing. We propose that elevated SM may modulate CD44 clustering within lipid rafts, potentially enhancing its protein stability and signaling activity. As a primary constituent of these membrane domains, which are known to be structurally altered in the ASMko brain (Galvan et al, 2005), SM levels directly influence raft dynamics. CD44 is enriched in the lipid rafts of various cell types (Oliferenko et al, 1999; Babina et al, 2014), where CD44-HA interactions have been shown to facilitate oncogenic signaling (Bourguignon, 2008). Supporting the hypothesis of an altered association between CD44 and lipid rafts in ASMD, we observed a marked increase in the colocalization of CD44 with the raft marker caveolin 2 in cultured ASMko OLs compared with WT controls.

The detrimental effects of CD44 overexpression on OL maturation suggest that its inhibition could counteract myelin defects in ASMko mice. While the role of CD44 in malignancy has spurred the development of various targeted therapies, including monoclonal antibodies (Avin et al, 2004; Jin et al, 2006), competitive peptides (Hibino et al, 2004), low-MW HA (Slomiany et al, 2009), and HA-conjugated drugs (De Stefano et al, 2011), their clinical utility in the CNS is hampered by high molecular weights and poor BBB penetration. By contrast, Vb, a low-molecular-weight inhibitor of CD44 identified through machine learning-based screening (Wang et al, 2020) and widely used in Chinese traditional medicine, effectively traverses the BBB. By preventing CD44 dimerization and clustering, critical steps for ligand binding and signal transduction (Liu and Sy, 1997; Hartmann et al, 2015), Vb has previously demonstrated efficacy in reducing intracranial glioblastoma growth in mice (Wang et al, 2020). Here, we demonstrate that Vb treatment improves ASMko oligodendroglia differentiation both in vitro and in vivo, ameliorating myelin pathology and behavioral deficits in both male and female ASMko mice following intraperitoneal administration. Given that CD44 is expressed in other CNS populations (Rahman et al, 2020; Peters and Sherman, 2020; Su et al, 2017), its inhibition likely exerts pleiotropic benefits. Indeed, CD44 blockade is known to attenuate reactive astrogliosis and microgliosis (Kruk et al, 2023; Wang et al, 2022), both of which are hallmarks of ASMD. Vb treatment may therefore confer benefits beyond its OL-intrinsic effects. Mechanistically, we propose that SM-induced partitioning of CD44 in OL lipid rafts promotes pathological CD44 dimerization, a process counteracted by Vb.

Overall, our findings identify an essential role for SM in governing OL differentiation. We demonstrate that the lipid imbalances characteristic of ASMD disrupt this process, resulting in a primary dysmyelination that likely serves as the catalyst for microgliosis and progressive neurodegeneration. The stage-specific transcriptional alterations identified in ASMko OPCs and OLs provide a novel molecular framework for understanding ASMD brain pathology and uncover previously unrecognized therapeutic vulnerabilities. Specifically, our results highlight the CD44 signaling axis as a highly tractable target. The preclinical proof-of-concept provided by Vb-mediated inhibition suggests that modulating the ECM-receptor landscape offers a promising strategy for treating the currently intractable neurological manifestations of ASMD.

## Methods

**Table:** Reagents and tools table

Reagent/resource	Reference or source	Identifier or catalog number
**Experimental models**		
C57BL/6 J mice (*Mus musculus*) control	Jackson Lab	RRID:IMSR_JAX:000664
ASM heterozygous C57BL/6 mice	Horinouchi et al, 1995	Kindly donated by Prof. EH Schuchman
**Antibodies**		
Anti-MBP (rat monoclonal) - *Tissue IF 1:100* - *Cells IF 1:1000*	AbD Serotec	Cat. MCA409S
Anti-CNPase (mouse monoclonal) - *Tissue IF 1:200*	Millipore	Cat. MAB326R
Anti-NG2 (rabbit polyclonal) - *Tissue IF 1:500* - *Cells IF 1:500*	Millipore	Cat. AB5320
Anti-Olig2 (goat polyclonal) - *Tissue IF 1:400*	R&D Systems	Cat. AF2418
Anti-GAPDH (mouse monoclonal) - *WB 1:1000*	Abcam	Cat. ab8245
Anti-Caveolin (goat polyclonal) - *Cells IF 1:500*	Abcam	Cat. ab36152
Anti-CD44 (rat monoclonal) *Dil. WB 1:1000;Tissue IF 1:100; Cells IF 1:400*	Thermofisher	Cat. 14-0441-81
Anti-b-Actin (mouse monoclonal) - *WB 1:10.000*	Sigma-Aldrich	Cat. A2228
Anti-PDGFRa (rat monoclonal) - *1:250*	BD Pharmingen	Cat. 558774
Anti-O1 (mouse monoclonal) *1:10*	Sommer and Schachner, 1981	Kindly donated by Dr. C. Paíno
HRP-conjugated goat anti-rabbit IgG (*1:10000)*	DakoCytomation, Glostrup, Denmark	Cat. P0448
HRP-conjugated rabbit anti-mouse IgG (*1:10000)*	DakoCytomation, Glostrup, Denmark	Cat. P0447
Donkey anti-mouse IRDye 680LT IgG (*1:10000)*	LI-COR Biosciences GmbH	Cat. 926-68022
Goat anti-rabbit IRdye 800CW IgG (*1:10000)*	LI-COR Biosciences GmbH	Cat. 926-32211
Alexa 488 Donkey anti-Rat IgG (*1:500)*	ThermoFisher	Cat. A-21208
Alexa 555 Goat anti-Rat IgG *(1:500)*	ThermoFisher	Cat. A-21434
Alexa 488 Donkey anti-Rabbit IgG *(1:500)*	ThermoFisher	Cat. A-21206
Alexa 555 Donkey anti-Rabbit IgG *(1:500)*	ThermoFisher	Cat. A-31572
Alexa 488 Donkey anti-Mouse IgG *(1:500)*	ThermoFisher	Cat. A-21202
Alexa 555 Donkey anti-Mouse IgG *(1:500)*	ThermoFisher	Cat. A-31570
Alexa 488 Donkey anti-Goat IgG *(1:500)*	ThermoFisher	Cat. A-11055
Alexa 555 Donkey anti-Goat IgG *(1:500)*	ThermoFisher	Cat. A-21432
Alexa 647 Donkey anti-Goat IgG *(1:500)*	ThermoFisher	Cat. A-21447
Streptavidin-HRP	Sigma	Cat. RABHRP3
Anti-A2B5-conjugated magnetic beads	Miltenyi Biotec	Cat. 130-093-388
**Chemicals, enzymes, and other reagents**		
HBSS + /+	Gibco	Cat. 14185-045
Neural Tissue Dissociation Kit (P)	Miltenyi Biotec	Cat. 130-092-628
Bovine Serum Albumin	PanReac AppliChem	Cat. A1391
Ethylenediaminetetraacetic acid (EDTA)	Sigma	Cat. E6758
LS columns	Miltenyi Biotec	Cat. 130-042-401
Neuro Medium	Miltenyi Biotec	Cat. 130-093-570
MACS® NeuroBrew-21 w/o vitamin A	Miltenyi Biotec	Cat. 130-093-566
Glutamine	ThermoFisher Scientific	Cat. 25030081
D-glucose	Sigma	Cat. G8769
Platelet-derived growth factor-AA (PDGF-AA)	Pepro-Tech	Cat. 100-13 A
Fibroblast growth factor-2 (FGF-2)	Pepro-Tech	Cat. 100-18B
Poly-L-lysine (0.5 mg/ml)	Sigma	Cat. P2636
Laminin (10 μg/ml)	Sigma	Cat. L-2020
Hyaluronic acid sodium salt MW 1.5-1.8 x 10E6 Da	Sigma	Cat. 53747
Paraformaldehyde (PFA)	Sigma	Cat. 158127
Phosphate-buffered saline (PBS)	Gibco	Cat. 14190-094
Sucrose	Sigma	Cat. 1.07654.1000
Triton X-100	Sigma	Cat. T8787
Hyaluronic Acid Binding Protein Biotinylated (1 μg/ml)	Sigma	Cat. 385911
4’,6-diamidino-2fenilindol (DAPI)	Calbiochem	Cat. 267298
OsO₄	Sigma	Cat. 75632
Epoxy resin	Sigma	Cat. 31190
Sphingomyelinase (Smase C)	Merck	Cat. S9396
Thesit	Sigma	Cat. 88315
Acid sphingomyelinase	Sino Biological	Cat. 50749-M08B
Verbascoside	MedChemExpress	Cat. HY-N0021
Verbascoside	Kaimosi Biochem	Cat. BP0124
DeadEnd™ Fluorometric TUNEL (TdT-mediated dUTP Nick-End Labeling)	Promega	Cat. G3250
Maxwell® 16 LEV simplyRNA kit	Promega	Cat. AS1270
PierceTM BCA Protein Assay Kit	ThermoFisher Scientific	Cat. 23225
Pierce^TM^ ECL Western Blotting Substrate	Thermo Fisher Scientific	Cat. 34577
**Software**		
Fiji	https://imagej.net/software/fiji/	N/A
GraphPad Prism 9 software	GraphPad Software, La Jolla, CA, USA	N/A
R	https://www.Rproject. org	N/A
MetaboAnalyst 5.0	https://www.metaboanalyst.ca/	N/A
Illumina NextSeq 2000 platform	Illumina Inc., San Diego, CA, USA	N/A
STRING (v11.5)	https://string-db.org/	N/A
Huygens Professional	Scientific Volume Imaging, The Netherlands	N/A
Biorender	BioRender.com	N/A
NIH Bioart	https://bioart.niaid.nih.gov/	N/A
**Other**		
Confocal Zeiss LSM710 vertical microscope	Zeiss	N/A
Confocal Zeiss LSM900 microscope	Zeiss	N/A
Axiovert 200 M inverted wide-field microscope	Zeiss	N/A
Transmission electron microscope JEM1010	Jeol,1992	N/A
DM IL LED inverted microscope	Leica Microsystems	N/A
Agilent 2100 Bioanalyzer	Agilent	N/A
Amersham Imager 680 (CCD camera)	Amersham	N/A
Odyssey Scanner	LI-COR Biosciences	N/A

### Mice

Breeding colonies were established from ASM-heterozygous C57BL/6 mice (Horinouchi et al, 1995), kindly donated by Prof. Schuchman (Icahn School of Medicine at Mount Sinai, New York). All animal procedures were designed and reported in strict accordance with ARRIVE guidelines. All procedures involving mice were reviewed and approved by the Comunidad de Madrid (PROEX 014.1/24 and PROEX044_19), and were conducted in compliance with specific European Union guidelines (Directive 2010/63/EU).

### Human samples

Formaldehyde-fixed brain tissue from a 3-year-old ASMD-type A patient, and a 3-year-old control child was donated by the Wylder Nation Foundation (http://wyldernation.org), and the Fundacion CIEN brain bank (http://bt.fundacioncien.es), respectively. Informed consent was obtained from all subjects, and the experiments conformed to the principles set out in the WMA Declaration of Helsinki and the Department of Health and Human Services Belmont Report. For immunofluorescence in human tissue, sections were first deparaffinized by pre-heating at 60 °C for 15 min in a dry heat incubator, followed by two consecutive 15-min washes in xylene. Rehydration was performed through a series of 5-min washes in decreasing concentrations of ethanol (100%, 96%, 90%, 70%, and 50%). Antigen retrieval was subsequently carried out by boiling the sections in 10 mM citrate buffer (pH 6.0) at 110 °C for 1 min. After cooling to room temperature, the immunofluorescence protocol was performed.

### OPC isolation from the mouse brain

Oligodendrocyte precursor cells (OPCs) were obtained from the cerebral cortices of postnatal day 5 (P5) C57BL/6 mice *via* magnetic-activated cell sorting (MACS; Miltenyi Biotec) as described (Melero-Jerez et al, 2021; Gómez-Martín et al, 2025). Briefly, brains were dissected, and meninges were removed in ice-cold Hank’s balanced salt solution with Ca^2+^ and Mg^2+^ (HBSS^+/+^, Gibco). Cortices were then dissociated for 25 min at 37 °C with gentle agitation in HBSS lacking Ca^2+^ and Mg^2+^ (HBSS^−/−^). The tissue was mechanically triturated in HBSS^−/−^ and further enzymatically digested for 25 min at 37 °C using the Neural Tissue Dissociation Kit P (Miltenyi Biotec). The resulting cell suspension was triturated using serial pipetting (p1000 and p200 tips), filtered through a 70-μm nylon strainer (BD Falcon), and centrifuged at 300× *g* for 15 min. The cell pellet was incubated for 30 min at 4 °C with A2B5-conjugated magnetic beads (Miltenyi Biotec) diluted 1:200 in wash buffer (2 mM sodium pyruvate, 0.5% bovine serum albumin [BSA; PanReac AppliChem], and 2 mM ethylenediaminetetraacetic acid [EDTA], pH 7.3 [Sigma-Aldrich]). Following washing, cells were loaded onto LS columns (Miltenyi Biotec) within a magnetic field. OPCs were eluted in Neuro Medium supplemented with 2% MACS® NeuroBrew-21 without vitamin A (both from Miltenyi Biotec), 0.6% penicillin/streptomycin (Sigma-Aldrich), 200 mM glutamine (Gibco), 25 mM D-glucose (Normapur), and 10 ng/ml of platelet-derived growth factor-AA (PDGF-AA; Merck) and fibroblast growth factor-2 (FGF-2; Pepro-Tech). Isolated OPCs (purity ~90%) were utilized for intravitreal injection or seeded onto coverslips coated with 0.5 mg/ml poly-L-lysine and 10 μg/ml laminin (both from Sigma-Aldrich).

### Immunofluorescence

Mouse brains were dissected and fixed in 4% paraformaldehyde (PFA) containing 0.12 M sucrose, followed by cryoprotection in 30% sucrose phosphate-buffered saline (PBS). The tissue was embedded in optimal cutting temperature compound (Tissue-Tek; Sakura Finetek), frozen, and sectioned sagittally at 40 μm using a cryostat (Leica, CM1950 Ag Protect freezing). To minimize non-specific binding, sections were incubated for 1 h at room temperature (RT) in PBS supplemented with 2% BSA and 0.5% Triton X-100 (Sigma-Aldrich). Primary antibodies were applied overnight at 4 °C, followed by incubation with appropriate Alexa Fluor-conjugated secondary antibodies (Thermo Fisher Scientific). For hyaluronan (HA) detection, sections were incubated overnight with 1 μg/ml Hyaluronic Acid Binding Protein Biotinylated (HABP) (Sigma-Aldrich) at 4 °C, followed by Streptavidin-HRP (Sigma-Aldrich) for 1 h. Nuclei were counterstained for 20 min with 4’, 6-diamidino-2-phenylindole (DAPI; Calbiochem), and sections were mounted using Mowiol-Dabco (Calbiochem).

Cultured oligodendroglia were fixed for 10 min in 4% PFA containing 0.12 M sucrose. Following fixation, cells were permeabilized and antigen-blocked for 1 h at RT in PBS containing 2% BSA and 0.1% Triton X-100. The cells were then incubated sequentially with primary antibodies (overnight at 4 °C) and Alexa Fluor-conjugated secondary antibodies (1 h at RT).

### Image acquisition and analysis

The following microscope systems were utilized according to experimental requirements: LSM 710 and LSM 900 confocal microscopes (Carl Zeiss), an Axiovert 200 M inverted wide-field microscope (Zeiss, PCO-equipped), and a DM IL LED inverted microscope (Leica Microsystems) for phase-contrast imaging. Image analysis and processing were performed using Fiji software (Schindelin et al, 2012). Graphical representations and schematic figures were generated using BioRender and NIH Bioart.

### Analyses of OLs in vitro

To assess early morphological changes during cell adhesion, MACS-purified OPCs were plated onto poly-L-lysine (PLL)/laminin-coated coverslips. After 2 h, cultures were gently washed, and the medium was carefully replaced to remove non-adherent cells. Adherent cells were imaged by phase-contrast microscopy (Leica), and cell polarity was quantified by calculating the polarity index (cell length/cell width ratio). A minimum of 50 cells per field was analyzed, and cells were subsequently classified into predefined polarity index ranges for distribution analysis. To evaluate OPC adhesion on hyaluronic acid (HA), MACS-purified OPCs were plated onto PLL/HA-coated coverslips (0.2 μg/μL HA, coated overnight at 4 °C). Because adhesion to HA was slower than to laminin, morphological analyses were performed 4 h after plating. Adherent cells were imaged by phase-contrast microscopy, and the polarity index was determined as described above. For adhesion experiments in differentiated oligodendrocytes (OLs), MACS-purified OPCs were first differentiated in vitro for 4 days. Cultures were then dissociated using papain and replated onto PLL/HA-coated coverslips. Cell morphology was assessed 7 h after replating by phase-contrast microscopy, and the polarity index was calculated as described above. To analyze subsequent morphological changes on HA, cultures were fixed 2 days after replating and processed for immunofluorescence analysis.

For differentiation assays, MACS-purified OPCs were seeded at 30,000 cells per coverslip. After 24 h, the elution medium was replaced with differentiation medium (lacking PDGF-AA and FGF-2). To simulate the progressive lipid accumulation characteristic of ASMD, cultures were treated with SM at concentrations of 20 and 40 µM on the 1st and 3rd days of differentiation, respectively, to avoid toxicity. The dosing regimen was adapted from previously published protocols in neuronal cultures (Arroyo et al, 2014). After 4 days of differentiation, cells were fixed for immunofluorescence analysis. Morphological parameters were assessed in NG2⁺ OPCs and MBP⁺ mature OLs. In NG2⁺ cells, radial and longitudinal growth were assessed by quantifying the number and length of cell processes. In MBP⁺ mature OLs, morphology was determined by measuring the total MBP⁺ area in the cells.

For apoptosis assays, PFA-fixed OL cultures were processed using the DeadEnd™ Fluorometric TUNEL (TdT-mediated dUTP Nick-End Labeling) System (Promega; # G3250).

### In vivo analyses of OL and myelin

The OL lineage was characterized using stage-specific markers, including NG2, CNPase, MBP, and Olig2. Myelin integrity and signs of degeneration were evaluated *via* immunostaining for MBP in the cerebellum of mice at P10, P20, and P90. The myelinated area was quantified relative to the total cerebellar area using stitched ×5 magnification confocal images. For detailed morphological analyses (including internodal length, MBP^+^ debris, and MBP^+^ contact-forming OLs), we focused on the apical regions of the cerebellar lobules, where lower myelin density facilitates the detection of pathological changes. Images were acquired using an Axiovert 200 M inverted wide-field microscope (Zeiss). At P20 and P90, analysis was performed on ×25 magnification images. Due to the nascent state of myelination at P10, high-resolution analyses were instead conducted on maximum-intensity projections of 15 µm z-stacks acquired at ×40 magnification. Internodal length was assessed in regions with lower myelin density (apical lobular areas), where individual fibers are clearly resolvable. Only clearly distinguishable isolated fibers were included in the analysis, and highly compact regions were excluded. Internodal length was measured across five images from lobules per cerebellum, with at least 30 myelinated fibers analyzed per image. For each mouse, the area occupied by MBP^+^ debris ( ≥ 1 µm²) and the density of contact-forming OLs were quantified in at least five images acquired from the apical, myelinated regions of cerebellar lobules.

### Myelin ultrastructural analysis by electron microscopy

Mice were transcardially perfused with PBS followed by a fixative solution containing 4% PFA and 2% glutaraldehyde in PBS. Brains were post-fixed overnight in 4% PFA, cryoprotected in 30% sucrose for 24 h, and sectioned into 200-µm-thick slices. Sections were subsequently treated with 2% OsO₄, en bloc stained with 2% uranyl acetate, dehydrated through a graded series of ethanol, and embedded in epoxy resin. Samples were mounted between acetate sheets and, after allowing polymerization at 60 °C for 48 h, cerebellar sections were examined using a transmission electron microscope (JEM1010, Jeol). Myelinated fibers within the cerebellar white matter were randomly sampled and imaged using a CMOS 4k TemCam-F416 camera (TVIPS, Gauting). The G-ratio, defined as the ratio of the inner axonal diameter to the total outer fiber diameter (including the myelin sheath), was calculated from images taken at ×8000 magnification. Analysis was restricted to transverse fiber sections with well-preserved myelin morphology; axons exhibiting aberrant or disrupted myelin were excluded from the G-ratio calculation. At least three mice per genotype were analyzed, with a minimum of 20 randomly selected images acquired per mouse.

### Intravitreal OPC transplantation

OPC purification was performed *via* MACS as described above, and intravitreal injections were conducted in 2-month-old mice following established protocols (Setzu et al, 2004; Nocera et al, 2024). Briefly, adult mice were anesthetized by an intraperitoneal injection of ketamine (87.5 mg/kg) and xylazine (12.5 mg/kg). Intravitreal delivery of OPCs was performed under direct visualization using a 33-gauge needle coupled to a 5 μL glass syringe (Hamilton). A total of 50,000 OPCs suspended in 1 μL of vehicle were delivered adjacent to the retinal ganglion cell (RGC) layer.

Five weeks post-transplantation, mice were transcardially perfused. Eyes were harvested, fixed in 4% PFA for 2 h, and subsequently transferred to PBS. The retinae were dissected free from the sclera, processed for immunohistochemistry, radially incised (4–5 cuts), flat-mounted, and examined by confocal microscopy to evaluate OPC integration and myelination. Caspr immunostaining (for paranodes) in combination with MBP was used to confirm that complete internodal segments were analyzed.

### Western blotting

Protein extracts from brain tissue or cultured cells were prepared in either MES-based lysis buffer (20 mM 2-morpholinoethanesulfonic acid, pH 7.0; 150 mM NaCl; 1 mM EDTA) or RIPA buffer (25 mM Tris-HCl, pH 7.6; 150 mM NaCl; 1% NP-40; 1% sodium deoxycholate; 0.1% SDS). Both buffers were supplemented with 2 mM protease inhibitors (cOmplete^TM^, Sigma-Aldrich) and phosphatase inhibitors (Sigma-Aldrich). Samples were resolved *via* SDS–PAGE and transferred onto nitrocellulose or PVDF membranes. Following blocking, membranes were probed with primary antibodies and subsequently incubated with HRP-conjugated (DakoCytomation) or fluorescent secondary (Fluorescent IrDye, LICOR Biosciences) antibodies. Signal detection was performed using enhanced chemiluminescence (Pierce^TM^ ECL Western Blotting Substrate, Thermo Fisher Scientific) or fluorescence-based methods (IrDye, LicorBiosciences) and captured using a CCD camera (Amersham Imager 680) or an Odyssey Scanner (LI-COR Biosciences). Band intensities were quantified by densitometry using FIJI software.

### Analysis of hyaluronan fiber-like structures

HA was visualized by immunofluorescence in the deep white matter of the cerebellum, a region enriched in mature OLs. For morphological quantification, the lengths of the five longest HABP+ fiber-like structures associated with MBP+ processes were measured per image, with five images analyzed per cerebellum. HA was quantified indirectly *via* HABP labeling, as HA is a polysaccharide and cannot be targeted by traditional antibodies.

### CD44-caveolin colocalization analysis

Colocalization of CD44 with the lipid raft marker caveolin 2 was evaluated in MBP⁺ mature OLs following 4 days of in vitro differentiation. For each MBP⁺ cell, high-resolution confocal/STED images of the intermediate region of the OL processes were acquired using a Zeiss LSM900 equipped with a ×63/1.4 NA oil objective (×2.5 digital zoom). Image stacks were collected under acquisition conditions satisfying Nyquist sampling (lateral sampling < 80 nm; z-step 0.13 µm) and were deconvolved using Huygens Professional software *via* the CMLE algorithm (40 iterations, automatic PSF, and channel-specific SNR). Deconvolved images were processed in FIJI, and colocalization between CD44 and caveolin 2 was quantified with the BIOP-JACoP plugin. For quantification, three regions of interest (ROIs) were randomly selected within the intermediate process regions per image. The Manders’ M1 coefficient threshold (fraction of CD44 overlapping with caveolin 2) was computed using Otsu thresholding for both channels. The colocalization value for each image was defined as the average of the three ROIs, with a minimum of four images analyzed per biological replicate.

### Quantification of total SM by enzymatic assay

Total SM levels were determined using an enzymatic assay adapted from previously described methods (Hojjati and Jiang, 2006). Lipid extracts were evaporated in the presence of Thesit detergent, and SM was enzymatically hydrolyzed and converted into peroxide *via* sequential incubation with sphingomyelinase (Merck), alkaline phosphatase, and choline oxidase. The resulting peroxide was quantified fluorometrically through a peroxidase-dependent reaction utilizing homovanillic acid as a substrate.

### RNA-sequencing analysis of immature and mature OLs

Immature OPCs and mature OLs were isolated *via* sequential immunopanning from P20 WT and ASMko mice (Emery and Dugas, 2013; Barres, 2014). Briefly, mice were transcardially perfused with PBS, and brains were dissected and enzymatically dissociated using a papain-based kit (Miltenyi Biotec). Single-cell suspensions were sequentially immunopanned to deplete microglia and endothelial cells, and to enrich for OPCs (PDGFRα⁺) or mature OLs (O1⁺). Total RNA was extracted using the Maxwell® 16 LEV simplyRNA kit (Promega). RNA integrity was verified using the RNA 6000 Nano Bioanalyzer 2100 assay (Agilent). Library construction and single-read sequencing were conducted at the Centre for Genomic Regulation (Barcelona, Spain) using Illumina NextSeq 2000 technology. Libraries were prepared from 100 ng of total RNA, utilizing oligo-dT beads for poly-A mRNA enrichment. Strand-specific cDNA libraries were generated with unique dual indices and molecular identifiers to ensure high-fidelity quantification.

### Transcriptome profiling

Libraries were validated on an Agilent 2100 Bioanalyzer and sequenced as single-end 50 bp reads on an Illumina NextSeq 2000 platform, yielding 32–35 million reads per sample. Following quality control, reads were aligned to the mouse reference genome (GRCm39), with a mapping efficiency of ~90%. Gene-level counts were generated using HTSeq-count (v0.11.2) in intersection-strict mode. Differential expression analysis was conducted using DESeq2 in R, utilizing a negative binomial distribution to model gene and transcript expression. Raw sequencing data have been deposited in the European Nucleotide Archive under accession PRJEB57840. Principal Component Analysis (PCA) and heatmap analysis of the 1000 most variable transcripts (ANOVA) were performed using MetaboAnalyst 5.0 software (Pang et al, 2021). Gene Set Enrichment Analysis (GSEA) was executed using predefined gene sets from the Gene Ontology (GO) and Kyoto Encyclopedia of Genes and Genomes (KEGG) databases. Differentially expressed genes with *P* values < 0.05 were chosen, and the log_2_ of the fold changes were used as input. Enrichment statistics and normalized enrichment scores (NES), which account for gene set size and inter-gene correlations, were computed using the R package clusterProfiler and enrichplot, applying the Benjamini-Hochberg procedure to control false discovery rate. For graphical representation, significantly enriched terms/pathways (adjusted *P* < 0.05) were systematically filtered to remove redundant or biologically unrelated categories, and a subset of terms with relevance to oligodendrocyte lineage biology was visualized with GraphPad Prism 9 software (GraphPad Software, La Jolla, CA). Finally, protein–protein interaction networks were constructed using STRING (v11.5), with functional modules identified through the STRING clustering algorithm.

### Verbascoside treatment

#### In vitro

MACS-purified OPCs were plated at 30,000 cells per coverslip and induced to differentiate as previously described. Verbascoside (Vb) was added to the medium at a final concentration of 5 µM on days 1 and 3 of the differentiation protocol. At the end of day 4, cells were fixed and processed for immunofluorescence. Morphological parameters of differentiation were quantified in NG2⁺ OPCs and MBP⁺ OLs as established in previous sections.

#### In vivo

Vb or saline (control) was administered intraperitoneally to WT and ASMko mice once daily from P15 for 14 weeks. To mitigate potential developmental toxicity, mice received 50 mg/kg once daily from P15 to P45, followed by 100 mg/kg for the remainder of the treatment period. All groups were monitored daily for adverse clinical signs.

### Behavioral analysis

Motor dysfunction was evaluated by monitoring tremors and hindlimb clasping. Tremors were monitored daily throughout the treatment period. Prior to drug administration, each animal was observed at rest for 5 s. To minimize false positives, tremors were only recorded when sustained, clearly noticeable shaking was present. The daily incidence of tremors was recorded for each group, and data were expressed as the weekly average proportion of affected animals. Hindlimb clasping was assessed at the conclusion of the treatment period. Mice were suspended by the base of the tail for 20 s at a height of 30 cm above a stable platform. Typically, hindlimbs are fully spread and moving; therefore, the duration of clasping, defined as one or both hindlimbs drawn toward the abdomen, was recorded.

### Statistics

The number of mice used was selected on the basis of previous phenotyping analyses conducted in the same model and by calculating the statistical power of the experiment. Mice were genotyped and, according to the genotype, randomly assigned to the experimental groups. Investigators assessing and measuring results were blinded to the intervention. Quantitative data were obtained from a minimum of three independent experimental groups and are presented as mean ± SEM. Data distribution and normality were assessed using the Shapiro–Wilk test. For comparisons between two groups, a two-tailed Student’s *t* test was used for normally distributed data, whereas the Mann–Whitney *U* test was employed for non-parametric data. For experiments involving more than two groups, normally distributed datasets were analyzed by one-way ANOVA followed by Sidak’s post hoc test, whereas non-parametric data were evaluated using the Kruskal–Wallis test followed by Dunn’s post hoc test. In instances where data were normalized to a control (fold change = 1), a one-sample *t* test was performed to determine if the mean significantly differed from the hypothetical value of 1. Correlation analyses were performed by computing Manders’ correlation coefficients. To compare rates of change, linear regression analyses were conducted, and slopes were compared using 95% confidence intervals. Statistical significance was defined as *P*  <  0.05. In all figures, asterisks denote significance levels: *P  <  0.05; **P  <  0.01; ***P  <  0.001. GraphPad Prism 9 software was used for all statistical analysis.

## Supplementary information

Peer Review File

Appendix

Source data Fig. 1

Source data Fig. 2

Source data Fig. 3

Source data Fig. 4

Source data Fig. 5

Figure EV1 Source Data

Figure EV2 Source Data

Appendix Figure Source Data

Expanded View Figures

## Data Availability

The raw sequencing dataset generated in this study has been deposited in the European Nucleotide Archive under accession PRJEB57840. The source data of this paper are collected in the following database record: biostudies:S-SCDT-10_1038-S44321-026-00503-8.
