# Supplementary material for: Targeting CD44 reverses sphingomyelin-induced oligodendrocyte maturation arrest in acid sphingomyelinase deficiency
Source: EMBO Mol Med. 2026 Aug 12;18(9):3767–89. doi: 10.1038/s44321-026-00503-8 (PMC13562706; doi:10.1038/s44321-026-00503-8)
Supplement: Supplementary file 1 [file 44321_2026_503_MOESM11_ESM.pdf]

Expanded View Figures

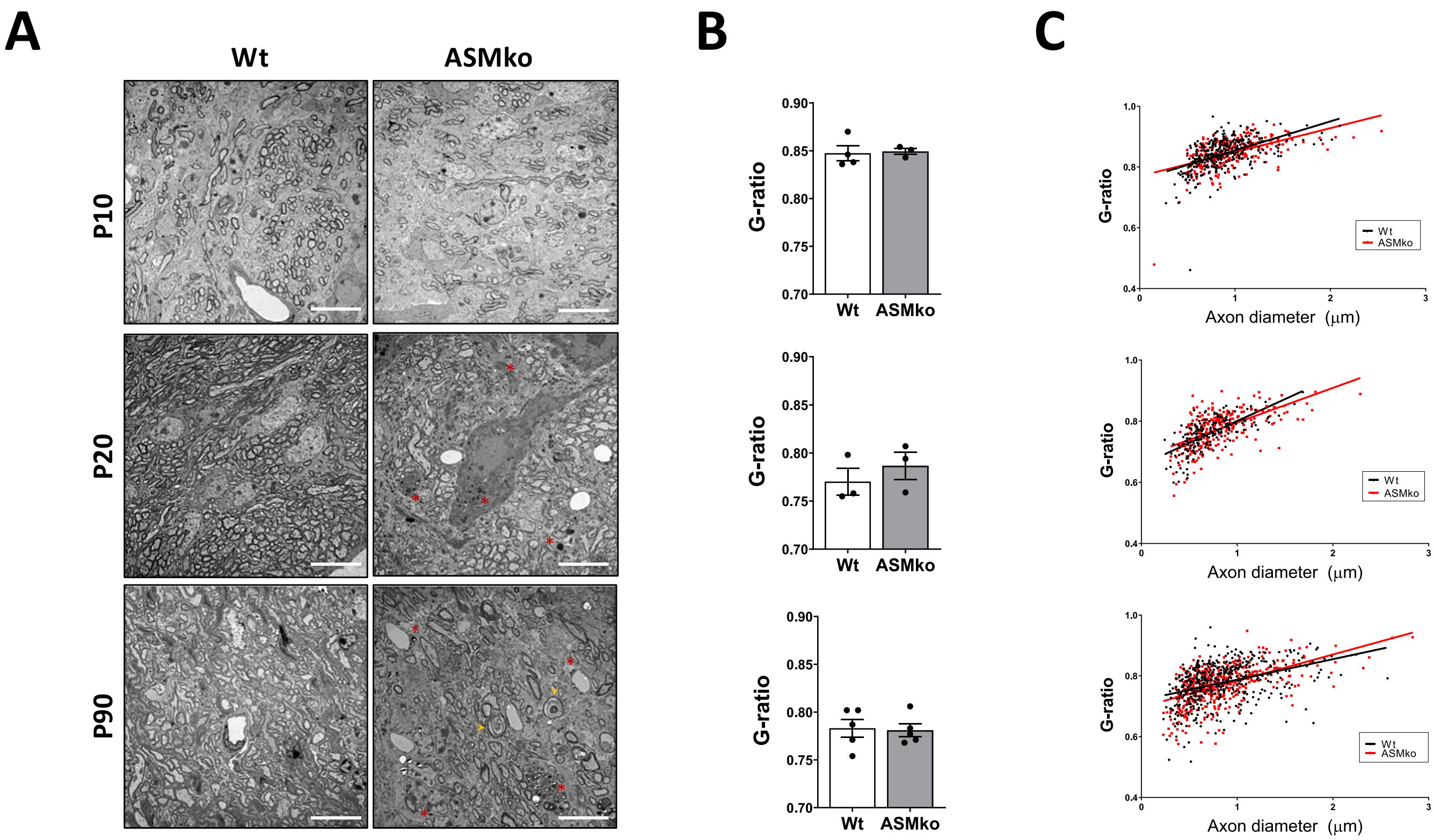

Figure EV1. Myelin characterization in the cerebellum of WT and ASMko mice.

(**A**) Electron micrographs of white matter from cerebellar lobules of WT and ASMko mice at P10, P20, and P90. Red asterisks indicate intracellular accumulation of electron-dense lipid droplets in ASMko samples; by P90, signs of fiber degeneration and axonal loss become evident (yellow arrowheads). Only transverse sections of myelinated fibers with intact axonal morphology and normally compacted myelin were included for g-ratio measurements. Scale bar: 10 µm. (**B**) Graphs show mean ± SEM g-ratio of myelin thickness of wt and ASMko mice at P10, P20, and P90; P10: Statistical analysis was performed using two-tailed *t* test. $n = 4$ wt, 3 ASMko, $P = 0.857$; P20: $n = 3$ per genotype, $P = 0.459$; P90: $n = 5$ per genotype, $P = 0.853$). (**C**) Scatter plot showing the relationship between g-ratio and axon diameter (< 3 µm) in wt and ASMko mice. Linear regression analyses reveal no significant differences between genotypes (P10: $P = 0.144$; P20: $P = 0.054$; P90: $P = 0.059$). More than 150 fibers were analyzed from at least three mice per genotype. Source data are available online for this figure.

 © The Author(s)

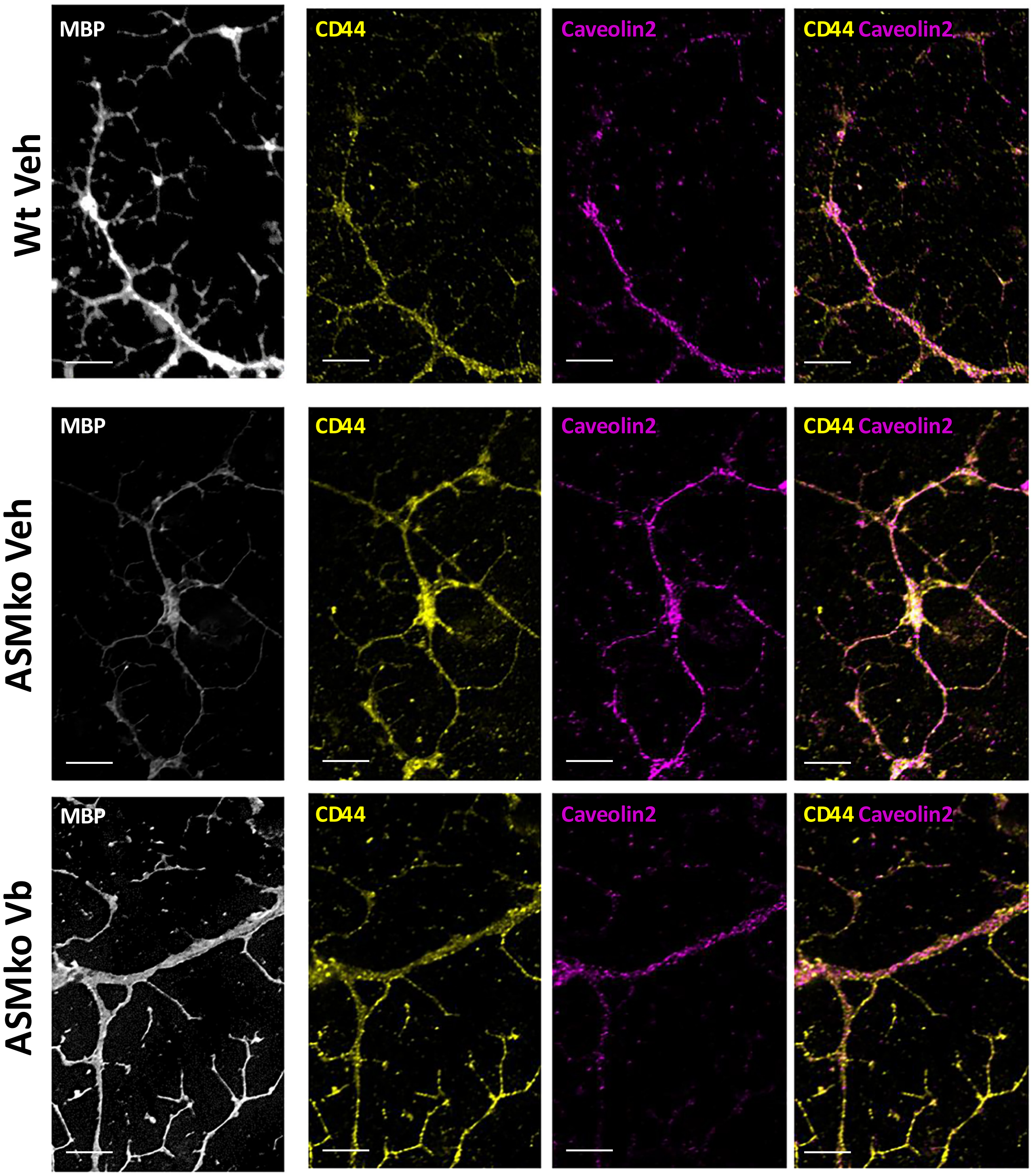

Figure EV2. Co-localization of CD44 with the lipid raft marker caveolin 2 in cultured OLs.

Immunofluorescence images against CD44 and the lipid raft marker caveolin 2 in MBP⁺ mature OLs differentiated for 4 days in vitro from WT and ASMko mice treated with vehicle or with Vb. Images show high-magnification regions of OL processes (×63) illustrating CD44-caveolin colocalization. Scale bar: 5 µm. Source data are available online for this figure.
