# Supplementary material for: Targeting CD44 reverses sphingomyelin-induced oligodendrocyte maturation arrest in acid sphingomyelinase deficiency
Source: EMBO Mol Med. 2026 Aug 12;18(9):3767–89. doi: 10.1038/s44321-026-00503-8 (PMC13562706; doi:10.1038/s44321-026-00503-8)
Supplement: Supplementary file 2 [file 44321_2026_503_MOESM1_ESM.pdf]

EMBO Molecular Medicine

Targeting CD44 reverses sphingomyelin-induced oligodendrocyte maturation arrest in ASM deficiency

Marta Guerrero-Valero, Elena Melgarejo, Miguel Marchena, Beatriz Fernández-Gómez, Jaime Mulero-Franco, Fernando de Castro, Edward Schuchman, and Maria Dolores Ledesma

Corresponding authors: Maria Dolores Ledesma (dledesma@cbm.csic.es) , Marta Guerrero-Valero (mguerr25@ucm.es)

Review Timeline:

Submission Date:	25th Feb 26
Editorial Decision:	1st Apr 26
Revision Received:	3rd Jul 26
Editorial Decision:	20th Jul 26
Revision Received:	23rd Jul 26
Accepted:	29th Jul 26

Editor: Jingyi Hou

Transaction Report:

(Note: With the exception of the correction of typographical or spelling errors that could be a source of ambiguity, letters and reports are not edited. Depending on transfer agreements, referee reports obtained elsewhere may or may not be included in this compilation. Referee reports are anonymous unless the Referee chooses to sign their reports.)

1st Apr 2026

Dear Dr. Ledesma,

Thank you for submitting your work to EMBO Molecular Medicine. We have now received evaluations from the two reviewers who agreed to assess your manuscript. As you will see from the reports below, the reviewers recognize the potential significance of your study. However, they also raise several concerns that must be addressed in a major revision.

Given the clarity of the reviewers' recommendations, I will not restate all the points here. In particular, we encourage the inclusion of at least some human data to strengthen the clinical relevance of the study, as suggested by Reviewer #2. Both reviewers also provided additional comments on the use of human data during our pre-decision cross-commenting process, which are included below following the reviewer reports. All the other concerns need to be carefully addressed as well.

As you may already know, our editorial policy allows in principle a single round of major revision and it is therefore essential to provide responses to the reviewers' comments that are as complete as possible. Please feel free to contact me in case you would like to discuss in further detail any of the issues raised by the reviewers.

EMBO Molecular Medicine has a "scooping protection" policy, whereby similar findings that are published by others during review or revision are not a criterion for rejection. Should you decide to submit a revised version, I do ask that you get in touch after three months if you have not completed it, to update us on the status.

Please also contact us as soon as possible if similar work is published elsewhere. If other work is published, we may not be able to extend the revision period beyond three months.

Please read below for important editorial formatting and consult our author's guidelines for proper formatting of your revised article for EMBO Molecular Medicine.

I look forward to receiving your revised manuscript.

Sincerely,
Jingyi

Jingyi Hou
Senior Editor
EMBO Molecular Medicine

*****
When preparing your revised manuscript, please refer to our guidelines: https://link.springer.com/journal/44321/submission-guidelines#cms-Revised-submissions. We perform an initial quality control of all revised manuscripts before re-review; failure to include requested items will delay the evaluation of your revision.

We require:

1) A .docx formatted version of the manuscript text (including legends for main figures, EV figures and tables). Please make sure that the changes are highlighted to be clearly visible.

2) Individual production quality figure files as .eps, .tif, .jpg (one file per figure). For guidance, download the 'Figure Guide PDF': https://media.springernature.com/original/springer-cms/rest/v1/content/27825798/data/v1.

3) A .docx formatted letter INCLUDING the reviewers' reports and your detailed point-by-point responses to their comments. As part of the EMBO Press transparent editorial process, the point-by-point response is part of the Review Process File (RPF), which will be published alongside your paper.

4) A complete author checklist, which you can download from our author guidelines. Please insert information in the checklist that is also reflected in the manuscript. The completed author checklist will also be part of the RPF.

5) Please note that all corresponding authors are required to supply an ORCID ID for their name upon submission of a revised manuscript.

6) It is mandatory to include a 'Data Availability' section after the Materials and Methods. Before submitting your revision, primary datasets produced in this study need to be deposited in an appropriate public database, and the accession numbers and database listed under 'Data Availability'. Please remember to provide a reviewer password if the datasets are not yet public.

In case you have no data that requires deposition in a public database, please state so in this section. Note that the Data Availability Section is restricted to new primary data that are part of this study.

7) For data quantification: please specify the name of the statistical test used to generate error bars and P values, the number (n) of independent experiments (specify technical or biological replicates) underlying each data point and the test used to calculate p-values in each figure legend. The figure legends should contain a basic description of n, P and the test applied. Graphs must include a description of the bars and the error bars (s.d., s.e.m.).

8) At EMBO Press we ask authors to provide source data for the main manuscript figures. You will receive a separate email with instructions for providing source data with your revised manuscript, including how to upload and organize the files.

9) Our journal encourages inclusion of *data citations in the reference list* to directly cite datasets that were re-used and obtained from public databases. Data citations in the article text are distinct from normal bibliographical citations and should directly link to the database records from which the data can be accessed. In the main text, data citations are formatted as follows: "Data ref: Smith et al, 2001" or "Data ref: NCBI Sequence Read Archive PRJNA342805, 2017". In the Reference list, data citations must be labeled with "[DATASET]". A data reference must provide the database name, accession number/identifiers and a resolvable link to the landing page from which the data can be accessed at the end of the reference.

10) We replaced Supplementary Information with Expanded View (EV) Figures and Tables that are collapsible/expandable online. A maximum of 5 EV Figures can be typeset. EV Figures should be cited as 'Figure EV1, Figure EV2" etc... in the text and their respective legends should be included in the main text after the legends of regular figures.

- For the figures that you do NOT wish to display as Expanded View figures, they should be bundled together with their legends in a single PDF file called *Appendix*, which should start with a short Table of Content. Appendix figures should be referred to in the main text as: "Appendix Figure S1, Appendix Figure S2" etc.

- Additional Tables/Datasets should be labeled and referred to as Table EV1, Dataset EV1, etc. Legends have to be provided in a separate tab in case of .xls files. Alternatively, the legend can be supplied as a separate text file (README) and zipped together with the Table/Dataset file.

11) The paper explained: EMBO Molecular Medicine articles are accompanied by a summary of the articles to emphasize the major findings in the paper and their medical implications for the non-specialist reader. Please provide a draft summary of your article highlighting

- the medical issue you are addressing,

- the results obtained and

- their clinical impact.

This may be edited to ensure that readers understand the significance and context of the research. Please refer to any of our published articles for an example.

12) Author contributions: You will be asked to provide CRediT (Contributor Role Taxonomy) terms in the submission system. These replace a narrative author contribution section in the manuscript.

13) A Conflict of Interest statement should be provided in the main text.

14) Please provide a 'Synopsis' to further enhance discoverability. Synopses are displayed on the journal webpage and are freely accessible to all readers. They include a short stand first (maximum of 300 characters, including space) as well as 2-5 one-sentences bullet points that summarizes the paper. Please write the bullet points to summarize the key NEW findings. They should be designed to be complementary to the abstract - i.e. not repeat the same text. We encourage inclusion of key acronyms and quantitative information (maximum of 30 words / bullet point). Please use the passive voice. Please attach these in a separate file or send them by email, we will incorporate them accordingly.

Please provide visual abstract to illustrate your article as a PNG file exactly 550 px wide x 300 px high.

15) All Materials and Methods need to be described in the main text using our 'Structured Methods' format. According to this format, the Methods section includes a Reagents and Tools Table (listing key reagents, experimental models, software and relevant equipment and including their sources and relevant identifiers) followed by a Methods and Protocols section describing the methods, ideally using a step-by-step protocol format. The aim is to facilitate adoption of the methodologies across labs.

Please download and fill our Reagents and Tools Table template (.docx), which you can find in our author guidelines: https://link.springer.com/journal/44320/submission-guidelines#structuredmethods.

EMBO Molecular Medicine has a "scooping protection" policy, whereby similar findings that are published by others during review or revision are not a criterion for rejection. Should you decide to submit a revised version, I do ask that you get in touch after three months if you have not completed it, to update us on the status.

***** Reviewer's comments *****

Referee #1 (Comments on Novelty/Model System for Author):

The technical quality, novelty and medical impact are high. The model systems are adequate for this kind of preclinical studies.

Referee #1 (Remarks for Author):

The present ms is about acid sphingomyelinase deficiency, a rare lysosomal storge disorder presenting a wide range of symptoms. Recently, an enzyme replacement therapy has been approved, but it is unclear whether and how ASMD patients with neurologic symptoms profit from this approach, as the enzyme cannot overcome the blood-brain barrier. This is all the more problematic as this group of ASMD patients becomes heavily disabled and shows the shortest life spans. Therefore, there is an urgent and unmet need for further understanding of neurologic disease mechanisms and new drug targets in the brain. The present work from one of the leading groups in the field fully meets this requirement. The authors corroborate the impact of dysmyelination as part of the neurodegenerative processes and they identify and validate a molecular target, CD44, whose continued overexpression hampers oligodendrocyte differentiation. The results represent a big step forward and open the doors to new investigations and drug development, possibly not only with respect to ASMD, but to other entries in the lysosomal disease spectrum. The paper presents a substantial amount of well founded data, but there are several weaknesses that need to be addressed. The authors should consider the following points:
- Throughout the ms, the authors should consider to use the term "ASMD with neurologic symptoms" rather than the somewhat "narrowing" term type A. Their results should be relevant for all ASMD patients presenting neurologic symptoms.
- Abstract, line 3: The authors should consider to remove "sphingomyelin-induced" as it is still not entirely known what induces dysfunction of specific neuronal cell types.
- Pg. 3, para 1: The phrase "To ameliorate the neurological progression" sounds ambiguous, it could be changed to "slow down or stop the neurological progression"
- Pg. 3, para 5, pg. 4, para 1; Fig. 1A, Fig. S1. The authors interpret the changes observed by electron microscopy as abberrant folding of myelin sheaths and they exclude defects in compaction based on similar g values in mutant and wildtype animals. However, the authors sampled only regions where myelin appeared compact (cf legend supp. fig. 1). Is it still possible that the in- and out-folding phenotype is a result of impaired compaction in specific regions along axons? The authors could strengthen their argument by including the important data shown in Fig. S1 in the main Fig. 1.
- Pg. 4, para 3: The description of oligodendrocyte development should be improved. The phrase "total differentiated lineage" is unclear and should be replaced by more precise term, for example "OL lineage marker" as OLIG2 labels the entire lineage from OPCs to mature OLs. Consequently, the statement "While the total number of OLs (Olig2+) remained" is incorrect, as Olig2+ labels the entire lineage (not just OLs). This should be corrected. The term "pre-OLs" is not defined and should be replaced by something like "pre-myelination OLs".
- Pg. 4, para 5; pg. 5, para 1-3: The description of transplantation and in vitro experiments and the interpretation of results should be improved and corrected. First, the authors should state in the main text, at which age oligo precursors were injected intravitreally. Second, the conclusion "that ASMko OLs possess a diminished intrinsic myelinating capacity" is not supported by the data and incongruent with their observation in the cerebellum: less mature CNPase-positive OLs. So, if the results are in line with the cerebellum and with what is shown in vitro, one would expect (and state) that the differentiation of transplanted A2B5 cells stalls at some stage leading to a lower density of mature CNPase+ cells in the optic nerve (with normal myelination capacity). A CNPase staining of post-transplantation optic nerves could help. Similarly, CNPAse staining could help to clarify, whether the ASM-deficient precursors generate less mature CNPase+ cells in vitro and whether sphingomyelin mimicks this effect.
Pg. 5 para 3 and 8 para 2: The authors should indicate (and justify) the concentration of sphingomyelin used in vitro. Did they observe dose-dependent effects?
Pg. 7, para 3: The experiments addressing defects on OPC morphology/polarisation raise several questions. First, the authors plated OPCs, but they claim to measure OL polarity. This is unjustified as there is no proof that these cells are OLs. Looking at Fig. 3, the cells look like OPCs. This should be revised. Second, the experiments performed here stand a bit isolated and the relevance of PDL/laminin as extracellular environment is unclear. Given the authors identification of CD44 it may be interesting

to repeat these morphology experiments with culture plates coated with hyaluronic acid and to test for morphologic changes in ASM-deficient OPCs in a model containing a natural ligand. These results could reinforce those shown in figures 4 and 5A,B.
- Pg 8, para 3: Again, the authors' use of the terms OL cultures and OPCs procee length is somewhat confusing. Did they use the same immunopanning protocol as described on pg. 7? In this case, these are primary cultures of OPCs, and not of OLs.
- Pg 9 para 2; pg 18, para 2: The behavioral data are very interesting and promising. A question is whether the authors have data repeated measures from individual mice allowing them to calculate tremor incidence and clasping duration at the start and end of treatment. This would allow for "per animal normalisation" and thus often helpful before-after analyses.
- Pg 9, para 2: The statement "... which escalated over the 14-week period" is a bit misleading and should be changed to something like "until 14 weeks-of-age".
- Pgs 9-10: Here, the discussion seems too focused on the authors results. As a suggestion, the authors could expand a bit and mention whether myelin defects occur in other lipid-related lysosomal disorders just to put their findings in a larger context.
- Pg. 11: The authors' finding of a disorganized hyaluronic acid matrix is very interesting. A question is which cells deposit this matrix normally, and why it is perturbed. Maybe the authors could discuss this briefly.
- Pgs 11-12: The authors should present the results shown in Appenix Fig. 3 in the appropriate section of the Results and in a main Figure.
- Pg. 14, para 5; Fig. 1 and legend: The authors should consider to indicate in a micrograph the internodal length in order to better understand how this was measured in MBP-stained sections.
- Figures (e.g. Fig. 4A, E; Fig. 5E): Correct y axes labels: whereever normalized values are shown, they do not indicate "arbitary units" (a.u.).
- All figures: The size of micrographs could be increased to render them more acessible. Moreover, single channel micrographs should be shown in greyscale rather than red or green to ensure visibility by color-blind readers. Colors of labels inside figures should be modified accordingly again to ensure visibility.

Referee #2 (Comments on Novelty/Model System for Author):

Tech quality score reflects lack of blinding. Medical impact reflects lack of human cell studies

Referee #2 (Remarks for Author):

This paper showing that the CNS effects of ASM deficiency result, at least in part, from a cell autonomous oligodendrocyte maturation defect secondary to increased CD44/HA interaction is well executed and interesting. It is potentially important as the authors show that a small molecule CD44 inhibitor Vb rescues some of the mouse ASM deficiency phenotype, a necessary step for experimental medicine approaches for this significant neurological disease.

Given this potential significance to an experimental medicine community, it's a surprise that the study has not been extended to use human cells, with engineered iPS or neural stem cell approaches to look at myelination in three-dimensional models. This would allow both analysis of the myelination phenotype and the effect of the small molecule inhibitor in a much more clinically relevant human setting.

Within the mouse work, I think that the range and power of the analysis should be extended in two ways. First, the quantitative immunofluorescence used in figure 5 is difficult. Better surely would be to use the more quantifiable measure of internodal length and see if this corrects with the inhibitor? Second, having identified extracellular matrix interactions as perturbed in the transcriptomics study, the authors then focus on CD44. It would be helpful to give context to this focus by presenting data on changes in other CNS extracellular matrix components and their receptors.

Finally, two technical points. i) Measurement of internodal length in density myelinated regions using MBP can be difficult. Could the authors replicate the findings using Caspr immunostaining (for paranodes) in combination with MBP to confirm that the entire myelin sheath is being imaged? ii) Observer blinding is essential in the analysis of myelin defects, especially in the EM morphology studies in Figure 1. The authors must clearly state in the methods how blinding was ensured in this and other experiments

****

Pre-decision cross-commenting

Referee #1:

I agree with referee 2 that data on human cells are important. However, their acquisition represents an entirely new and possibly lengthy study. In my opinion, the present mouse data merits publication if the authors respond to our comments.

Referee #2:

I agree that the human cell work would be a lengthy and new study. My comment was more to say that, in 2026, I think that studies written for an experimental medicine community should utilize human models and/or tissues as a first choice. The authors might look for human post mortem tissue that would allow the key findings to be validated, but I appreciate that the rarity of the disease might make that impossible. I'd be happy to support publication if my other points are addressed.

Referee #1

We thank this referee for the positive comments on our manuscript, acknowledging the technical quality, novelty and impact of the results. We are also very grateful for the identification of weaknesses, which have been addressed as follows:

1.Throughout the ms, the authors should consider to use the term "ASMD with neurologic symptoms" rather than the somewhat "narrowing" term type A. Their results should be relevant for all ASMD patients presenting neurologic symptoms.

We agree with the reviewer that the results obtained are relevant to all ASMD patients presenting neurological symptoms and not only to those with the most severe type A form of the disease. Following this suggestion, we now use the term *ASMD with neurologic symptoms* instead of *type A* where appropriate (lanes 37, 76). In addition, we have added a sentence at the end of the first paragraph of the Introduction broadening the classification of ASMD patients according to the severity of neurological involvement (lines 66-68).

2. Abstract, line 3: The authors should consider to remove "sphingomyelin-induced" as it is still not entirely known what induces dysfunction of specific neuronal cell types.

The reviewer is correct that the involvement of sphingomyelin in neuronal dysfunction has not yet been fully characterized. We have therefore removed “sphingomyelin-induced” from the text.

3. Pg. 3, para 1: The phrase "To ameliorate the neurological progression" sounds ambiguous, it could be changed to "slow down or stop the neurological progression"

The phrase has been changed accordingly (lines 63-64).

4. Pg. 3, para 5, pg. 4, para 1; Fig. 1A, Fig. S1. The authors interpret the changes observed by electron microscopy as aberrant folding of myelin sheaths and they exclude defects in compaction based on similar g values in mutant and wildtype animals. However, the authors sampled only regions where myelin appeared compact (cf legend supp. fig. 1). Is it still possible that the in- and out-folding phenotype is a result of impaired compaction in specific regions along axons?

The unaltered g-ratio observed in morphologically preserved myelin fibers excludes a global defect in myelin compaction. However, as the reviewer points out, we cannot rule out impaired compaction in specific regions presenting in- and out-folding. This point is now discussed in the manuscript (lines 120-122).

The authors could strengthen their argument by including the important data shown in Fig. S1 in the main Fig. 1.

In response to the reviewer’s suggestion, the g-ratio data have been moved from Appendix Fig. S1 to the Expanded View associated with Fig. 1, thereby integrating these data within the main manuscript while maintaining the clarity of the figure layout.

5. Pg. 4, para 3: The description of oligodendrocyte development should be improved. The phrase "total differentiated lineage" is unclear and should be replaced by more precise term, for example "OL lineage marker" as OLIG2 labels the entire lineage from OPCs to mature OLs. Consequently, the statement "While the total number of OLs (Olig2+) remained" is incorrect, as Olig2+ labels the entire lineage (not just OLs). This should be corrected. The term "pre-OLs" is not defined and should be replaced by something like "pre-myelination OLs".

We thank the reviewer for pointing out the imprecision in the description of the oligodendrocyte lineage. We have corrected the terminology according to the reviewer's suggestions (lines 157-160).

- Pg. 4, para 5; pg. 5, para 1-3: The description of transplantation and in vitro experiments and the interpretation of results should be improved and corrected.

- First, the authors should state in the main text, at which age oligo precursors were injected intravitreally.

OPCs were injected intravitreally into 2-month-old WT and ASMko mice. This information has now been clarified in the text (line 175).

- Second, the conclusion "that ASMko OLs possess a diminished intrinsic myelinating capacity" is not supported by the data and incongruent with their observation in the cerebellum: less mature CNPase-positive OLs. So, if the results are in line with the cerebellum and with what is shown in vitro, one would expect (and state) that the differentiation of transplanted A2B5 cells stalls at some stage leading to a lower density of mature CNPase+ cells in the optic nerve (with normal myelination capacity). A CNPase staining of post-transplantation optic nerves could help. Similarly, CNPase staining could help to clarify, whether the ASM-deficient precursors generate less mature CNPase+ cells in vitro and whether sphingomyelin mimicks this effect.

While we believe that the observed reduction in internodal length of the myelin produced after transplantation of A2B5 cells in retina is consistent with the myelination and oligodendroglial alterations identified in the cerebellum, the aim of the retinal transplantation experiment was not to compare oligodendroglial differentiation with that observed in the cerebellum, given the substantial differences between these systems and experimental conditions (physiological in the cerebellum versus transplanted in the retina). Instead, our objective was to determine whether intrinsic factors in ASMko oligodendroglia were sufficient to induce myelin alterations, or whether these alterations were secondary to ASM deficiency in the axons being wrapped. Similarly, the *in vitro* differentiation experiments were designed to assess morphological maturation in isolated oligodendroglial lineage cells rather than lineage distribution.

We agree that the previous wording of the manuscript may have been misleading, regarding the use of the term "diminished intrinsic myelinating capacity", which have been removed.

To further address the reviewer concern on the contribution of injected oligodendroglial lineage cells to retinal myelination, we have quantified MBP+ area relative to the number of Olig2+ cells within myelinated retinal regions as an indirect readout of myelin output per oligodendroglial lineage cell. In this experimental setting, where no overt degeneration is present, this approach provides an indirect estimate of the balance

between myelinating (MBP+) and total engrafted oligodendroglial lineage cells. We observed a trend towards reduced MBP/Olig2 ratio in retinas receiving ASMko cells, although this did not reach statistical significance (Figure 1 for this referee only). These data are consistent with the reduced internodal length observed after transplantation and support an intrinsic contribution of ASMko oligodendroglial lineage cells to the myelination phenotype.

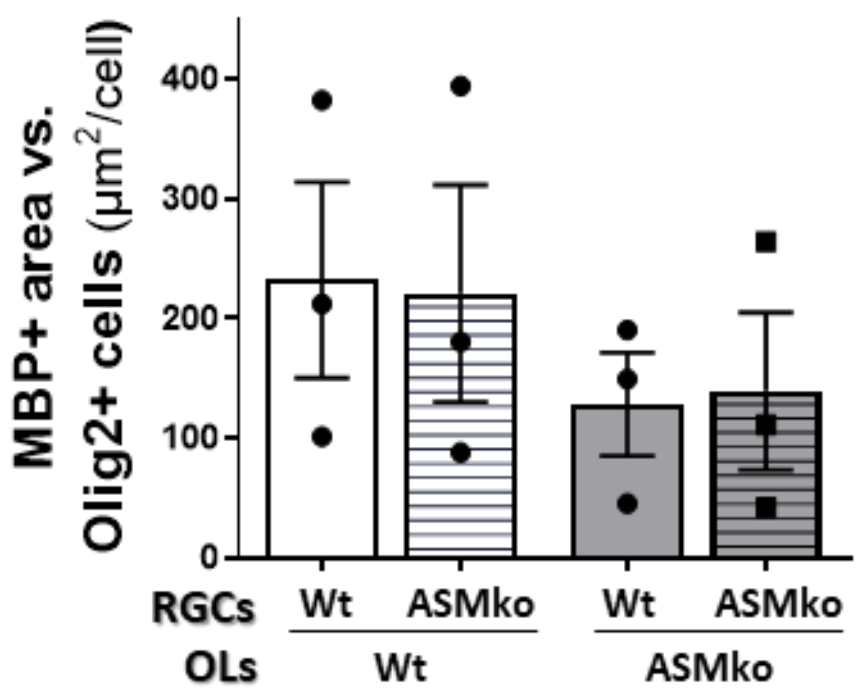

Figure 1 for this referee only. Graph shows mean ± SEM MBP+ area relative to the number of Olig2+ cells within myelinated retinal ganglion cells (RGC) regions of wt and ASMko mice after injection of wt or ASMko OLs (n=3)

- Pg. 5 para 3 and 8 para 2: The authors should indicate (and justify) the concentration of sphingomyelin used in vitro. Did they observe dose-dependent effects?

The experimental paradigm for sphingomyelin (SM) supplementation in WT OLs was adapted from previously published protocols in primary neuronal cultures (Arroyo et al., *EMBO Molecular Medicine*, 2014). The treatment regimen (20 μM on day 1 and 40 μM on day 3, maintained until day 4) was selected to avoid toxicity and to mimic the progressive accumulation of SM observed during differentiation under ASMko conditions. This approach resulted in a 65% increase in SM levels in SM-treated WT OLs, comparable to the increase observed in ASMko OLs. This information has now been included in lines 209–212 of the Results section and lines 718–720 of the Materials and Methods section.

- Pg. 7, para 3: The experiments addressing defects on OPC morphology/polarisation raise several questions.

-First, the authors plated OPCs, but they claim to measure OL polarity. This is unjustified as there is no proof that these cells are OLs. Looking at Fig. 3, the cells look like OPCs. This should be revised.

The cells shown in Fig. 3 are indeed OPCs. We apologize for the oversight, which has now been corrected.

- Second, the experiments performed here stand a bit isolated and the relevance of PDL/laminin as extracellular environment is unclear. Given the authors identification of CD44 it may be interesting to repeat these morphology experiments with culture plates coated with hyaluronic acid and to test for morphologic changes in ASM-deficient OPCs in a model containing a natural ligand. These results could reinforce those shown in figures 4 and 5A,B.

In the PDL/laminin experiments, our aim was to assess cell adhesion by examining spreading and polarization rather than differentiation, given the large number of

dysregulated genes associated with extracellular matrix interactions. This rationale has now been clarified in the manuscript (lines 304-305). We thank the reviewer for suggesting the use of hyaluronic acid (HA), which is indeed more relevant in the context of the CD44 alterations observed in mature OLs. In response to this suggestion, we performed HA-based functional assays using HA-coated substrates to directly assess CD44-dependent responses in a physiologically relevant extracellular matrix context.
To capture the full spectrum of HA-responsive behavior across the oligodendroglial lineage, we analyzed both freshly isolated OPCs obtained by MACS and cultures derived from MACS-purified OPCs differentiated in vitro for 4 days. Cells were replated onto HA-coated substrates and assessed for early spreading and polarization responses. No differences were detected between WT and ASMko OPCs. In contrast, cells derived from 4-day differentiated cultures exhibited altered HA-dependent cell spreading and morphological expansion under ASMko conditions compared with WT cells. These findings indicate that the effects of ASM deficiency on HA-responsive behavior are developmentally regulated and depend on the differentiation stage. These new experiments are described in lines 337-358 and are presented in the New Figures 4E, F and Appendix Fig. S4.

Pg 8, para 3: Again, the authors' use of the terms OL cultures and OPCs process length is somewhat confusing. Did they use the same immunopanning protocol as described on pg. 7?

In this case, these are primary OPC cultures isolated using MACS protocol and subsequently incubated in differentiation medium for four days. This has now been clarified in lines 305, 307.

Pg 9 para 2; pg 18, para 2: The behavioral data are very interesting and promising. A question is whether the authors have data repeated measures from individual mice allowing them to calculate tremor incidence and clasping duration at the start and end of treatment. This would allow for "per animal normalisation" and thus often helpful before-after analyses.

We thank the reviewer for this constructive suggestion and agree that longitudinal analyses at the individual level can provide additional insight into behavioral progression. In response, we revisited our dataset and incorporated new individual-based analyses where feasible. For hindlimb clasping, a before-and-after paired analysis was not informative because treatment started at postnatal day 15, when all animals are still asymptomatic and hindlimb clasping is absent. In contrast, daily monitoring of tremor throughout the treatment period enabled individual longitudinal analyses. Following the reviewer's suggestion, we have now included two additional analyses: (i) a time-to-onset (Kaplan–Meier) analysis evaluating the day of first tremor appearance in each individual mouse, and (ii) an analysis of tremor persistence, calculated as the percentage of days on which each animal exhibited tremor after symptom onset. These analyses further support the beneficial effect of Vb treatment, demonstrating a delayed onset of tremor and a trend toward reduced tremor persistence in treated ASMko mice. These new data are described in lines 414-424 and are presented in the new Appendix Fig. S5.

Pg 9, para 2: The statement "... which escalated over the 14-week period" is a bit misleading and should be changed to something like "until 14 weeks-of-age".

We apologize for the unclear wording. The text has been revised to clarify that the numbers in this paragraph (8 and 14 weeks) refer to treatment duration rather than the age of the mice (lines 410-411).

Pgs 9-10: Here, the discussion seems too focused on the authors results. As a suggestion, the authors could expand a bit and mention whether myelin defects occur in other lipid-related lysosomal disorders just to put their findings in a larger context.

We appreciate this suggestion to place our findings in a broader context. We now discuss myelin defects reported in other lipid-related lysosomal disorders in lines 440-451.

Pg. 11: The authors' finding of a disorganized hyaluronic acid matrix is very interesting. A question is which cells deposit this matrix normally, and why it is perturbed. Maybe the authors could discuss this briefly.

We thank the reviewer for this suggestion, which helped improve the discussion of our findings. We now refer to the current debate regarding the cells responsible for deposition of the hyaluronic acid matrix and its potential perturbation in ASMD in lines 517-541.

Pgs 11-12: The authors should present the results shown in Appendix Fig. 3 in the appropriate section of the Results and in a main Figure.

To address this query, we now describe the data on CD44-caveolin 2 colocalization in the Results section (lines 391-397) and include the quantification in the main Figure 5 (new panel C). To avoid overloading this figure and to preserve panel size, the immunofluorescence images are shown as expanded view (Fig. EV2).

Pg. 14, para 5; Fig. 1 and legend: The authors should consider to indicate in a micrograph the internodal length in order to better understand how this was measured in MBP-stained sections.

In the *in vivo* cerebellar analyses, internodal length was assessed in regions with lower myelin density (apical lobular areas), where individual fibers are clearly resolvable. Only clearly distinguishable isolated fibers were included in the analysis, and highly compact regions were excluded. This is now better explained in the materials and methods section (lines 750-752). In addition, we have included an inset with a representative internode in Figure 1C (P10) and explained it in the figure legend (lines 1169-1172).

Figures (e.g. Fig. 4A, E; Fig. 5E): Correct y axes labels: whereever normalized values are shown, they do not indicate "arbitary units" (a.u.).

We have corrected the y-axis labels accordingly in Figs. 4A,E and 5E, and also in Figs. 2 and 3.

All figures: The size of micrographs could be increased to render them more acessible. Moreover, single channel micrographs should be shown in greyscale rather than red or green to ensure visibility by color-blind readers. Colors of labels inside figures should be modified accordingly again to ensure visibility

We assembled the figures in the original version with the aim of maximizing micrograph size within the available space. To accommodate the reviewer's request as much as possible, we have increased the size of the micrographs in Figure 1B,C,G. However, we kindly ask the reviewer to allow us to retain the current layout for the remaining figures. Regarding the colors, we thank the reviewer for the suggestion. We have retained the single-channel images in their original colors, as color-blind readers can still distinguish intensity differences in the red and green channels. However, we modified the merged images to use color combinations that are more easily distinguishable for color-blind readers (Figure 3, Figure 4, Figure EV2, Appendix Fig S2).

Referee #2

We thank the referee for considering our work well executed and interesting, and for the constructive comments that helped improve the manuscript. These comments have been addressed as follows:

Given this potential significance to an experimental medicine community, it's a surprise that the study has not been extended to use human cells, with engineered iPS or neural stem cell approaches to look at myelination in three-dimensional models. This would allow both analysis of the myelination phenotype and the effect of the small molecule inhibitor in a much more clinically relevant human setting.

We agree with Reviewer 2 that data obtained from human samples are important, and we appreciate the thoughtful consideration from both reviewers acknowledging the challenges associated with obtaining such material for this study. To address this concern as thoroughly as possible, we obtained access to postmortem human tissue and performed immunofluorescence analyses comparing myelin (MBP+) in the brain of an ASMD type A patient and an age-matched control child. Unfortunately, our attempts to immunolabel oligodendroglia at different differentiation stages were unsuccessful, as antibodies against human NG2 and CNPase did not yield reliable staining. Nevertheless, MBP staining revealed a reduced MBP+ area, increased myelin debris, and a higher number of MBP+ contact-forming OLs in the ASMD type A patient compared with the control, whereas the total number of oligodendroglial cells, identified by SOX10 staining, was not significantly altered (Appendix Figure S1). Although the rarity of the disease precluded the analysis of additional samples, we believe that these findings, which are described in lines 142-150, support the relevance of our observations in the human setting.

Within the mouse work, I think that the range and power of the analysis should be extended in two ways.

First, the quantitative immunofluorescence used in figure 5 is difficult. Better surely would be to use the more quantifiable measure of internodal length and see if this corrects with the inhibitor?

Quantitative immunofluorescence was used in this figure to assess Vb-induced improvements in myelin area, myelin debris, NG2+ area, and CNPase+ area, since these are all parameters that were found altered in ASMko versus WT cerebellum (Figure 1). Following the reviewer's suggestion, we have now measured internodal length as well (see Figure 2 for this referee only). We did not observe significant differences in the internodal length in ASMko mice treated with vehicle or with the inhibitor verbascoside. It is possible that this alteration arises very early and is therefore more difficult to reverse with treatment initiated at postnatal day 15.

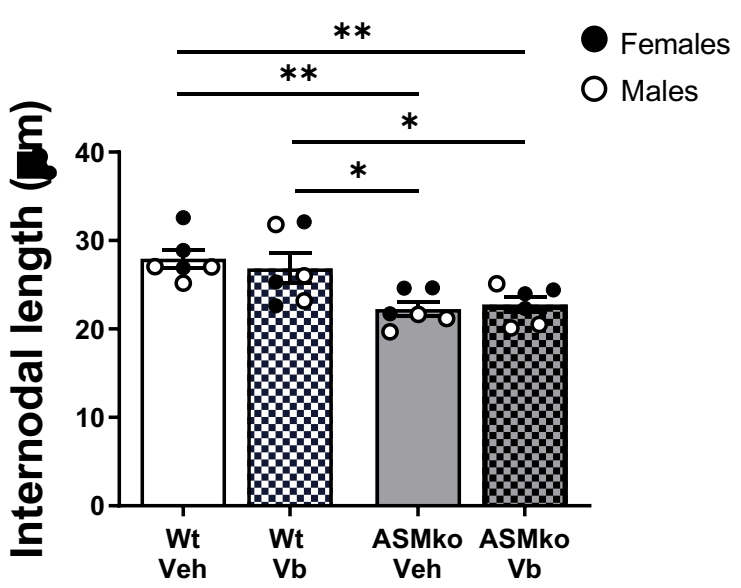

Figure 2 for this referee only. Graph shows mean ± SEM internodal length in µm in the cerebellum of wt and ASMko mice treated with vehicle or with Verbascoside (n=6). Black and white dots denote females and males, respectively; sex ratios were balanced. Statistics: Ordinary one-way ANOVA followed by Sidak's post hoc comparisons test: $p_{\text{ASMko Veh vs. Wt Veh}} = 0.002$, $p_{\text{Wt Vb vs. Wt Veh}} = 0.510$, $p_{\text{ASMko Vb vs. ASMko Veh}} = 0.767$, $p_{\text{ASMko Vb vs. Wt Veh}} = 0.005$, $p_{\text{ASMko Veh vs. Wt Vb}} < 0.011$, $p_{\text{ASMko Vb vs. Wt Vb}} = 0.021$.

Second, having identified extracellular matrix interactions as perturbed in the transcriptomics study, the authors then focus on CD44. It would be helpful to give context to this focus by presenting data on changes in other CNS extracellular matrix components and their receptors.

We thank the reviewer for this suggestion, which helped provide broader context for our focus on CD44. In the revised manuscript, we now report and discuss coordinated changes in multiple extracellular matrix components (including laminins and collagens) as well as cell-ECM receptors such as integrins, supporting a generalized disruption of OL-matrix interactions in ASMko cells (lines 503-508).

Finally, two technical points:

i) Measurement of internodal length in density myelinated regions using MBP can be difficult. Could the authors replicate the findings using Caspr immunostaining (for paranodes) in combination with MBP to confirm that the entire myelin sheath is being imaged?

We agree that internodal length measurements based solely on MBP staining can be challenging in densely myelinated regions. In our study, we addressed this concern by applying context-specific validation strategies.

For the in vivo cerebellar analyses, internodal length was measured in regions with lower myelin density (apical lobular areas), where individual fibers can be clearly resolved. Only well-isolated and clearly distinguishable fibers were included in the analysis, whereas highly compact regions were excluded. This is now explained in lines 750-752. In addition, a representative internode is shown as an inset in panel C (P10) of Figure 1 and described in the figure legend (lines 1169-1172).

In the OPC transplantation experiments onto retinas, Caspr staining was additionally used because myelin organization can be more variable during de novo sheath formation. Although Caspr staining had already been performed, it was not included in the original figure because the low magnification required to visualize large retinal areas did not permit adequate visualization of paranodal structures. We have now included higher-magnification images showing Caspr-positive paranodes flanking MBP-positive internodes, thereby confirming that complete internodal segments were analyzed. These data are now included in the new Appendix Figure S2 and are described on lines 788-789.

ii) Observer blinding is essential in the analysis of myelin defects, especially in the EM morphology studies in Figure 1. The authors must clearly state in the methods how blinding was ensured in this and other experiments.

We now clearly state in the Methods section (line 904-906) how blinding was ensured across all experiments.

20th Jul 2026

Dear Dr. Ledesma,

Thank you for the submission of your revised manuscript to EMBO Molecular Medicine. We have now received the reports from the two referees who re-evaluated your study. As you will see from the comments below, both referees are generally satisfied with the revisions you have made. Referee #2 has, however, raised a few remaining comments, and we therefore ask that you revise your manuscript accordingly and provide a point-by-point response to the comments.

On a more editorial level:

1. Please upload the EV figures as separate files, with one figure per file.

2. Please ensure that the funding information listed in the manuscript is consistent with the information entered in the submission system. The following funding sources appear to be missing from the submission system (and there may be others): MICIU/AEI/10.13039/501100011033; LeukoReMy; Ramón Areces Foundation.

3. Please revise the Paper Explained section to include separate subsections entitled Problem, Results, and Impact, in line with the journal format. Please refer to published papers for examples.

4. "Declaration of competing interest" should be renamed to "Disclosure and Competing Interests Statement" .

5. When submitting your revised manuscript, please remove the Reagents and Tools Table from the Methods section of the manuscript but upload it as a separate file choosing the file type "Reagent Table".

6. Callouts: Please update the callout for Fig. S4B to Appendix Fig. S4B, and add the missing callout for Appendix Fig. S5. Please also ensure that the term "Supplementary" is not used.

7. In the Methods, for human studies, include a statement that informed consent was obtained from all subjects and that the experiments conformed to the principles set out in the WMA Declaration of Helsinki and the Department of Health and Human Services Belmont Report.

8. Appendix: Please add page numbers to the items listed in the Table of Contents. In addition, please rename the title "Appendix Supplementary Figures" to simply "Appendix."

9. The visual abstract should be provided as a separate PNG file (rather than a PDF), with dimensions of exactly 550 px (width) × 300 px (height) to ensure optimal display on the website.

10. The Data Availability section should be placed before the Acknowledgments. Please also note that the following statements should not be used: "Data will be made available upon request," "The source data of this paper are collected in the following database record BioStudies," and "Expanded View data and supplementary information for this article are available online."

11. Please provide a completed Source Data Checklist (which was sent to you on April 1) and ensure that the information is consistent with the provided source data files.

12. Please address the following issues in figure legends:
-Please define the annotated p values ****/***/**/* as well as provide the exact p-values for the same in the legend of figure S3 B as appropriate.
- Please indicate what */ **/ ***/ **** represents; if this represents p value(s), please specify the exact p value in the legend(s) of figure(s) 2C, D, G, H, I; 4F, 5A, B, C, F, G, H, J, K; S1 A
- Please indicate the statistical test used for data analysis in the legends of figures S3 A, B
- Please note that the error bars are not defined in the legends of figures EV1 B, S3 B
- Please note that scale bar and its definition are missing for figure1A.

I look forward to reading a new revised version of your manuscript as soon as possible.

Sincerely,
Jingyi

Jingyi Hou
Senior Editor
EMBO Molecular Medicine

*** Instructions to submit your revised manuscript ***

*** PLEASE NOTE *** As part of the EMBO Publications transparent editorial process initiative (see our Editorial at https://www.embopress.org/doi/pdf/10.1002/emmm.201000094), EMBO Molecular Medicine will publish online a Review Process File to accompany accepted manuscripts.

In the event of acceptance, this file will be published in conjunction with your paper and will include the anonymous referee reports, your point-by-point response and all pertinent correspondence relating to the manuscript. If you do NOT want this file to be published, please inform the editorial office at contact@embomolmed.org.

*****

When preparing your revised manuscript, please refer to our guidelines: https://link.springer.com/journal/44321/submission-guidelines#cms-Revised-submissions. We perform an initial quality control of all revised manuscripts before re-review; failure to include requested items will delay the evaluation of your revision.

***** Reviewer's comments *****

Referee #1 (Comments on Novelty/Model System for Author):

The authors report novel results that are of high interest for the lysosomal disease communities.

Referee #1 (Remarks for Author):

The authors have addressed all comments in a highly satisfactory manner. The present version of the manuscript is suitable for publication.

Referee #2 (Remarks for Author):

The careful attention to the reviewer comments is welcome, and the manuscript is sufficiently improved. The inclusion of human data, albeit with appropriate caveats, adds to the power and interest of the study. My only comment now would be around the failure of vb to rescue the internode length defect. If this is due to the time of administration, as suggested in the letter, why are the other metrics of myelination corrected? Its interesting, and an area for further study - and so I think this data should be included in the MS with text in the final para and a figure in the supplementary data. I would suggest that there is more than one ECM related process at work here, and that CD44 may regulate differentiation but not sheath geometry

POINT-BY-POINT AMNSWER TO REVIEWER´S COMMENTS

Referee #1

We thank the reviewer for recognizing the novelty and significance of our findings for the lysosomal disease field and for considering the manuscript suitable for publication.

Referee #2

We thank the reviewer for considering that the manuscript has been substantially improved and for acknowledging the inclusion of the human data. To address the remaining comment regarding the inability of verbascoside to rescue the internode length defect, we have now included these data as **Appendix Fig. S6** and discussed them in **lines 406–413**.

We agree with the reviewer that more than one extracellular matrix (ECM)-related mechanism may contribute to the phenotype observed in ASMko mice, and that CD44 may regulate oligodendrocyte differentiation without affecting myelin sheath geometry. This observation opens interesting avenues for future investigation.

29th Jul 2026

Dear Dr. Ledesma,

We are pleased to inform you that your manuscript is accepted for publication and is now being sent to our publisher to be included in the next available issue of EMBO Molecular Medicine.

Your manuscript will be processed for publication by EMBO Press. It will be copy edited and you will receive page proofs prior to publication. Please note that you will be contacted by Springer Nature Author Services to complete licensing and payment information.

You may qualify for financial assistance for your publication charges - either via a Springer Nature fully open access agreement or an EMBO initiative. Check your eligibility: https://link.springer.com/journal/44321/how-to-publish-with-us

Should you be planning a Press Release on your article, please notify the production team via this form in order to coordinate publication and release dates: https://forms.cloud.microsoft/e/eH4A8JVP6g

If you have any questions, please do not hesitate to contact the Editorial Office. Thank you for your contribution to EMBO Molecular Medicine.

Yours sincerely,
Jingyi

Jingyi Hou
Senior Editor
EMBO Molecular Medicine

>>> Please note that it is EMBO Molecular Medicine policy for the transcript of the editorial process (containing referee reports and your response letter) to be published as an online supplement to each paper. If you do NOT want this, you will need to inform the Editorial Office via email immediately. More information is available here: https://link.springer.com/partners/embo-press/editorial-policies#Peer%20review

EMBO Press Author Checklist

Corresponding Author Name: María Dolores Ledesma and Marta Guerrero-Valero	
Journal Submitted to: EMBO Molecular Medicine	
Manuscript Number: EMM-2026-23550	

USEFUL LINKS FOR COMPLETING THIS FORM
The EMBO Journal - Author Guidelines
EMBO Reports - Author Guidelines
Molecular Systems Biology - Author Guidelines
EMBO Molecular Medicine - Author Guidelines

Reporting Checklist for Life Science Articles (updated January
This checklist is adapted from Materials Design Analysis Reporting (MDAR) Checklist for Authors. MDAR establishes a minimum set of requirements in transparent reporting in the life sciences (see Statement of Task: 10.31222/osf.io/9sm4x). Please follow the journal's guidelines in preparing your
Please note that a copy of this checklist will be published alongside your article.

Abridged guidelines for figures
1. Data
The data shown in figures should satisfy the following conditions:
- the data were obtained and processed according to the field's best practice and are presented to reflect the results of the experiments in an accurate and unbiased manner.
- ideally, figure panels should include only measurements that are directly comparable to each other and obtained with the same assay.
- plots include clearly labeled error bars for independent experiments and sample sizes. Unless justified, error bars should not be shown for technical
- if n<5, the individual data points from each experiment should be plotted. Any statistical test employed should be justified.
- Source Data should be included to report the data underlying figures according to the guidelines set out in the authorship guidelines on Data

2. Captions
Each figure caption should contain the following information, for each panel where they are relevant:
- a specification of the experimental system investigated (eg cell line, species name).
- the assay(s) and method(s) used to carry out the reported observations and measurements.
- an explicit mention of the biological and chemical entity(ies) that are being measured.
- an explicit mention of the biological and chemical entity(ies) that are altered/varied/perturbed in a controlled manner.
- the exact sample size (n) for each experimental group/condition, given as a number, not a range;
- a description of the sample collection allowing the reader to understand whether the samples represent technical or biological replicates (including how many animals, litters, cultures, etc.).
- a statement of how many times the experiment shown was independently replicated in the laboratory.
- definitions of statistical methods and measures:
  - common tests, such as t-test (please specify whether paired vs. unpaired), simple χ2 tests, Wilcoxon and Mann-Whitney tests, can be unambiguously identified by name only, but more complex techniques should be described in the methods section;
  - are tests one-sided or two-sided?
  - are there adjustments for multiple comparisons?
  - exact statistical test results, e.g., P values = x but not P values < x;
  - definition of 'center values' as median or average;
  - definition of error bars as s.d. or s.e.m.

Please complete ALL of the questions below.
Select "Not Applicable" only when the requested information is not relevant for your study.

Materials

Newly Created Materials	Information included in the manuscript?	In which section is the information available? (Reagents and Tools Table, Materials and Methods, Figures, Data Availability Section)
New materials and reagents need to be available; do any restrictions apply?	Not Applicable	

Antibodies	Information included in the manuscript?	In which section is the information available? (Reagents and Tools Table, Materials and Methods, Figures, Data Availability Section)
For **antibodies** provide the following information:<br>- Commercial antibodies: RRID (if possible) or supplier name, catalogue number and or/clone number<br>- Non-commercial: RRID or citation	Yes	Reagents and Tools Table

DNA and RNA sequences	Information included in the manuscript?	In which section is the information available? (Reagents and Tools Table, Materials and Methods, Figures, Data Availability Section)
**Short novel DNA or RNA including primers, probes**: provide the sequences.	Not Applicable	

Cell materials	Information included in the manuscript?	In which section is the information available? (Reagents and Tools Table, Materials and Methods, Figures, Data Availability Section)
**Cell lines:** Provide species information, strain. Provide accession number in repository **OR** supplier name, catalog number, clone number, and/**OR** RRID.	Not Applicable	
**Primary cultures:** Provide species, strain, sex of origin, genetic modification status.	Yes	Materials and Methods
Report if the cell lines were recently **authenticated** (e.g., by STR profiling) and tested for mycoplasma contamination.	Not Applicable	

Experimental animals	Information included in the manuscript?	In which section is the information available? (Reagents and Tools Table, Materials and Methods, Figures, Data Availability Section)
**Laboratory animals or Model organisms:** Provide species, strain, sex, age, genetic modification status. Provide accession number in repository **OR** supplier name, catalog number, clone number, **OR** RRID.	Yes	Reagents and Tools Table, Materials and Methods and Figures
**Animal observed in or captured from the field:** Provide species, sex, and age where possible.	Not Applicable	
Please detail **housing and husbandry conditions**.	Yes	Materials and Methods

Plants and microbes	Information included in the manuscript?	In which section is the information available? (Reagents and Tools Table, Materials and Methods, Figures, Data Availability Section)
**Plants:** provide species and strain, ecotype and cultivar where relevant, unique accession number if available, and source (including location for collected wild specimens).	Not Applicable	
**Microbes:** provide species and strain, unique accession number if available, and source.	Not Applicable	

Human research participants	Information included in the manuscript?	In which section is the information available? (Reagents and Tools Table, Materials and Methods, Figures, Data Availability Section)
If collected and within the bounds of privacy constraints report on age, sex and gender or ethnicity for all study participants.	Not Applicable	

Core facilities	Information included in the manuscript?	In which section is the information available? (Reagents and Tools Table, Materials and Methods, Figures, Data Availability Section)
If your work benefited from core facilities, was their service mentioned in the acknowledgments section?	Yes	

Design

Study protocol	Information included in the manuscript?	In which section is the information available? (Reagents and Tools Table, Materials and Methods, Figures, Data Availability Section)
If study protocol has been **pre-registered, provide DOI in the manuscript**. For clinical trials, provide the trial registration number **OR** cite DOI.	Not Applicable	
Report the **clinical trial registration number** (at ClinicalTrials.gov or equivalent), where applicable.	Not Applicable	

Laboratory protocol	Information included in the manuscript?	In which section is the information available? (Reagents and Tools Table, Materials and Methods, Figures, Data Availability Section)
Provide DOI OR other citation details if **external detailed step-by-step protocols** are available.	Not Applicable	

Experimental study design and statistics	Information included in the manuscript?	In which section is the information available? (Reagents and Tools Table, Materials and Methods, Figures, Data Availability Section)
Include a statement about **sample size** estimate even if no statistical methods were used.	Yes	Materials and Methods
Were any steps taken to minimize the effects of subjective bias when allocating animals/samples to treatment (e.g. **randomization procedure**)? If yes, have they been described?	Yes	Materials and Methods
Include a statement about **blinding** even if no blinding was done.	Yes	Materials and Methods
Describe **inclusion/exclusion criteria** if samples or animals were excluded from the analysis. Were the criteria pre-established? If sample or data points were omitted from analysis, report if this was due to attrition or intentional exclusion and provide justification.	Not Applicable	
For every figure, are **statistical tests** justified as appropriate? Do the data meet the assumptions of the tests (e.g., normal distribution)? Describe any methods used to assess it. Is there an estimate of variation within each group of data? Is the variance similar between the groups that are being statistically compared?	Yes	Materials and Methods

Sample definition and in-laboratory replication	Information included in the manuscript?	In which section is the information available? (Reagents and Tools Table, Materials and Methods, Figures, Data Availability Section)
In the figure legends: state number of times the experiment was **replicated** in laboratory.	Yes	Figure legends
In the figure legends: define whether data describe **technical or biological replicates**.	Yes	Figure legends

Ethics

Ethics	Information included in the manuscript?	In which section is the information available? (Reagents and Tools Table, Materials and Methods, Figures, Data Availability Section)
Studies involving **human participants**: State details of **authority granting ethics approval** (IRB or equivalent committee(s), provide reference number for approval.	Not Applicable	
Studies involving **human participants**: Include a statement confirming that **informed consent** was obtained from all subjects and that the experiments conformed to the principles set out in the WMA Declaration of Helsinki and the Department of Health and Human Services Belmont Report.	Not Applicable	
Studies involving **human participants:** For publication of **patient photos**, include a statement confirming that consent to publish was obtained.	Not Applicable	
Studies involving experimental **animals**: State details of **authority granting ethics approval** (IRB or equivalent committee(s), provide reference number for approval. Include a statement of compliance with ethical regulations.	Yes	Materials and Methods
Studies involving **specimen and field samples:** State if relevant **permits** obtained, provide details of authority approving study; if none were required, explain why.	Not Applicable	

Dual Use Research of Concern (DURC)	Information included in the manuscript?	In which section is the information available? (Reagents and Tools Table, Materials and Methods, Figures, Data Availability Section)
Could your study fall under dual use research restrictions? Please check biosecurity documents and list of **select agents and toxins** (CDC): https://www.selectagents.gov/sat/list.htm	Not Applicable	
If you used a select agent, is the security level of the lab appropriate and reported in the manuscript?	Not Applicable	
If a study is subject to dual use research of concern regulations, is the name of the **authority granting approval and reference number** for the regulatory approval provided in the manuscript?	Not Applicable	

Reporting

The MDAR framework recommends adoption of discipline-specific guidelines, established and endorsed through community initiatives. Journals have their own policy about requiring specific guidelines and recommendations to complement MDAR.

Adherence to community standards	Information included in the manuscript?	In which section is the information available? (Reagents and Tools Table, Materials and Methods, Figures, Data Availability Section)
State if relevant guidelines or checklists (e.g., **ICMJE, MIBBI, ARRIVE, PRISMA**) have been followed or provided.	Yes	Materials and Methods
For **tumor marker prognostic studies,** we recommend that you follow the **REMARK** reporting guidelines (see link list at top right). See author guidelines, under 'Reporting Guidelines'. Please confirm you have followed these guidelines.	Not Applicable	
For **phase II and III randomized controlled trials**, please refer to the **CONSORT** flow diagram (see link list at top right) and submit the CONSORT checklist (see link list at top right) with your submission. See author guidelines, under 'Reporting Guidelines'. Please confirm you have submitted this list.	Not Applicable	

Data Availability

Data availability	Information included in the manuscript?	In which section is the information available? (Reagents and Tools Table, Materials and Methods, Figures, Data Availability Section)
Have **primary datasets** been deposited according to the journal's guidelines (see 'Data Deposition' section) and the respective accession numbers provided in the Data Availability Section?	Yes	Data Availability Section
Were **human clinical and genomic datasets** deposited in a public access-controlled repository in accordance to ethical obligations to the patients and to the applicable consent agreement?	Not Applicable	
Are **computational models** that are central and integral to a study available without restrictions in a machine-readable form? Were the relevant accession numbers or links provided?	Not Applicable	
If publicly available data were reused, provide the respective **data citations in the reference list**.	Not Applicable	
