# Supplementary material for: Targeting CD44 reverses sphingomyelin-induced oligodendrocyte maturation arrest in acid sphingomyelinase deficiency
Source: EMBO Mol Med. 2026 Aug 12;18(9):3767–89. doi: 10.1038/s44321-026-00503-8 (PMC13562706; doi:10.1038/s44321-026-00503-8)
Supplement: Supplementary file 3 [file 44321_2026_503_MOESM2_ESM.pdf]

APPENDIX

Targeting CD44 reverses sphingomyelin-induced oligodendrocyte maturation arrest in acid sphingomyelinase deficiency

Marta Guerrero-Valero[1#*], Elena Melgarejo#[1], Miguel A. Marchena[2], Beatriz Fernández-Gómez[2], Jaime Mulero-Franco[4], Fernando de Castro[2], Edward H. Schuchman[3], María Dolores Ledesma[1*]

[1] Centro Biología Molecular Severo Ochoa (CSIC-UAM), 28049 Madrid, Spain

[2] Centro de Neurociencias Cajal/Instituto Cajal-CSIC, Madrid, Spain

[3] Department of Genetics & Genomic Sciences, Icahn School of Medicine at Mount Sinai, New York, NY, USA.

[4]Universidad de Castilla-La Mancha, Ciudad Real, Castilla-La Mancha, Spain.

*Corresponding authors: mguerr25@ucm.es, dledesma@cbm.csic.es

# equal contribution

Table of content

Appendix Figure S1. Myelin and oligodendroglial alterations in cerebellar tissue from a patient with ASMD type A (page 2)

Appendix Figure S2. Internodal segment definition in the retinal transplantation model (page 3)

Appendix Figure S3. Quality control of OL isolation protocol (page 4)

Appendix Figure S4. Polarity index analysis of WT and ASMko OPCs during early adhesion to hyaluronic acid (page 5)

Appendix Figure S5. Individual-level analyses of tremor onset and persistence in ASMko mice (page 6)

Appendix Figure S6. Quantification of internodal length in WT and ASMko mice treated or not with Vb (page 7)

APPENDIX FIGURE S1

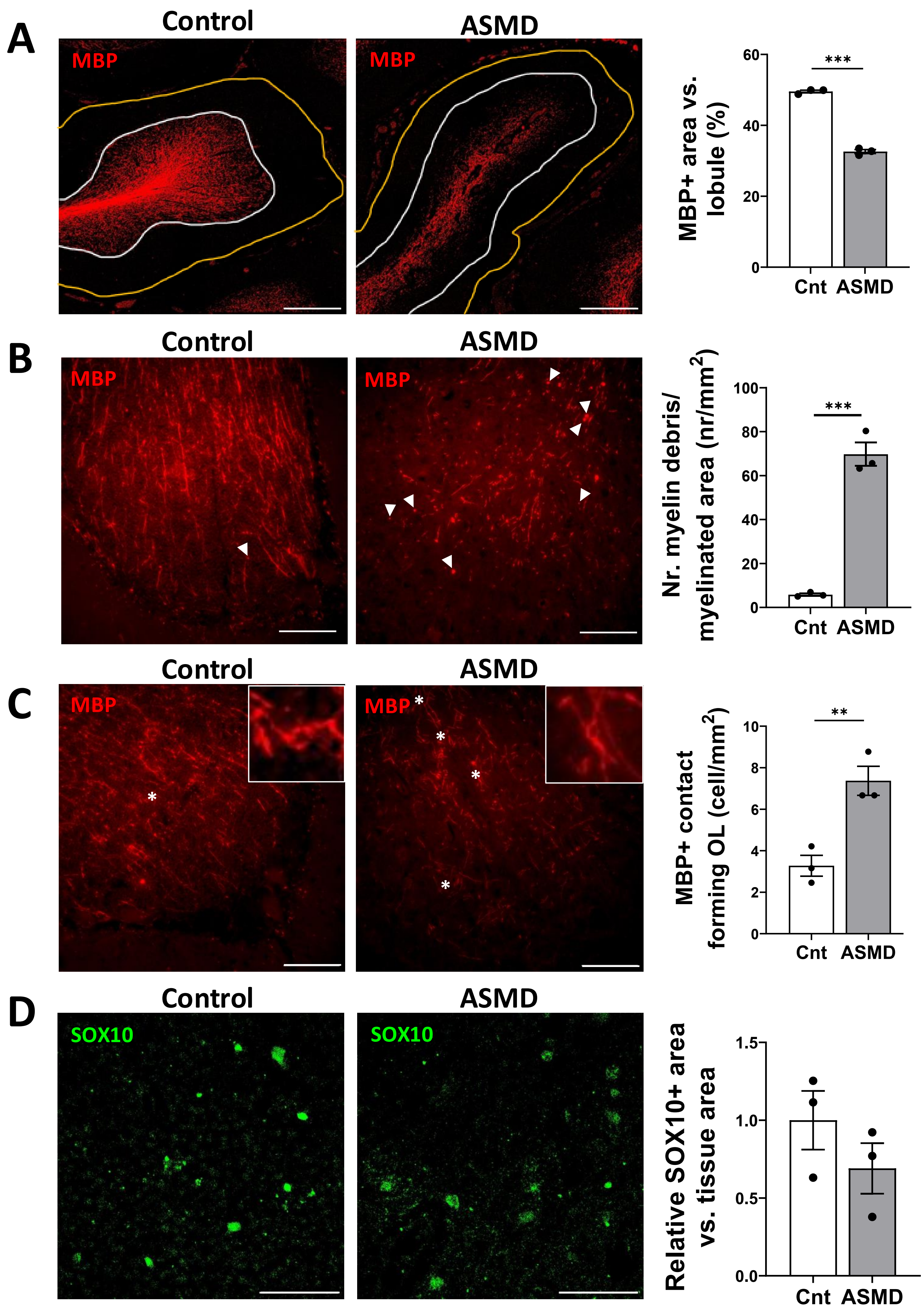

Appendix Fig. S1. Myelin and oligodendroglial alterations in cerebellar tissue from a patient with ASMD type A.
A) Representative low-magnification immunofluorescence images of MBP in cerebellar lobules from a patient with ASMD type A and an age-matched control child. The cerebellar lobule area is outlined by a yellow line, while the MBP+ area is delimited by a white line. Data show mean ± SEM percentage of the cerebellar area occupied by MBP. Statistical analysis was performed using two-tailed t-test (n = 3 brain slices from a single patient; technical replicates; $p < 0.0001$). Scale bar: 500 µm.

B) Representative high-magnification immunofluorescence images of MBP within the apical regions of the cerebellar lobules from a patient with ASMD type A and an age-matched control child. Myelin debris is indicated by arrowheads, with magnified views shown in insets. Data show mean ± SEM number of myelin debris per myelinated area. Statistical analysis was performed using two-tailed t-test (n = 3 brain slices from a single patient; technical replicates; $p = 0.0003$). Scale bar: 200 µm.

C) Representative immunofluorescence images of MBP in cerebellar lobules from a patient with ASMD type A and an age-matched control child. Asterisks mark MBP+ contact-forming OLs; the upper-right insets show magnified views. Data show mean ± SEM number of MBP+ cells at the stage of axonal contact and initial wrapping per area of the cerebellar lobules. Statistical analysis was performed using two-tailed t-test (n = 3 brain slices from a single patient; technical replicates; $p = 0.009$). Scale bar: 200 µm.

D) Representative high-magnification immunofluorescence images of SOX10 within the apical regions of the cerebellar lobules from a patient with ASMD type A and an age-matched control child. Data show mean ± SEM SOX10+ area as percentage of the total lobule area. Statistical analysis was performed using two-tailed t-test (n = 3 brain slices from a single patient; technical replicates; $p = 0.281$). Scale bar: 40 µm.

Asterisks in the graphs of all panels denote significance levels: $^{**}p < 0.01$; $^{***}p < 0.001$.

APPENDIX FIGURE S2

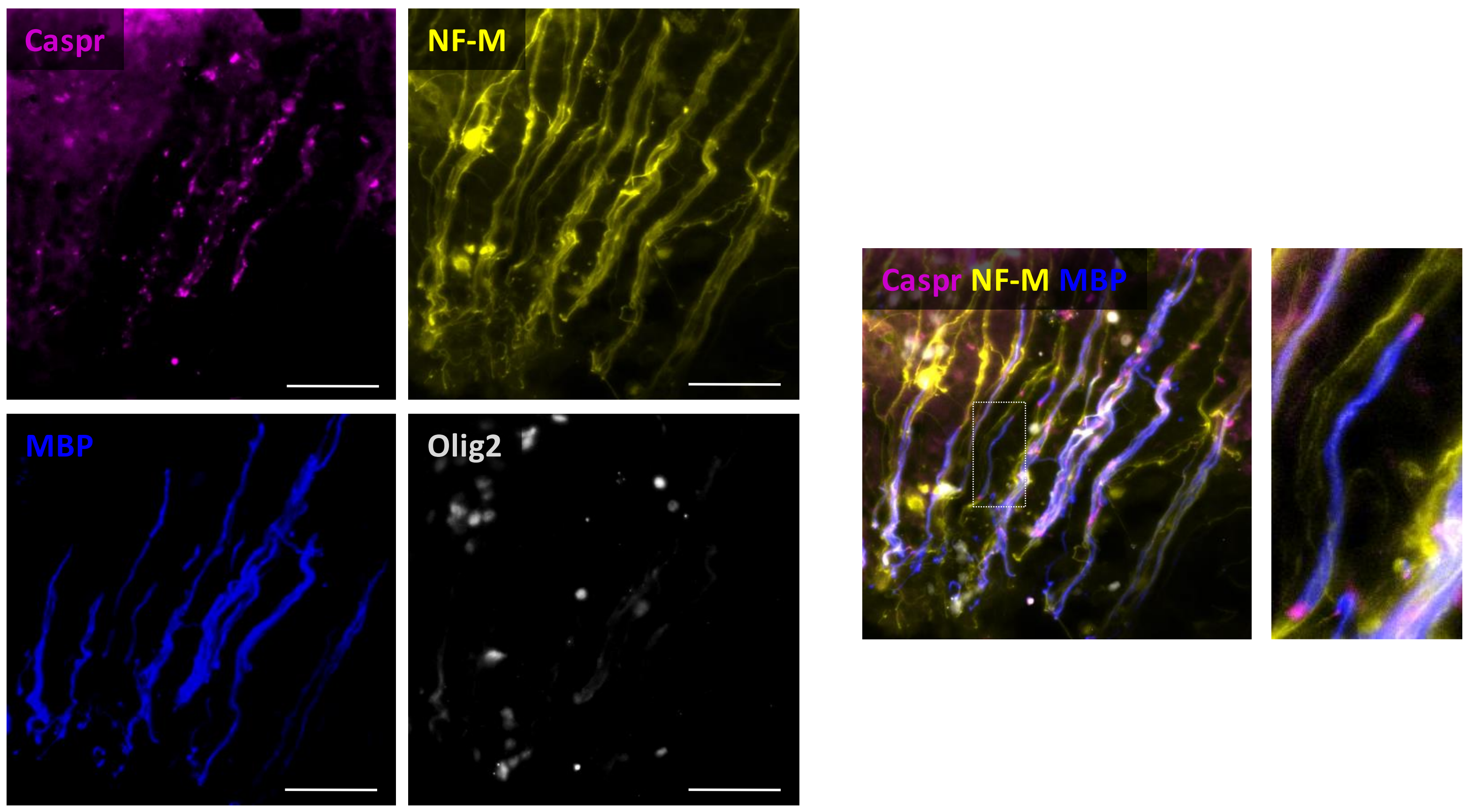

Appendix Fig. S2. Internodal segment definition in the retinal transplantation model
Representative immunofluorescence images showing myelinated internodal segments in the retinal transplantation model. Single channels are shown on the left: the paranode marker Caspr and the axonal market NF-M (top row), and MBP and Olig2 (bottom row). Merged images on the right illustrate myelinated internodes, with MBP+ segments along NF-M+ axons flanked by Caspr+ paranodal regions. The inset highlights a representative internode with clear paranodal Caspr labeling at both ends of the MBP+ segment. Scale bar: 50 µm.

APPENDIX FIGURE S3

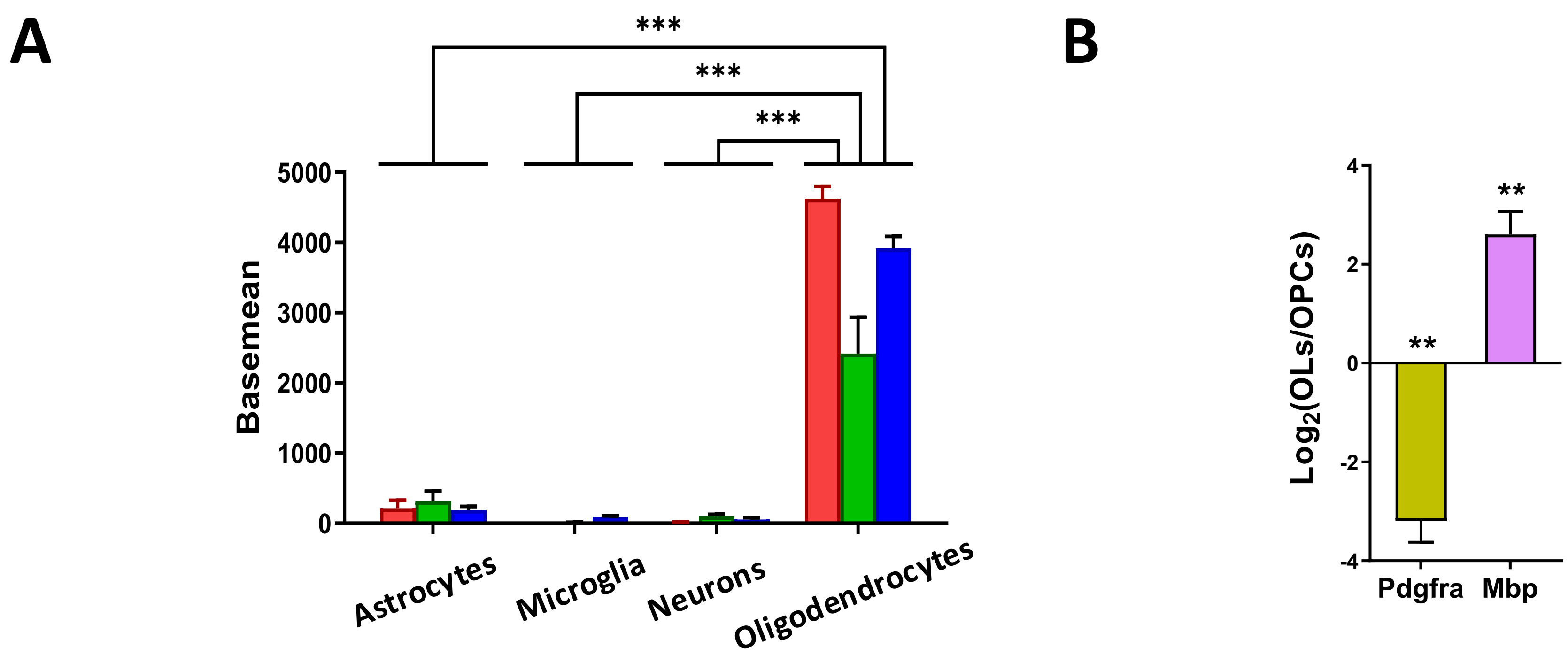

Appendix Fig. S3. Quality control of OL isolation protocol
A) Graph shows mean ± SEM expression of astrocyte- (Gfap, Aqp4, Aldh1l1), microglia- (Ccr9, Cd11b, Cd40), neuron- (Nefh, NeuN, Tuj1), and oligodendrocyte- (Cd82, Mobp, Cnp) specific genes across all RNA-seq samples, confirming minimal contamination by non-OL lineages (n = 12). Statistical analysis was performed using one-way repeated-measures ANOVA followed by Tukey's multiple comparisons test.
Expression values of the three markers corresponding to each cell lineage were averaged for each biological replicate (n = 12; $p_{astrocyte\text{- vs. oligodendrocyte-}} < 0.0001$, $p_{microglia\text{- vs. oligodendrocyte-}} < 0.0001$, $p_{neuron\text{- vs. oligodendrocyte-}} < 0.0001$, $p_{astrocyte\text{- vs. microglia-}} = 0.214$, $p_{astrocyte\text{- vs. neuron-}} = 0.164$, $p_{microglia\text{- vs. neuron-}} = 0.634$).

B) Validation of stage-specific OL enrichment: the graph shows the mean $\log_2$ fold change (purified OLs relative to OPCs) ± SEM for an OPC marker (Pdgfrα) and a mature OL marker (Mbp, used as a transcriptomic surrogate for O1, which is a ganglioside). Both markers exhibited significant differential expression between populations. As data did not meet the normality assumption (Shapiro-Wilk test), statistical analysis was performed using a two-tailed Mann–Whitney U test (n = 6; Pdgfrα: $p = 0.002$, Mbp: $p = 0.002$).

Asterisks in the graphs of panels denote significance levels: **$p < 0.01$; ***$p < 0.001$

APPENDIX FIGURE S4

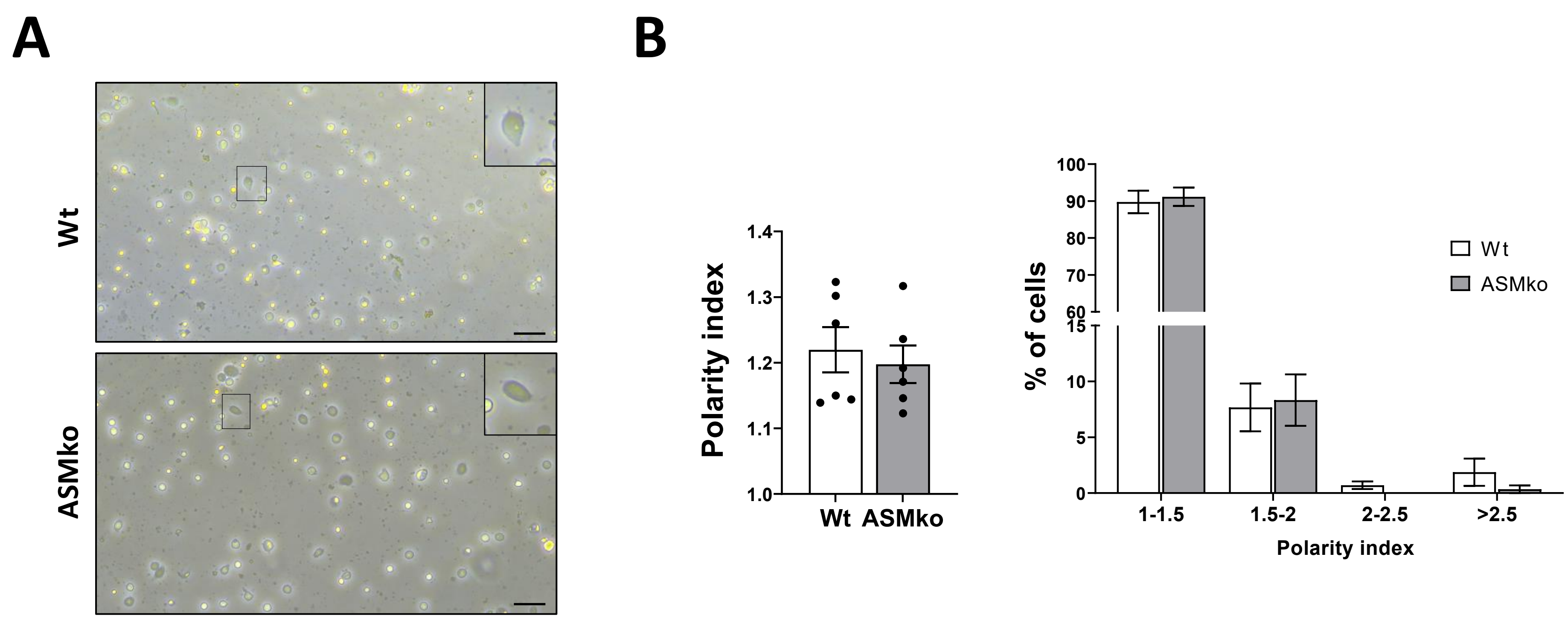

Appendix Fig. S4. Polarity index analysis of WT and ASMko OPCs during early adhesion to hyaluronic acid.
A) Representative phase-contrast images of WT and ASMko OPC cultures 4 h after plating on hyaluronic acid-coated coverslips; insets show magnified representative cells for each genotype.

B) Data show the mean ± SEM polarity index (left), and the mean ± SEM percentage of cells reaching the indicated polarity index (right). Statistical analysis was performed using two-tailed t-test (n = 6; p_{PI} = 0.633 for mean polarity index; distribution p-values: $p_{1\text{-}1.5}$ = 0.726, $p_{1.5\text{-}2}$ = 0.840, $p_{2\text{-}2.5}$ = 0.065; $p_{>2.5}$ = 0.256). Scale bar: 50 µm.

APPENDIX FIGURE S5

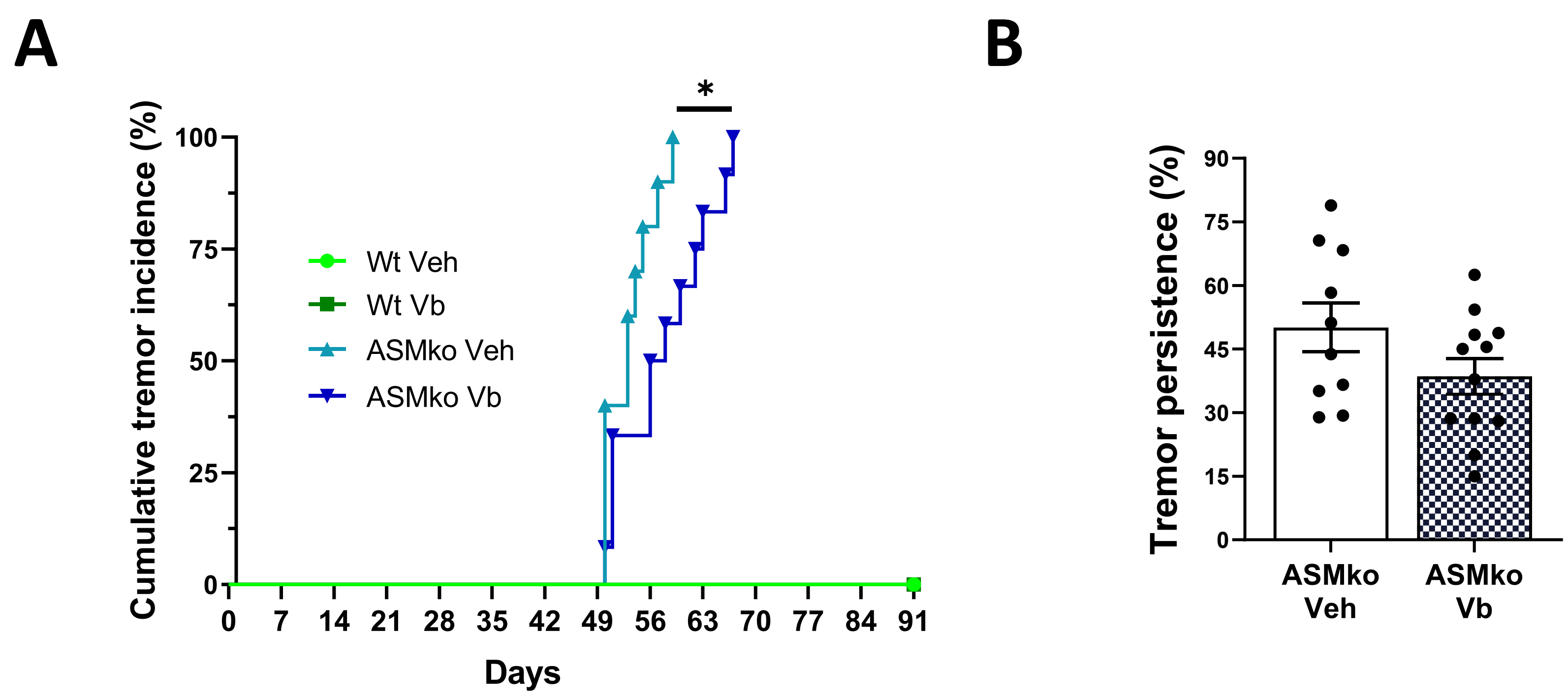

Appendix Fig. S5. Individual-level analyses of tremor onset and persistence in ASMko mice treated or not with Vb.
A) Cumulative incidence of tremors over time in ASMko mice, comparing vehicle- and Vb-treated groups. Time-to-event analysis was performed using Kaplan–Meier curves, where the event was defined as the first appearance of tremor in each animal (n = 10 for WT Veh, WT Vb and ASMko Veh; and n = 12 for ASMko Vb; Log-rank (Mantel-Cox) test *p = 0,018;* Gehan-Breslow-Wilcoxon test *p = 0,047*). Asterisk in the graph denotes significance levels: $*p < 0.05$.

B) Tremor persistence in individual mice, calculated as the percentage of days with tremor after first onset: (number of days with tremor / total days observed since first tremor) × 100. Data are shown as mean ± SEM (n = 10 for WT Veh, WT Vb and ASMko Veh; and n = 12 for ASMko Vb; Unpaired t test *p = 0,113*).

APPENDIX FIGURE S6

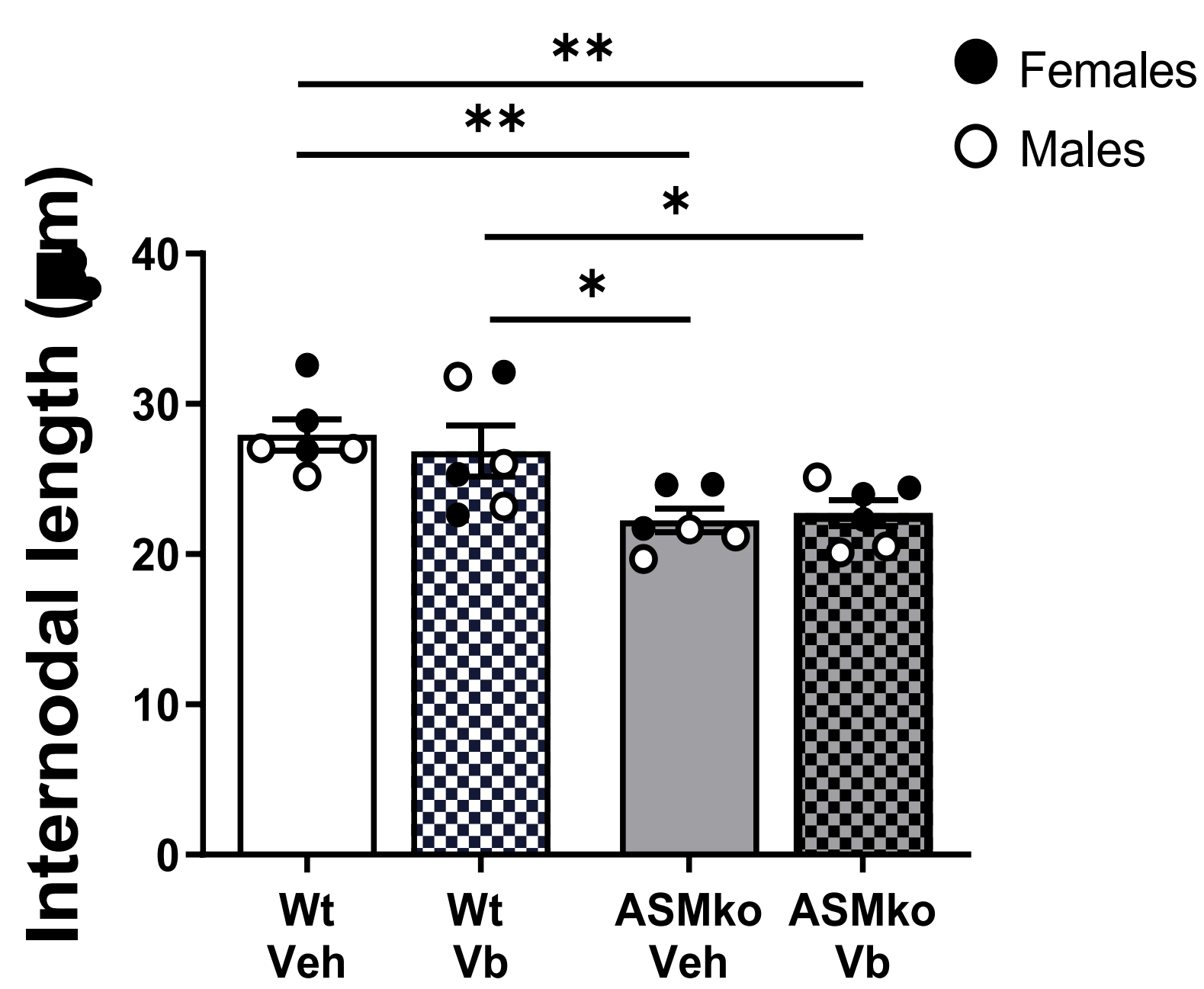

Appendix. Fig. S6. Quantification of internodal length in WT and ASMko mice treated or not with Vb.
Graph shows mean ± SEM internodal length in µm in the cerebellum of WT and ASMko mice treated with vehicle or with Vb (n=6). Black and white dots denote females and males, respectively; sex ratios were balanced. Statistics Ordinary one-way ANOVA followed by Sidak's post hoc comparisons test: $p_{ASMko\ Veh\ vs.\ Wt\ Veh} = 0.002$, $p_{Wt\ Vb\ vs.\ Wt\ Veh} = 0.510$, $p_{ASMko\ Vb\ vs.\ ASMko\ Veh} = 0.767$, $p_{ASMko\ Vb\ vs.\ Wt\ Veh} = 0.005$, $p_{ASMko\ Veh\ vs.\ Wt\ Vb} < 0.011$, $p_{ASMko\ Vb\ vs.\ Wt\ Vb} = 0.021$.
Asterisks denote significance levels: *$p < 0.05$; **$p < 0.01$.
