# Supplementary material for: Targeting CD44 reverses sphingomyelin-induced oligodendrocyte maturation arrest in acid sphingomyelinase deficiency
Source: EMBO Mol Med. 2026 Aug 12;18(9):3767–89. doi: 10.1038/s44321-026-00503-8 (PMC13562706; doi:10.1038/s44321-026-00503-8)
Supplement: Supplementary file 5 [file 44321_2026_503_MOESM3_ESM.zip › Figure 1/1F/Stages of MBP distribution during myelin formation.pdf]

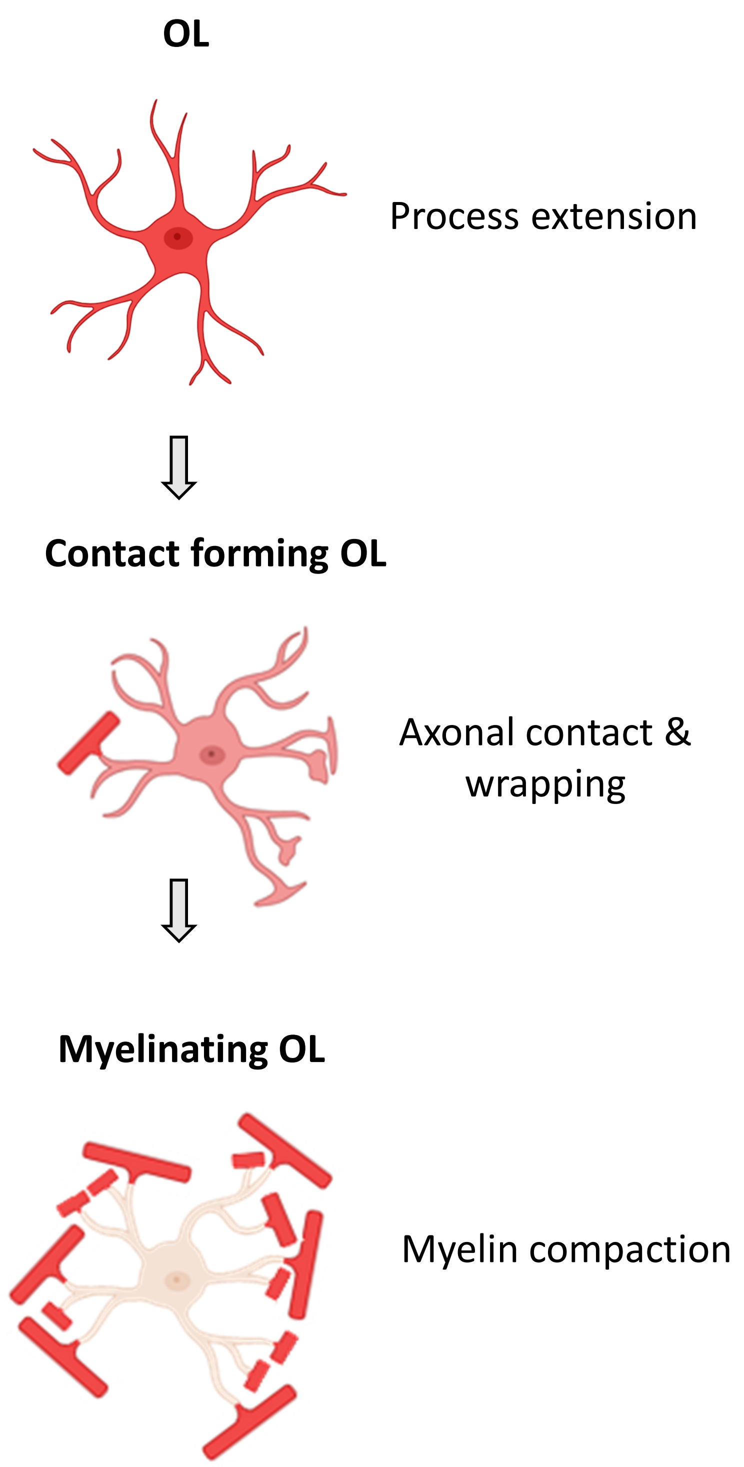
OL
Process extension
Contact forming OL
Axonal contact &
wrapping
Myelinating OL
Myelin compaction
