# Supplementary material for: Targeting CD44 reverses sphingomyelin-induced oligodendrocyte maturation arrest in acid sphingomyelinase deficiency
Source: EMBO Mol Med. 2026 Aug 12;18(9):3767–89. doi: 10.1038/s44321-026-00503-8 (PMC13562706; doi:10.1038/s44321-026-00503-8)
Supplement: Supplementary file 6 [file 44321_2026_503_MOESM4_ESM.zip › Figure 2/A/Esquema Linaje OLs.pptx [solo lectura].pdf]

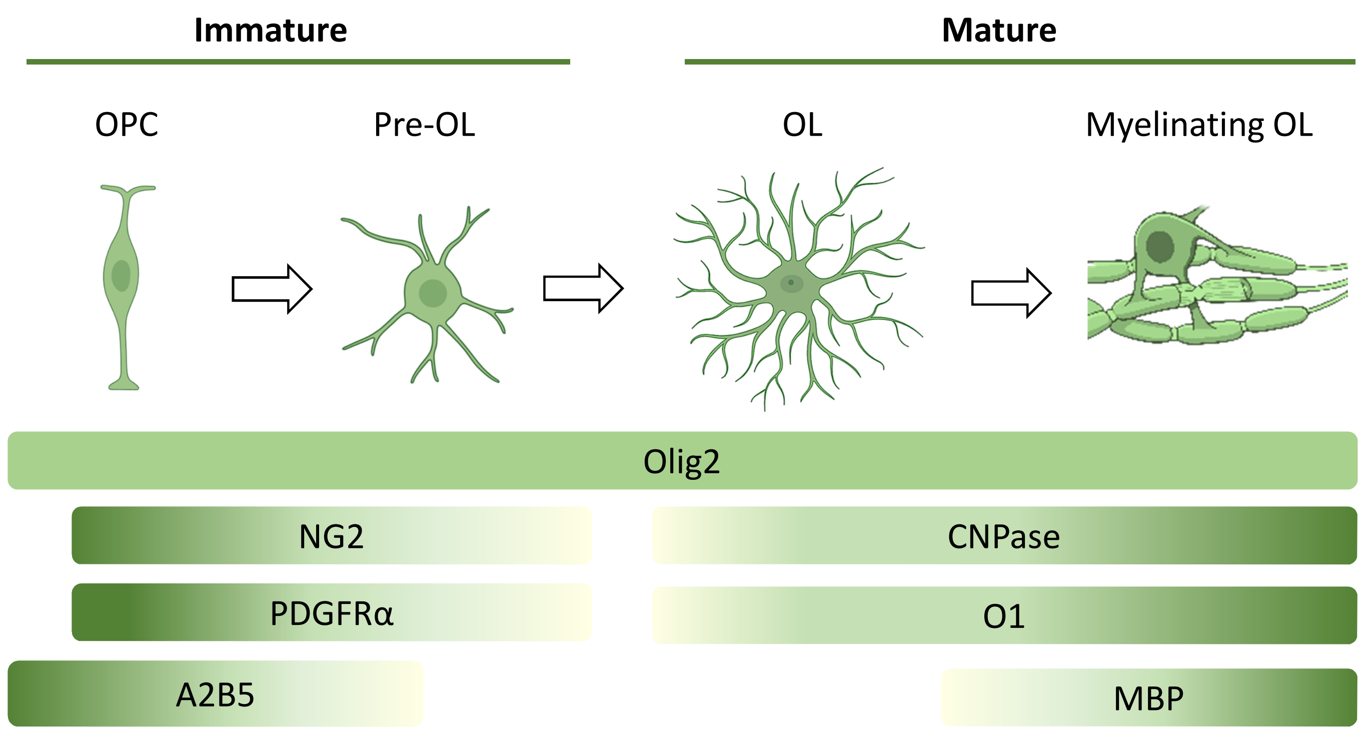
Immature
Mature
OPC
Pre-OL
OL
Myelinating OL
Olig2
NG2
CNPase
PDGFRα
O1
A2B5
MBP
