# Supplementary material for: Targeting CD44 reverses sphingomyelin-induced oligodendrocyte maturation arrest in acid sphingomyelinase deficiency
Source: EMBO Mol Med. 2026 Aug 12;18(9):3767–89. doi: 10.1038/s44321-026-00503-8 (PMC13562706; doi:10.1038/s44321-026-00503-8)
Supplement: Supplementary file 7 [file 44321_2026_503_MOESM5_ESM.zip › Figure 3/A/Workflow used to isolate OPCs and mature OLs from mice for RNA-seq analysis.pdf]

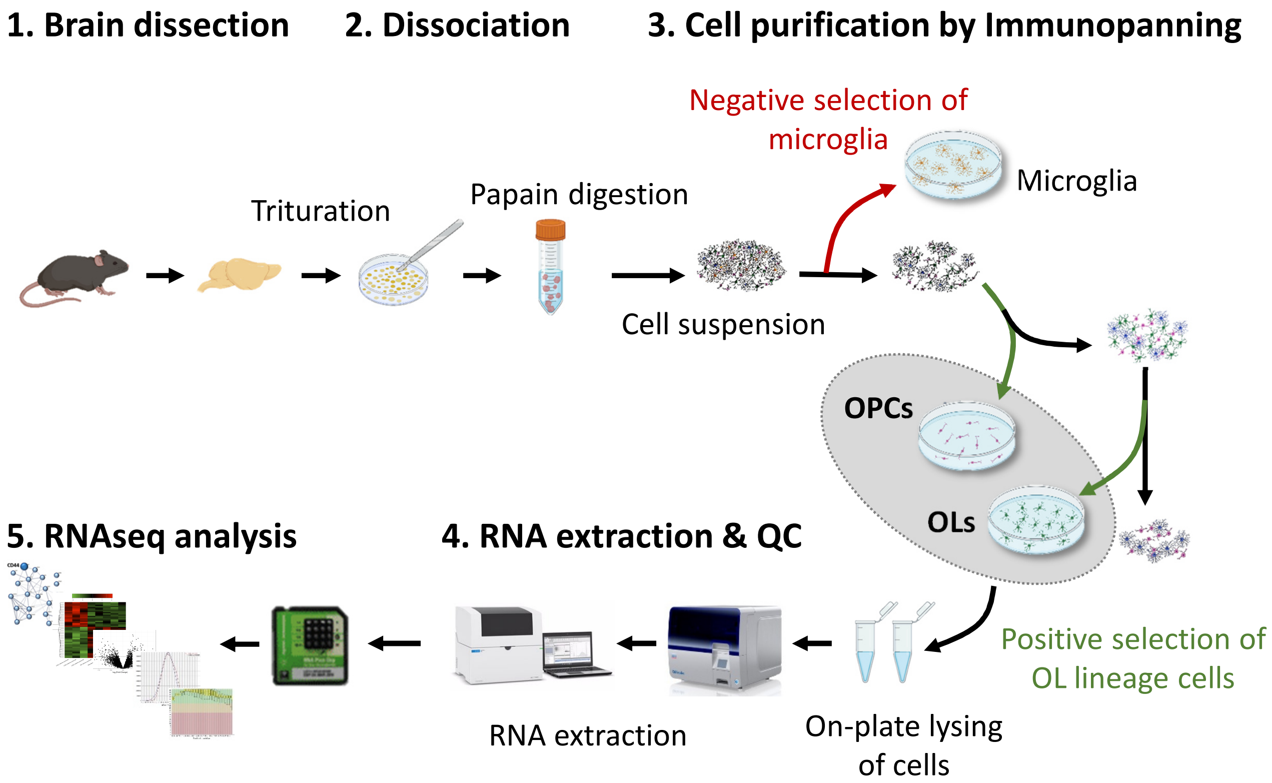
1. Brain dissection
2. Dissociation
3. Cell purification by Immunopanning
Negative selection of microglia
Microglia
Trituration
Papain digestion
Cell suspension
OPCs
OLs
Positive selection of OL lineage cells
On-plate lysing of cells
4. RNA extraction & QC
RNA extraction
5. RNAseq analysis
