# Supplementary material for: Targeting CD44 reverses sphingomyelin-induced oligodendrocyte maturation arrest in acid sphingomyelinase deficiency
Source: EMBO Mol Med. 2026 Aug 12;18(9):3767–89. doi: 10.1038/s44321-026-00503-8 (PMC13562706; doi:10.1038/s44321-026-00503-8)
Supplement: Supplementary file 7 [file 44321_2026_503_MOESM5_ESM.zip › Figure 3/E/OLs padj0.05 basemean50.pdf]

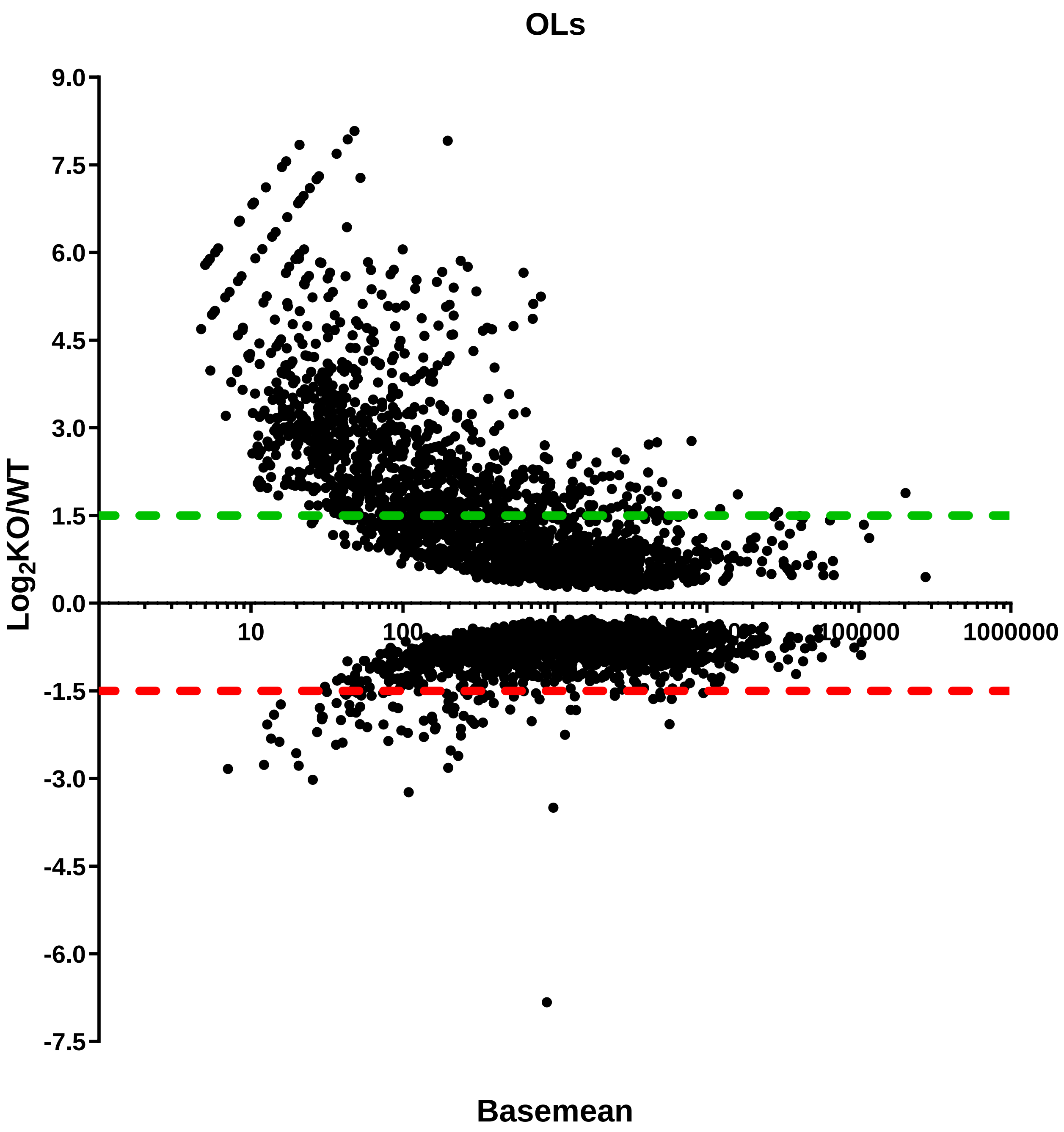
OLs
Log2KO/WT
9.0
7.5
6.0
4.5
3.0
1.5
0.0
-1.5
-3.0
-4.5
-6.0
-7.5
10
100
100000
1000000
Basemean
